# Whole-genome sequence and mass spectrometry study of the snow blight fungus *Phacidium infestans* (Karsten) DSM 5139 growing at freezing temperatures

**DOI:** 10.1007/s00438-023-02073-7

**Published:** 2023-10-10

**Authors:** C. Zerouki, K. Chakraborty, S. Kuittinen, A. Pappinen, O. Turunen

**Affiliations:** School of Forest Sciences, University of Eastern Finland, Yliopistokatu 7, 80101 Joensuu, Finland

**Keywords:** *Phacidium infestans*, *Gremmenia infestans*, Whole-genome sequence, Metabolomic enzymes, Cold adaptation, Mass spectrometry

## Abstract

*Phacidium infestans* (synonym *Gremmenia infestans*) is a significant pathogen that impacts *Pinus* species across the northern regions of Europe and Asia. This study introduces the genome sequence of *P. infestans* Karsten DSM 5139 (Phain), obtained through Pacbio technology. The assembly resulted in 44 contigs, with a total genome size of 36,805,277 bp and a Guanine–Cytosine content of 46.4%. Genome-mining revealed numerous putative biosynthetic gene clusters that code for virulence factors and fungal toxins. The presence of the enzyme pisatin demethylase was indicative of the potential of Phain to detoxify its environment from the terpenoid phytoalexins produced by its host as a defense mechanism. Proteomic analysis revealed the potential survival strategies of Phain under the snow, which included the production of antifreeze proteins, trehalose synthesis enzymes, desaturases, proteins related to elongation of very long-chain fatty acids, and stress protein responses. Study of protein GH11 endoxylanase expressed in *Escherichia coli* showed an acidic optimum pH (pH 5.0) and a low optimum temperature (45 °C), which is reflective of the living conditions of the fungus. Mass spectrometry analysis of the methanol extract of Phain, incubated at − 3 °C and 22 °C, revealed differences in the produced metabolites. Both genomic and mass spectrometry analyses showed the ability of Phain to adapt its metabolic processes and secretome to freezing temperatures through the production of osmoprotectant and cryoprotectant metabolites. This comprehensive exploration of Phain's genome sequence, proteome, and secretome not only advances our understanding of its unique adaptive mechanisms but also expands the possibilities of biotechnological applications.

**Supplementary Information:**

The online version contains supplementary material available at 10.1007/s00438-023-02073-7.

## Introduction

Coniferous trees are susceptible to various pathogenic fungi that can lead to severe tree damage and substantial losses. Among these, the fungus *Phacidium infestans* holds particular significance as a major pathogen that affects *Pinu*s species across northern Europe and Asia (Björkman 1948; Kujala 1950; Roll-Hansen 1989). This study focuses on revealing the genomic and metabolic cold-adaptation features of *P. infestans* (hereafter referred to as Phain), which is a common pathogen in the circumpolar boreal forest zone, where it triggers 'snow blight' by growing under the snowpack on infected seedling needles and killing them. The infection leads to the growth of grayish mycelium on seedlings, which results in brown needles post-snowmelt. Infection occurs via ascospores in autumn and persists until the snow covers the apothecia or until they are exhausted (Kurkela 1997). Previous observations in Finnish nurseries and inoculation trials suggest that Phain can also infect Norway spruce (*Picea abies*) seedlings (Lilja et al. 2010).

Previous studies have shown that the duration of snow cover affects fungal growth both in terms of water availability and because of the insulating properties of the snow (Schmidt et al. 2012). A thicker snow cover and later snowmelt have been related to massive needle loss in spruce trees (caused by snow fungi) and subsequent increased tree mortality rates (Auger and Payette 2010).

Under the snow, Phain also spreads from branch to branch by mycelial growth and the fungus is able to continue its growth at sub-freezing point temperatures (Björkman 1948). The mature spores continue to be released at temperatures close to 0 °C. It has been shown that free water is required to initiate ascospore release and maximum dispersal is reached within 4–6 h of suitable rain, although heavy rain can decrease spore liberation (Lilja et al. 2010). Under laboratory conditions, growth has been recorded as low as − 5 °C (Björkman 1948). According to Vuorinen and Kurkela (1993), carbon dioxide (CO_2_) concentrations and Phain activity under the snow reach a maximum between − 3 and 0 °C in late winter when the mycelial colonies achieve their greatest extension. Usually, snow blights occur in northern latitudes and at high altitudes in the south where there is sufficient snow cover during winter (Jamalainen 1956). However, it has been demonstrated that Phain was also able to infect seedlings in an indoor freezer store (Petäistö and Hantula 2013), which suggests that the disease could also develop in the absence of snow cover.

Effective control of snow blight in nurseries is performed by spraying the seedlings with fungicides. Moreover, overwintering outdoor seedlings should be sprayed as late as possible in the autumn before the formation of the permanent snow cover.

Phain grows on pine needles, which are known for their high terpene composition. Pine needles have been found to contain five times greater monoterpene concentrations than wood (Manninen et al. 2002). Terpenes are also known to have an antifungal effect (Withers and Keasling 2007). By 2007, more than 40,000 different terpenes had been identified (Withers and Keasling 2007).

Previous research has determined the chemical composition of essential oil from pine needles (*Cedrus deodara*) (Zeng et al. 2012). In total, 23 components have been identified by gas chromatography and mass spectrometry and represent 95.79% of the oil composition. The main components include α-terpineol (30.2%), linalool (24.47%), limonene (17.01%), anethole (14.57%), caryophyllene (3.14%), and eugenol (2.14%) (Zeng et al. 2012). Chemical analysis of the essential oil in Scots pine (*P. sylvestris*) (Mofikoya et al. 2020), for example, has demonstrated that the essential oils in the needles can strongly inhibit fungal spore germination and mycelial growth (Amri et al. 2013; Bakkali et al. 2008; Boulogne et al. 2012) by disrupting internal structures and permeabilizing fungal cells (Amri et al. 2011; Bakkali et al. 2008). Furthermore, Scots pine lesions infected by *Cronartium pini* fungus were found to show elevated terpene concentrations (Kaitera et al. 2021). Thus, fungi, such as Phain, that live in terpene-rich environments have developed mechanisms to survive in these conditions.

Given that Phain thrives under sub-zero temperatures beneath the snow cover and is a key pathogen of conifer trees, it is an appealing subject for comprehensive investigations, such as whole-genome sequencing and the exploration of secondary metabolites and enzymatic assemblies. In this context, the present study is designed to unravel the complete genome sequence of the fungus *P. infestans* Karsten DSM 5139, which was isolated from cedar pine (*Pinus cembra*) trees in the Austrian Alps. The enzymes, secondary metabolites, and adaptive mechanisms of Phain were investigated based on genome and proteome annotations. Furthermore, mass spectrometry analysis of the compounds secreted by Phain at 22 and − 3 °C was performed as a first step to investigate its cold-adapted metabolism.

## Materials and methods

### Strain cultivation

*P. infestans* Karsten DSM 5139 was purchased from the DSMZ collection. The fungus was originally isolated in 1982 from the needles of *P. cembra* in Pinzgau, Obersulzbachtal (47.1197N, 12.2948E), Austria.

The strain was cultivated on MEA-agar plates (pH 5.6) and incubated at 24 °C. To purify the DNA, the fungus was cultivated on liquid medium containing 2% malt extract (VWR Chemicals) by inoculating a disk (6 mm) of mycelium. The flasks were incubated for 3–6 days at 24 °C by shaking at 150 rpm.

### Genome DNA extraction

Fungal pellets were collected by filtration using Whatman No. 1 filter paper, followed by cell disruption. Glass beads (0.5 mm) from Scientific Industries Inc. were used to disrupt the cells. The beads were utilized with the Disruptor Genie^®^ from Scientific Industries SI™. Within the microtubes, a volume ratio of 40% disruptor beads to 40% fungus suspension was maintained, with approximately 20% headspace to facilitate the disruption process. The cell disruption procedure took place in a controlled cold room environment at 6 °C, utilizing pre-chilled materials. The disruption process was carried out at a speed of 2400 rpm for 5 min. The disrupted fungal material was then collected for DNA purification.

The genomic DNA was extracted using a Plant/Fungi DNA isolation kit (Norgen Bioteck Corp. (E5038-1KT)). The DNA concentration and quality were checked using NanoDrop^®^ ND-1000 from Thermo Scientific and Lonza FlashGel™ electrophoresis.

### Whole-genome sequencing

Initially, the Internal Transcribed Spacer (ITS) regions of the ribosomal DNA (rDNA) were sequenced from the fungus sample by Macrogen Europe (Amsterdam, The Netherlands). These highly variable sequences are of great importance in distinguishing fungal species by PCR analysis using the primers ITS1 (5′-TCCGTAGGTGAACCTGCGG-3′) and ITS4 (5′-TCCTCCGCTTA TTGATATGC-3′) (White et al. 1990).

The fungal DNA was initially purified from the fungus cultivated in 2% malt extract liquid medium with a Plant/Fungi DNA Isolation Kit (Sigma-Aldrich E5038). The whole-genome sequencing of the isolated DNA was carried out at the sequencing center of the University of Oregon (USA) Genomics & Cell Characterization Core Facility (GC3F) by Pacific Biosciences Sequel II technology (PacBio). The fungal DNA was further purified with the YeaStar Genomic DNA Kit (Zymo Research). The DNA was introduced into SMRTbell libraries using the Express Template Prep Kit 2.0 from PacBio according to the manufacturer’s protocol (https://www.pacb.com/wp-content/uploads/Procedure-Checklist-Preparing-Multiplexed-Microbial-Libraries-Using-SMRTbell-Express-Template-Prep-Kit-2.0.pdf). Samples were pooled into a single multiplexed library and size selected using the Sage Sciences BluePippin system with the 0.75% DF Marker S1 High-Pass 6–10 kb v3 run protocol.

A size selection cutoff of 8000 (BPstart value) was used. The size-selected SMRTbell library was annealed and bound according to the SMRT Link Set Up, and sequenced on Sequel II. Raw PacBio reads were converted to fasta format with Samtools fasta (http://www.htslib.org/doc/samtools.html) and were then assembled with Flye 2.6 (https://github.com/fenderglass/Flye) with parameters-plasmids-iterations 2 and asm-coverage 120.

### Genome analysis

The assembled fungal genome was annotated using Augustus (https://github.com/Gaius-Augustus/Augustus). Protein annotation was performed using BlastKOALA (Kanehisa et al. 2016). The predicted protein sequences were blasted in Geneious against the UniProt/Swiss-Prot database at different E-values (10, 1e − 1). Cluster of Orthologous Groups (COG) annotations were performed using the WebMGA server, and KofamKOALA for KEGG Orthology (Aramaki et al. 2020). Blast2GO (Conesa et al. 2005) was used to blast search the nonredundant database of 4751 taxa of fungus UniProt/Swiss-Prot sequences with E-value 1e−3 (word size 6). The Interproscan search was carried out in Galaxy/Europe (Afgan et al. 2016).

Carbohydrate-active enzymes (CAZy) were investigated using the dbCAN meta server (Zhang et al. 2018), and HMMER, Diamond and Hotpep tools were utilized. The secondary metabolite biosynthetic gene clusters (BGC) were determined by the antiSMASH fungal version (Blin et al. 2019).

### Enzymatic study of endoxylanase GH11

#### Gene cloning

The protein sequence of putative GH11 endoxylanase (Phain_OT5_Proseq9131) obtained from the whole-genome sequences was fused with the N-terminal *Bacillus amyloliquefaciens* pelB secretion signal sequence and synthesized by Genscript from the deduced amino acid sequence. The expression construct DNA provided by Genscript, and optimized for *Escherichia coli* expression, was transformed into competent *E. coli* BL21 (DE3) cells purchased from Merck (CMC0014-4X40UL). The transformed cells were spread onto LB-agar ampicillin plates (pH 7). Colonies were selected and grown in Lysogeny Broth (LB) medium with 100 mg ampicillin to obtain crude enzyme.

#### Enzyme production and assays

Enzyme production was induced by 1.0 mM IPTG after overnight incubation at 30 °C (pH 7) in LB medium. The flasks were then incubated by shaking for at least 4 h. A clear solution was obtained by eliminating the cells by centrifugation. Enzymatic activity was evaluated using 1% birchwood xylan (Carl Roth GmbH) as a substrate using the dinitrosalicylic acid method (DNS) modified from Bailey et al. (1992). Absorbance was measured by a spectrophotometer at the 540 nm wavelength.

Determination of the pH activity profile at 40 °C for xylanase was carried out at pH values that ranged from pH 3 to 8. Two different buffers were used: 50 mM citrate–phosphate buffer (pH 3–7), and 50 mM potassium phosphate buffer (pH 8). Determination of the optimal temperature for the enzymes was carried out by incubating the enzyme for 30 min at temperatures that ranged from 25 to 55 °C, at pH 4.5. Each data point was the mean value of three assay times (replicates).

### Mass spectrometry analysis LC–MS/MS

#### Solvent extraction

Mass spectrometry analysis was performed on the Phain samples to identify the metabolites secreted at different temperatures. Phain was inoculated on 10 MEA plates (pH 5.9) and incubated at 22 °C until sufficient growth was observed. Five of those plates were placed at − 3 °C for 14 days and the other five were kept at 22 °C. After incubation, the mycelia were scraped from the agar surface and used as raw material for metabolite extraction. In total, 0.5 g of each sample were scraped on ice and dissolved in methanol. The samples were then extracted by shaking for at least 4 h at 6 °C. The solid materials were then removed by centrifugation and the supernatants were transferred into clean tubes. The same extraction steps were performed on the MEA plates (without the fungus) as a control. All the extracts were dried using a SpeedVac vacuum concentrator. The samples were then sent to MS-Omics, Denmark for mass spectrometry analysis.

#### Quality control and sample preparation

Quality control on the samples was performed at MS-Omics as follows: A mixed pooled sample (QC sample) was created by taking a small aliquot from each sample. This sample was analyzed at regular intervals throughout the sequence. Matrix effects were tested to quantify the compounds by spiking the QC sample at a minimum of two levels. The samples were resuspended in 200 μl methanol and incubated for 1 h on ice. The volume was increased to 450 μl, and 200 μl samples were taken and filtrated through spin X columns with 100 μl transferred to high recovery vials for injection. Analytical performance of UPLC–MS analysis was determined on the basis of a quality control (QC) sample, made from pooled equal aliquots from each sample. Over the sequence, this pooled QC was measured as every 7th injection, resulting in a total of seven repeated analyses. Analytical variation of metabolite responses (peak areas) was determined as the standard deviation in the pooled QC and reported normalized to the average area for each metabolite expressed as percentage (Relative precision).

#### Analysis method

Analyses of the samples were carried out by MS-Omics using a Thermo Scientific Vanquish LC coupled to a Thermo Q Exactive HF mass spectrometer. An electrospray ionization interface was used as an ionization source. Analyses were performed in negative and positive ionization modes. Each sample was extracted once and analyzed once in positive and negative mode. The best signal for each metabolite (either positive or negative) was selected and its relative response was reported.

The Ultra Performance Liquid Chromatography (UPLC) was performed using a slightly modified version of the protocol described by Hsiao et al. (2018). Peak areas were extracted using Compound Discoverer 3.1 (Thermo Scientific). Identification of compounds was performed at four levels: Level 1: identification by retention times (compared against in-house authentic standards), accurate mass (with an accepted deviation of 3 ppm), and a tandem mass spectrometry spectrum (MS/MS spectra); Level 2a: identification by retention times (compared against in-house authentic standards), accurate mass (with an accepted deviation of 3 ppm); Level 2b: identification by accurate mass (with an accepted deviation of 3 ppm), and MS/MS spectra; Level 3: identification by accurate mass alone (with an accepted deviation of 3 ppm).

The statistical significance of the mean values of the studied compounds in the two treatment groups (− 3 and 22 °C extracts, five replicates at each temperature) was evaluated using a t-test (two tails, type 3).

## Results

### Genomic features

PacBio sequencing generated a total read length of 11,917,952,680 bp with a coverage of 238. The N50/N90 read values were 11,945/7514. The genome assembly resulted in 44 contigs (Table 1) for a total genome size of 36,805,277 bp and a Guanine–Cytosine (GC) content of 46.4%. The length, coverage, and numbers of the contigs and scaffolds of the assembled genome are shown in Table S1. The BUSCO analysis (Nishimura et al. 2017) showed 98.6% completeness: (C: 98.6% [S: 97.9%, D: 0.7%], F: 0.7%, M: 0.7%, n: 290). The BUSCO analysis also indicated that 97.9% of the genes were single copy and 0.7% were duplicated genes.

**Table 1:** Genome sequence features of *Phacidium infestans* DSM 5139

Main genome scaffold total	44
Main genome contig total	44
Main genome scaffold sequence total	36.805 MB
Main genome contig sequence total (0.000% gap)	36.805 MB
Main genome scaffold N/L50^a^	8/1.729 MB
Main genome contig N/L50^a^	8/1.729 MB
Main genome scaffold N/L90^b^	22/618.358 KB
Main genome contig N/L90^b^	22/618.358 KB
Max scaffold length	4.329 MB
Max contig length	4.329 MB

^a^N**/**L50 is defined as count of the smallest number of contigs or scaffolds whose length sum makes up half the genome size

^b^N/L90 is defined as the smallest number of contigs or scaffolds whose length sum makes up 90% of the genome size

### Similarity search

**Fig. 1:**
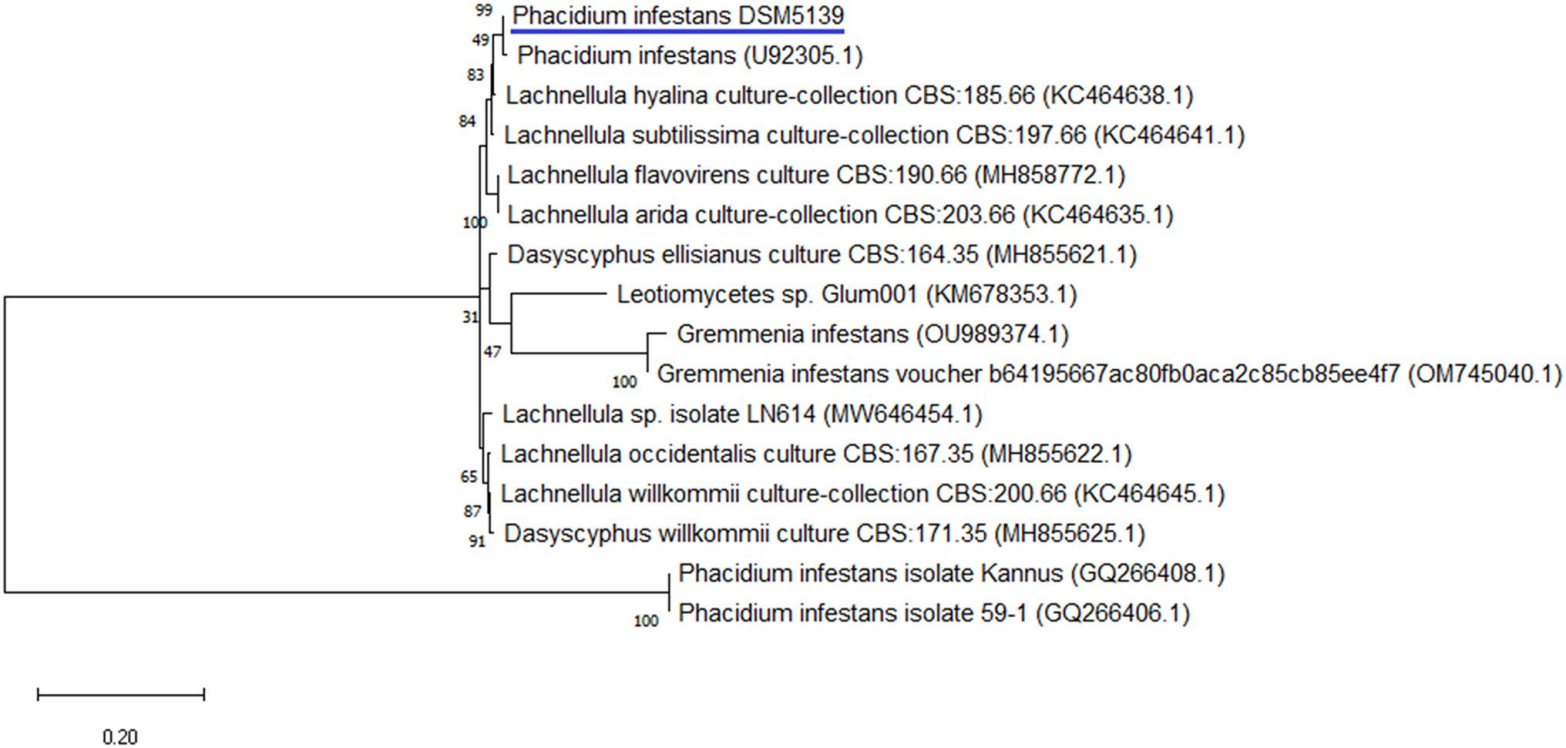
Phylogenetic analysis of DSM 5139 using Internal Transcribed Spacer (ITS) sequences with Mega 11. Model K2P, (+G) was used based on 1000 Bootstrap replications

To analyze the molecular classification and phylogeny of Phain DSM 5139, the DNA sequences of ITS1 and ITS4 were obtained from Macrogen Europe (Amsterdam, The Netherlands). The cleaning of the sequences resulted in 865 bp for ITS1 and 876 for ITS4. The sequences were then subjected to a BLAST search on NCBI Nucleotide, and the most relevant hits were utilized to construct a phylogenic tree. The phylogenic analysis was performed using Mega 11. The Kimura 2-parameter (K2P) model was used, incorporating a discrete Gamma distribution to model evolutionary rate differences among sites (+G) and 1000 replications (Fig. 1).

Strain DSM 5139 was successfully clustered with the *P. infestans* (U92305.1) strain with 99 bootstrap replications. On different branches of the same clade, *L. subtilissima* CBS: 197.66 and *L. hyalina* CBS: 185.66 were found among other *Lachnellula* strains that were supported by 49 bootstrap replications.

The Blast2GO search, which used UniProt/Swiss-Prot from 4751 species of fungi, generated 9920 hits and is illustrated in Fig. S1. In 4631 hits, identity was assigned to *L. subtilissima*, followed by 2631 hits assigned to *L. hyalina*, 479 hits to *L. cervina*, 470 hits to *L. arida,* 407 hits to *L. occidentalis,* 308 hits to *L. willkommii,* and 251 hits to *Cudoniella acicilaris* (Fig. S1).

Based on these results, the nearest sequenced fungal genome was the *L. subtilissima* strain CBS: 196.66, which is a wood-associated saprobe fungus with a total genome size of 33.86 Mb (11,875 proteins). Thus, the six nearest species in the whole-genome sequence comparison belonged to the *Lachnellula* genus.

Average nucleotide identity (ANI) was estimated for Phain with *L. subtilissima* CBS:196.66 (Lacsu), *L. hyalina* CBS:185.66 (Lachy), and *L. willkommii* GCA_007825375.1 (Lacwi) (names used in this study are shown in parentheses) by comparing the genome sequences in the ANI Calculator (http://enve-omics.ce.gatech.edu/ani/index) using the following alignment parameters: minimum length 700 bp, minimum identity 70%, minimum alignments 50, fragment options 1000 bp for window size, and 200 bp for step size. The results using best hits (one-way ANI) and reciprocal best hits (two-way ANI) resulted in hits of 87.19% ANI between Phain and Lacwi, 91.82% hits between Phain and Lachy, and 92.01% hits between Phain and Lascu (Fig. S2). These results confirmed the Blast2GO findings and indicated that Lacsu is the closest relative to Phain at the genomic and protein levels.

To access the genetic similarity, a digital DNA–DNA hybridization (dDDH) was conducted using Genome to Genome Distance Calculator (GGDC) (https://ggdc.dsmz.de/) available from the Leibniz Institute DSMZ. The probability that DDH > 70% is indicative that the analyzed genomes are from the same species. The Phain genome was compared to three *Lachnellula* fungi. The results indicated a dDDH estimation of 42.50% between Phain and *L. subtilissima* CBS: 196.66, 41.70% dDDH between Phain and *L. hyalina* CBS:185.66, and 27.90% dDDH between Phain and *L. willkommii* (GCA_007825375.1). These results clearly showed that Phain and Lacsu are distant species.

### Protein analysis

The genome assembly revealed 11,357 open-reading frames (ORF) with a length range of 55–8028 amino acids. The shortest detected ORF with only 16 amino acids long was excluded from this list. Out of the 27 sequences that contained an X, 51 protein sequences were generated, and 24 were renamed and added to the end of the protein sequence list. Thus, the final number of protein sequences was 11,381. In total, 279 of the proteins were 55–99 amino acids long, and 10,501 proteins had a length of 150–8028 amino acids. The final protein sequences were then analyzed with various tools to accurately predict their functions, structures, and protein relationships.

#### Protein annotations

The functional annotation was carried out based on a similarity search to known sequences using BlastKOALA (Kanehisa et al. 2016). The results indicated 3847 entries. The functional annotation KofamKOALA included HMMER/HMM SEARCH against Kofam (a customized HMM database of KEGG Orthologs (KO)) (Aramaki et al. 2020) and generated 4194 entries. In total, 39% of 10,000 proteins were identified with a length of 186–8028 amino acids. However, only 21.4% of 11,381 proteins with a length of 55–186 amino acids were annotated.

To identify and characterize the sequences with similarities to a query sequence, thereby inferring potential functions and evolutionary relationships, a Blast search using the UniProt/Swiss-Prot databases was performed on Geneious software. In total, 9710 hits were generated with an E-value of 10 and a word size of 6. Of these, 2388 proteins had only 15–99 amino acids long regions with similarity, while 5763 proteins had 200–4820 amino acids long regions with similarity. The identity in the matching region was 18.3–100% for the whole group.

InterProScan, conducted within Galaxy Europe, was utilized to provide a comprehensive assessment of a potential functions and structural attributes of the protein. Of the total, 10,026 proteins exhibited similarity to proteins that harbored established features, with a significant number demonstrating an association with cellular membranes. Furthermore, 3588 membrane-associated proteins with domains predicted to be extracellular, 2532 proteins with a membrane-associated cytoplasmic domain, and 2703 proteins with a membrane-bound domain embedded in the membrane. In total, 1317 proteins were identified with a signal peptide region.

#### Truncated proteins

In most cases, the sequences before and after the in-frame stop codon (in 27 proteins) showed significant shared identity with known proteins, which would suggest that a genomic rearrangement fused the two protein sequences close to each other. In two cases, the stop codon was found at the beginning of the protein, and then, the protein was not split into two proteins. About half of the resulting 51 sequences were estimated to be partial (truncated) proteins and about 11 were likely to be full-length proteins. These findings suggest that genomic reorganization happened to some degree in Phain.

### GH11 xylanase

**Fig. 2:**
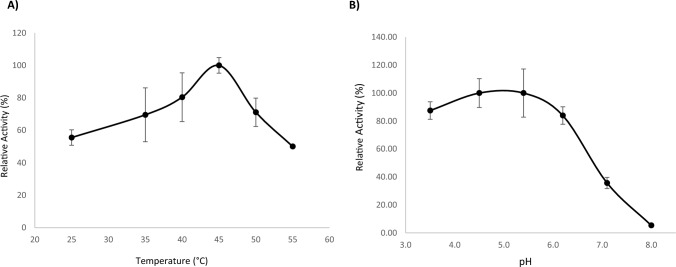
Temperature and pH assay of the *Phacidium infestans* GH11 xylanase*.* **a** Temperature activity profile at pH 4.5 determined with 30-min enzyme assay, and **b** pH activity profile at 40 °C

Characterization of GH11 xylanase was utilized to unveil the range of pH and temperature conditions in which the fungus grows. The partial characterization of xylanase GH11 indicated mesophilic behavior with a temperature optimum at 45 °C and activity even at 55 °C (Fig. 2a), which is almost similar to the cold-active *Cladosporium neopsychrotolerans* SL-16 xylanases that showed temperature optima at 35–40 °C (Ma et al. 2018). This reflects the ability of Phain to grow even at quite high temperatures. Optimal enzymatic activity was observed at pH 5.0. However, GH11 xylanase also seemed to be active at quite low pH values (Fig. 2b). The enzyme became inactive at pH 8. These results indicated that the fungus grew at mildly acidic pH values.

## Comparison of *P. infestans* and *Lachnellula* proteomes

**Fig. 3:**
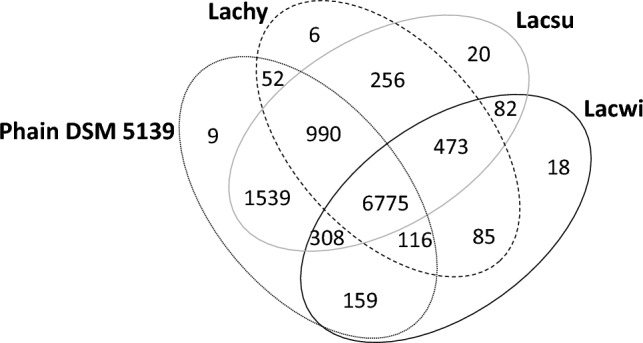
Venn diagram based on protein sequences of Phain, Lacsu (CBS: 196.66), Lachy (CBS: 185.66) and Lacwi (GCA_007825375.1) constructed using OrthoVenn2

A comparative analysis was conducted between Phain and *Lachnellula* fungi using the OrthoVenn2 platform. This online resource enabled the identification and comparison of protein clusters across distinct strains. The Venn diagram (Fig. 3) showed that 6775 protein clusters were common to all strains. Phain and Lacsu have 1539 unique clusters that are shared only by these two strains, whereas the mutual unique clusters between the other strain pairs were much lower (Fig. 3). These results confirmed that phylogenetically, Lacsu is the closest genome to Phain among the studied fungi. This finding also supported the phylogenetic order generated by Blast2GO. Phain and Lacsu had 9612 common protein clusters, Phain had 220 unique protein clusters, and Lacsu had 831 unique protein clusters. In addition, Phain had 1290 species-specific singleton proteins that do not belong to any cluster, while Lacsu had 1086 singletons, Lachy had 272, and Lacwi had 803.

The comparison of Phain to a phylogenetically distant strain, such as *Trichoderma reesei* QM6a (9111 proteins), is shown in Table S2. The number of shared protein clusters between those two strains was 5692, while only 2550 protein clusters were shared between Phain and *Saccharomyces cerevisiae* (6002 proteins) (see Table S2). The species-specific singleton proteins were most likely involved in the creation of the species-specific features, whereas the common proteins were largely responsible for basic metabolic and cellular functions, which are largely common between the species. Lacsu contained a greater number of proteins than the other compared species (Table S2), with this difference seen in most of the protein classes.

The Phain genome was found to contain a group of 26 reverse transcriptase-like proteins that seemed to have started from one parent copy and then spread in the genome. The proteins Phain_OT5_Proseq8679 and Phain_OT5_Proseq 8681 were identified as retropolymerases.

### Analysis of singleton proteins

To identify and characterize proteins that exist as single occurrences within the dataset, the singleton proteins were subjected to BLAST search using Uniprot/Swiss-Prot database by Geneious (word size 6, Blosum62). In total, 1290 Phain-specific singleton proteins (size distribution 55–2450 amino acids) were obtained from the OrthoVenn2 comparison with *Lachnellula* species (Lacsu, Lachy and Lacwi). In addition, nine Phain-specific clusters (19 proteins) were also identified as exclusive to Phain (Fig. 3). Only the highest identity matches were recorded for each protein. The results showed that 870 hits (from 1309 proteins) were obtained with 15–1624 aligned region lengths: 385 proteins (29.4% of proteins) had at least an 80 amino acid long matching region with a protein detected by Blast. As expected, there were no sequence matches with the *Lachnellula* species.

#### Secondary metabolite analysis

The prediction and annotation of the biosynthetic gene clusters (BGC) responsible for secondary metabolites production within the genome were conducted using AntiSMASH (version 7 beta). In total, 34 regions were identified as BGC in Phain. Of these, 11 regions were attributed as Nonribosomal Peptide Synthetases (NRPS) and NRPS-like gene clusters, 11 regions as a T1PKS cluster and one region as a T3PKS cluster. A total of eight fungal-RiPP-like BGC were found. Of the 34 BGC, only eight clusters showed similarity with known compounds (Table 2) and a 100% hit was obtained with three gene clusters. Two BGC coded for UNII-YC2Q1O94PT and choline and a third BGC that showed a 100% hit was identified as a biosynthetic gene of phenguignardic acid, or aspulvinone H/aspulvinone B1. However, the identity of the cluster was not clear (for phenguignardic acid, see Molitor et al. 2012). A 50% similarity match was detected with the PR-toxin synthesis BGC, and 40% similarity was detected with the squalestatin S1 biosynthesis gene cluster. AntiSMASH attributed one cluster to gregatin A (55%). Fujikurins A, B, C, and D were also attributed to the same region as gregatin A with a 66% hit. Fujikurin and fujikurin-like gene clusters are also called PKS19 in *F. fujikuroi* and the phylogenetic analysis showed that the PKS19 gene cluster was widespread among phytopathogens and plant-associated fungi.

**Table 2:** AntiSMASH (V.7.0.0 Beta) prediction of Biosynthetic gene clusters (BGC) of *Phacidium infestans* showing similarity with known BGC

Type	From^a^	To^b^	Most similar known cluster (% ID)	
NRPS-like	101,002	140,948	Choline	100
T1PKS	334,334	381,683	Trichoxide	50
NRPS-like	1,814,662	1,857,516	Aspulvinone H/aspulvinone B1 Phenguignardic acid	100
T1PKS	1,696,453	1,745,120	Gregatin A	55
			Fujikurin A, B, C, and D	66
Terpene	1,675,310	1,696,453	PR-toxin	50
NRPS	1,079,128	1,123,204	UNII-YC2Q1O94PT	100
NRPS	987,101	1,040,243	Nidulanin A	50
Terpene	146,522	168,230	Squalestatin S1	40

^a^First nucleotide of the biosynthetic gene cluster

^b^Stop codon sequence of the biosynthetic gene cluster

Interproscan annotation identified 132 cytochrome P450 proteins. The Geneious Blast of *P. infestans* proteins identified 17 polyketide synthase proteins that were over 2000 amino acids in size. These proteins are listed in Table S3. Also, 9 NRPS or NRPS-like single proteins and 12 NRPS-like fragments were found from the Geneious blast search.

The functional annotation KofamKOALA identified 315 protein clusters with 454 proteins involved in the biosynthesis of secondary metabolites. However, only one of the 17 polyketide synthases detected by Blast were among the proteins found by KofamKOALA. By screening the matches found with the Geneious blast using various secondary metabolites as screening key words, ~ 690 proteins (size > 100 amino acids) were detected and, thus, could be involved in secondary metabolite synthesis. However, this number did not contain transcription regulators. In addition, at least 84 were truncated proteins and at least 77 were fused or larger proteins. While about 190 of the non-truncated proteins were transporters, hundreds of enzymes have the potential for biosynthesis of secondary metabolites. Most of the identified pathway genes are located in different places and only in a few cases was there significant clustering of BGC into the same contig region.

There are numerous proteins in Phain that show partial shared identity with the synthesis proteins present in other fungi, e.g., for the production of pisatin, botcinic acid, fumonisin, citrinin, fusicoccii A, cyclochlorotine, brefeldin A control, abscisic acid, aflatoxin, dothistromin, pneumocandin, calbistrin, pyriculol/pyriculariol, cyclochlorotine, phomenoic acid, fusicoccin A, and tropolone (some are shown in Table S3). However, the large numbers of proteins needed for full synthesis pathways were not identified in most of the potential clusters. Thus, it is possible that many of these clusters are defective (Ulrich et al. 2020).

### Secondary metabolites in phytopathogenesis

The Geneious Blast identified many gene clusters involved in the phytopathogenesis of Phain. Specifically, a fujikurin cluster was identified within a unique location, specifically contig_3, as a series of genes. This cluster encompassed most of the required enzymes for fujikurin biosynthesis. Thus, it is probable that Phain possesses the capability to synthesize fujikurins. Phain_OT5_Proseq6436 protein shared 63% identity with Type 1 (T1PKS) Polyketide synthase PKS19 of *F. fujikuroi,* which is responsible for fujikurin synthesis (von Bargen et al. 2015). Two other enzymes in Phain showed over 60% shared identity with the known fujikurin synthesis enzymes of *F. fujikuroi* IMI 58289 (Table 3).

**Table 3:** Fujikurin gene clusters (PKS19) identified in *Phacidium infestans* using Geneious Blast

Sequence name/sequence length (amino acid)	Proteins^a^	Best hit^b^	Identity (%)
Phain_OT5_Proseq6436 (2469 aa)	Highly reducing polyketide synthase	Fujikurins biosynthesis cluster protein PKS19	63
Phain_OT5_Proseq6435 (346 aa)	Trans-enoyl reductase	Fujikurins biosynthesis cluster protein FFUJ_12240	67
Not identified	Hydrolase	Various hydrolases exist	
Phain_OT5_Proseq6431 (413 aa)	Putative transcription factor	Fujikurins biosynthesis cluster transcription factor FFUJ_12243	37
Phain_OT5_Proseq3693 (1637 aa)	Putative transporter	Fujikurin efflux protein	45
Phain_OT5_Proseq6433 (557 aa)	Cytochrome P450 monooxygenase	Low identity with various biosynthesis proteins	27–29
Phain_OT5_Proseq6434 (294 aa)	Polyketide transferase	Fujikurins biosynthesis cluster protein FFUJ_12241	68
Phain_OT5_Proseq6439 (269 aa)	Short chain dehydrogenase	Trichoxide biosynthesis protein virK	48

^a^Proteins retrieved from the fujikurin synthesis pathway in *F. fujikuroi* (Studt et al. 2016)

^b^Most similar biosynthesis protein available in the database

The analysis of phytopathogen metabolite production in Phain revealed the presence of the aristolochene synthase enzyme. This enzyme catalyzes the cyclization of farnesyl diphosphate (FPP) to the bicyclic sesquiterpene aristolochene, which is a starting compound for the synthesis of a number of sesquiterpenoid toxins produced by filamentous fungi (Cane and Kang 2000). This key enzyme identified in the Phain genome (Phain_OT5_Proseq8148; 340 amino acids) shared 68% identity with the aristolochene synthase (Q9UR08) of *Aspergillus terreus*, and 70.2% sequence identity to the *A. terreus* aristolochene synthase protein structure (PDB structure 3bnx)*.* A model created by Swiss-Model showed that the active site is fully conserved in the Phain enzyme, thereby confirming that the enzyme function apparently is also fully conserved in Phain. A partial pyriculol pathway was found in Phain by the Geneious Blast and appeared to be similar to the *Pyricularia oryzae* pyriculol synthesis pathway.

In addition, several pisatin demethylase candidates were found in Phain. This enzyme found in phytopathogenic fungi plays a critical role in their interaction with host plants. The protein Phain_OT5_Proseq9413 had the highest detected similarity to the pisatin demethylases: 98% shared identity with the pisatin demethylase of Lacsu (TVY38707.1).

## Pathogenesis-related proteins

The investigation of pathogenesis-related proteins revealed the potential involvement of three proteins in Phain pathogenesis. The UniProt Blast search identified the protein Phain_OT5_Proseq5572 (170 aa) as a putative pathogenesis-related protein with 96.3% shared identity with the same protein in Lacsu (A0A8H8RXT0). Protein Phain_OT5_Proseq6172 (161aa) was identified by the UniProt Blast as a putative pathogenesis-related protein that had 44.7% shared identity with *Ceratocystis fimbriata* CBS 114723 (A0A2C5X2F9). *C. fimbriata* is a phytopathogen ascomycete native to Brazil and is the causative agent of wilting and canker on numerous hosts (Oliveira et al. 2018).

The protein Phain_OT5_Proseq9013 (980aa) was identified as a virulence protein that had 99.2% shared identity with the *L. hyalina* virulence protein (XP_031001133) encoded by the gene *Ssd1*. The *Ssd1* homologues have been found to be important for the virulence of fungal pathogens of plants and humans (Thammahong et al. 2019).

Three oxalate decarboxylases were identified in Phain. The BLAST search of proteins Phain_OT5_Proseq9876 (499 aa) and Phain_OT5_Proseq5870 (464 aa) indicated 98.20% and 98.71% shared identity, respectively, with the oxalate decarboxylases VY34405.1 and TVY40836.1 of Lacsu*.* Phain_OT5_Proseq1811 protein (414 aa) showed 95.69% shared identity with the putative oxalate decarboxylase TVY36189.1 of *L. occidentalis*.

## Cold adaptation

The capacity of Phain to withstand freezing conditions was subjected to investigation. Several proteins associated with cold adaptation were identified by analyzing the Geneious Blast results. The identified proteins were further analyzed by Blast on NCBI. The Phain_OT5_Proseq6240 protein was identified as a protein related to the elongation of very-long-chain fatty acids (3-keto acyl-CoA synthase ELOVL3), which is a cold-inducible glycoprotein of 30 kDa size. The Phain_OT5_Proseq5092 protein showed similarity to the cold-sensitive fermentation protein 1 (CSF1) of *Saccharomyces cerevisiae* S288C (Q12150) but with a low shared identity (28%).

Two Phain proteins were identified as antifreeze proteins. Phain_OT5_Proseq9011 (779 aa) was identified as the IBP AFP1. The NCBI Protein BLAST search showed a 60.6% shared identity region with the IBP of *D. vulcani* (WP_198925670.1; 316 aa). In *S*. *frigidimarina*, the protein functions as an ice-binding adhesion protein that maintains the favorable position of this strain in its aquatic habitat (Vance et al. 2018).

The second IBP similarity was found with Phain_OT5_Proseq10963 (220aa). The UniProt database search indicated 32% shared identity with the antifreeze protein K3-B1 in the gray snow mold *Typhula ishikariensis* (Q76CE6). This enzyme binds to the surface of ice crystals and lowers the freezing point of an aqueous solution (Cheng et al. 2016).

**Fig. 4:**
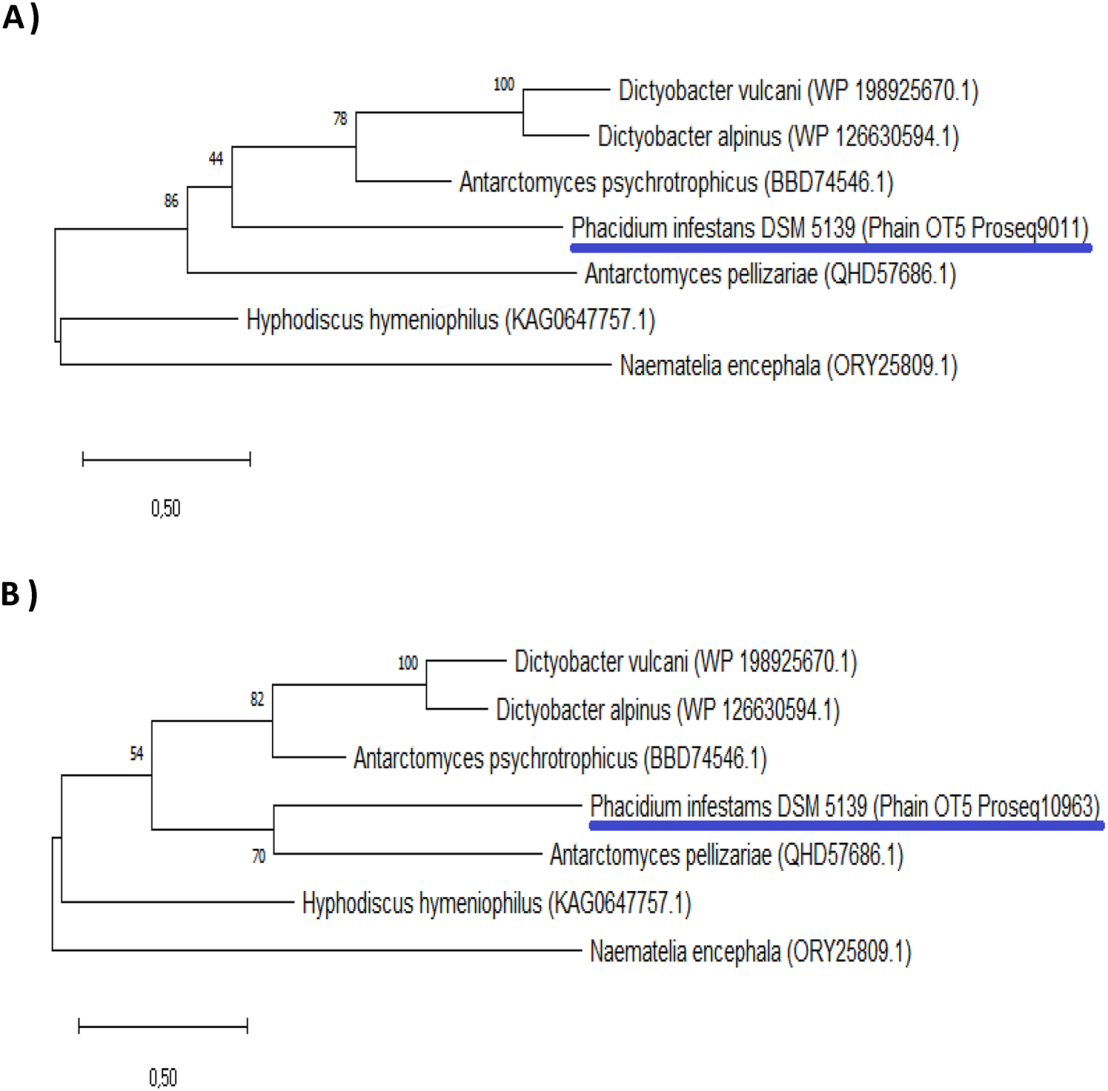
Phylogenetic tree based on Maximum Likelihood method using amino acid sequences of ice-binding proteins from various fungi. Bootstrap values are indicated at the branch points. The model was based on WAG + G + F and 1000 replications. **a** Phylogenetic analysis of the IBP Phain_OT5_Proseq9011. **b** Phylogenetic analysis of the IBP Phain_OT5_Proseq10963

The phylogenetic analyses of the IBPs encoded by the genes Phain_OT5_Proseq9011 and Phain_OT5_Proseq10963 were performed with six other sequences of IBPs from different fungi deposited in NCBI database. The protein Phain_OT5_Proseq9011 appeared phylogenetically distinct from other IBP proteins (Fig. 4a). On the other hand, the IBP Phain_OT5_Proseq10963 was successfully clustered with the IBP of the ascomycete blue stain fungus *Antarctomyces pellizariae* (IBP accession: QHD57686.*1*) (Fig. 4b) supported by 70 bootstrap. *Antarctomyces pellizariae* strain was recovered from the seasonal snow in the Antarctic Peninsula by de Menezes et al. (2017).

In addition to the antifreeze proteins, trehalose synthesis enzymes were detected: trehalose-6-phosphate synthase (Tps1) and trehalose-6-phosphate phosphatase (Tps2). Phain_OT5_Proseq343 (511 aa) was identified as alpha, alpha-trehalose-phosphate synthase (78% shared identity with *A. niger* protein Q00075)*.* Phain_OT5_Proseq547 was identified as trehalose-6-phosphate phosphatase (Tps2) (962 amino acids) with a 44% shared identity with the *S. cerevisiae* protein P31688. In total, seven potential desaturases (length range 345–617 amino acids) were found in Phain with 46–99% shared identity with known desaturases (Phain_OT5_Proseq238, Phain_OT5_Proseq2676, Phain_OT5_Proseq4847, Phain_OT5_Proseq4931, Phain_OT5_Proseq5217, Phain_OT5_Proseq8468, Phain_OT5_Proseq10124).

## Stress proteins

To identify the stress proteins of Phain, Swiss-Prot database search was performed on Geneious, and revealed the presence of 17 stress-related proteins. The Swiss-Prot search attributed the Phain_OT5_Proseq8537 to cell wall integrity and stress response with a 39% shared identity with protein Q12215 of *Saccharomyces cerevisiae* S288C. Phain_OT5Proseq471 was also identified as cell wall integrity and stress response. The search indicated 97.2% shared identity with the protein TVY32721.1 of Lacsu. The cell wall integrity (CWI) pathway helps to fortify and repair cell wall damage in stressful environments.

The protein Phain_OT5_Proseq5771 shared a 98.9% shared identity with Target of Rapamycin (TOR) complex 2 subunit of Lacsu (Protein accession TVY32766.1). Phain_OT5_Proseq1309 and Phain_OT5_Proseq3339 were identified as stress response element-binding proteins and were studied for their involvement in the osmotic stress response in *T. atroviride* (Peterbauer et al. 2002). Our results showed 40% and 32% shared identity between Phain_OT5_Proseq1309 and Phain_OT5_Proseq3339, respectively, and the stress response element-binding proteins of *Aspergillus fumigatus* Af293 (Q4WPF5). Four putative stress response proteins nst1 (Phain_OT5_Proseq7825, Phain_OT5_Proseq6335, Phain_OT5_Proseq1771, Phain_OT5_Proseq7993) and one putative peroxide stress-activated histidine kinase mak1 (Phain_OT5_Proseq2500) were identified. Putative mitogen-activated protein kinase (MAPK) was also found (Phain_OT5_Proseq1552).

Several superoxide dismutase related proteins were identified in Phain (Phain_OT5_Prose5443, Phain_OT5_Proseq2671, Phain_OT5_Proseq6247, Phain_OT5_Proseq1964). Those proteins showed a strong shared identity (97.7–100%) with the known proteins in *Lachnellulas* and, thus, indicate the ability of Phain to withstand oxidative stress. One thioredoxin reductase enzyme was identified in Phain (Phain_OT5_Proseq2352), and two putative catalase–peroxidase enzymes (Phain_OT5_Proseq2735, Phain_OT5_Proseq2238), and three catalases (Phain_OT5_Proseq9152, Phain_OT5_Proseq1952, Phain_OT5_Proseq4412) were also identified.

The Phain_OT5_Proseq9060 protein was identified as glutathione peroxidase-like peroxiredoxin HYR1 (97.84% shared identity with Lacsu TVY34082.1).

## Mass spectrometry analysis

Mass spectrometry analysis indicated the presence of numerous compounds produced by Phain at both 22 and − 3 °C. Of those compounds, special interest was paid to those listed in Table 4 and Table S5. Annotation level 1 indicated the presence of betaine as a major compound in all the analyzed samples (smaller amount in the control) and slightly more in the − 3 °C samples. However, the observed difference between these two extracts was not statistically significant (*p*-value: 0.54). The Phain genome sequence indicated the presence of enzymes related to betaine metabolism (Phain_OT5_Proseq1467, Phain_OT5_Proseq3181, Phain_OT5_Proseq2261). These proteins were identified as betaine aldehyde dehydrogenases. In addition, the protein Phain_OT5_Proseq8866 was identified as choline monooxygenase with 70.5% shared identity to KAI9926982.1 of *Aspergillus wentii*. Choline monooxygenase catalyzes the first step of the glycine betaine synthesis.

**Table 4:** Mass spectrometry results of the selected compounds produced by Phain at − 3 °C and 22 °C

Monoisotopic mass	Formula	Compound (annotation level)	− 3 °C average area, ± standard deviation	22 °C average area, ± standard deviation	Control^a^ (area)	*p*-value^b^
226.10	C10 H14 N2 O4	Carbidopa (level 2b)	1,603,064 ± 370,612	521,044 ± 262,797	9976	0.004
237.09	C9 H11 N5 O3	Sepiapterin (level 2b)	6,875,365 ± 1,891,958	2,373,722 ± 1,371,746	2076	0.003
253.08	C9 H11 N5 O4	Neopterin (level 2b)	7,637,449 ± 2,402,794	3,599,159 ± 2,471,979	3872	0.03
117.08	C5H 11N O2	Betaine (level 1)	1,654,148,902 ± 478,694,055	1,481,382,680 ± 355,974,675	2,336,773	0.54
161.11	C7 H15 N O3	Carnitine (level 1)	2,154,586 ± 1,622,666	1,568,248 ± 681,028	41,428	0.49
342.12	C12 H22 O11	Hexose dimer (level 1)	3,431,904 ± 2,713,679	6,026,936 ± 5,548,518	422,496	0.38
152.07	C5 H12 O5	Sugar alcohol (level 1)	9,687,053 ± 20,331,693	11,545,816 ± 10,995,786	20,757	0.25

^a^Unit used is relative area (areas under the curve/ chromatographic peak)

^b^Calculated from the difference between mean values of the − 3 °C and 22 °C extracts

Mass spectrometry analysis showed the presence of carnitine (annotation level 1) at both temperatures. Four carnitine-related enzymes (94.09–100% shared identity) were also identified in the proteome of Phain using Uni-Prot Blast (Phain_OT5_Proseq5001, Phain_OT5_Proseq4716, Phain_OT5_Proseq5268, and Phain_OT5_Proseq9590).

The presence of dihydrolipoamide was greater in the − 3 °C extracts than in the 22 °C extracts. The difference in dihydrolipoamide at both temperatures was statistically significant (*p*-value: < 0.001). Uni-Prot indicated the presence of many proteins that could be potentially associated with dihydrolipoamide. Protein Phain_OT5_Proseq236 was identified as dihydrolipoamide dehydrogenase (DLD) (74.53% shared identity with PMD14080.1) and is a part of the pyruvate dehydrogenase (PDH) complex and plays an important role in the production of energy for cells.

Sepiapterin and neopterin were found to be two- to threefold more abundant in the −3 °C extracts. The difference in the detected pterins was highly significant for both compounds (*p*-value: < 0.05). At the proteome level, Phain_OT5_Proseq8549 appeared to be a molybdopterin synthase catalytic subunit (98.39% shared identity with TVY36555.1). Protein Phain_OT5_Proseq3354 was identified as gephyrin (100% shared identity with TVY41082.1), which is a multifunctional protein responsible for molybdenum cofactor biosynthesis (Tyagarajan and Fritschy 2014). As an example, an unidentified hexose dimer and sugar alcohol (Table 4), and other similar metabolites were detected at comparable levels at both temperatures, highlighting the significance of compounds differing between the two temperatures.

## Discussion

*P. infestans* is known as a hygrophilic and cryophilic fungus (Roll-Hansen 1989). This strain has been previously studied for its ability to kill pine needles under the snowpack. The strain DSM 5139 with the designation P431 was isolated in 1982. It has been studied and compared to other *Phacidium* species by its growth rate as function of temperature and the microscopic aspect including ascomata, ascospores and asci by Butin and Söderholm (1984). The genome sequencing of *P. infestans* Karsten. DSM 5139 resulted in 44 contigs, 36,805,277 bp and 98.6% BUSCO completeness. The initial phylogenetic study based on ITS sequences indicated that the reference strain *P. infestans* (GenBank accession U92305.1) was the most identical fungus to DSM 5139 with 100 bootstrap replications. U92305.1 is the same strain as Phain DSM 5139 that was purchased from the DSMZ collection. *P. infestans* U92305.1 sequence was obtained and deposited in the databank by Gernandt et al. (1997) with the strain designation P431.

The phylogenetic analysis showed that *Lachnellula* represents the closest genus available in the databases to DSM 5139 (48 bootstrap replications). The results based on the genomic comparison of the sequences (available in the databases) indicated that Lacsu (CBS:196.66) is the closest relative, followed by Lachy (CBS:185.66) and Lacwi (GCA_007825375.1). *Lachnellula* is a genus of fungi that causes canker disease in conifer trees. This genus belongs to the *Hyaloscyphaceae* family and contains 40 species (Oguchi 1981; Kirk et al. 2008). Lacwi is a fungus that causes European Larch Canker. Lacwi kills the growing bark, which results in swellings on the twigs and branches, and the formation of sunken cankers on the larger stems (Sinclair and Lyon 2005). Lacsu is a common wood-associated fungus of conifers of the *Pinaceae* family, especially *Pinus* and *Picea* trees (Kuo 2020). The fungus has been occasionally reported to cause minor damage to natural tree stands and plantations (Weissenberg 1975), to *Pinus contorta* in the nurseries of Finland and cause cankers on conifers in the Himalayas (Minter 2005).

Molecular analyses report this fungus to be abundant in the topsoil of coniferous stands where the fruitbodies are not observed (Baldrian et al. 2012). Lacsu is considered as an example of a saprotroph that can grow on multiple substrates due to the composition of its enzymatic machinery.

The DNA sequence similarity of Phain with the other *Lachnellula* fungi might be related to their ability to infect or grow on similar host trees, mainly conifers, thus suggesting host-specificity of these strains.

The Phain genome sequence was investigated for proteins and CAZymes using different bioinformatics tools. The protein GH11 xylanase was selected for enzymatic studies. The GH11 enzyme (Phain_OT5_Proseq9131) produced in competent *E. coli* cells showed an optimum temperature at 45 °C and optimum pH at 5.0. In the acidic GH11 xylanases, a residue position nearby acid/base catalyst is Asp on the edge of the active site canyon and it is Asn (position 71 in Phain) in mildly acidic-neutral enzymes (Li and Turunen 2015). In Phain, this position is occupied by Asn, which would suggest that the only detected GH11 xylanase of Phain is not strongly acidic. The pH optimum at 5.0 shows that Phain grows in mildly acidic conditions. Previous studies on *Duganella* sp., an Antarctic soil bacterium investigated for its novel cold-adapted endoxylanase, showed the greatest beechwood xylan-degrading activity occurred at pH 5.5 and at 40 °C (Kim et al. 2021). Aside from GH11, Phain also seems to contain a large number of putative glycoside hydrolases with many involved in fungal penetration of the plant cell wall (CAZymes), and a large number also known to be implicated in virulence (Rafiei et al. 2021).

Conifers accumulate resins in response to injury or infections. Pine produces oleoresin, which is a super-saturated solution of resin acids in liquid terpenes, and parenchyma resins composed of terpenes and terpenoids, as well as fatty acids (Peter 2018). Terpenes form a major class of compounds that show antifungal and antimicrobial properties.

Pine needle essential oils have been studied and have been found to exhibit antifungal activity, e.g., against *Fusarium* spp. (Krauze-Baranowska et al. 2002). Phain is a microorganism that survives on needles and kills them. In our study, secondary metabolite predictions showed the presence of 34 potential BGC. These clusters were attributed to various toxins, such as UNII-YC2Q1O94PT and the PR-toxin. The former has been characterized in the *Alternaria* producing host-selective ACR-toxin, which causes leaf spot disease in rough lemon (*Citrus jambhiri*) (Izumi et al. 2012). Snow blight kills needles under the snow and the disease produces small black dots of fruiting bodies in two lines on either side of the midrib, on the underside of the needles. Therefore, the ACR toxin could be involved in the formation of the brown spots on pine needles, since the spots are similar to those caused by *C. jambhiri* (Izumi et al. 2012). Furthermore, gregatin A (55% identity in Phain) is a fungal metabolite that was originally isolated from the fungus *Cephalosposium gregatum* in 1975 and has been found to be produced by several different fungal species (Kotayashi and Ui 1975). Studies on gregatins have shown their potential inhibitory activity against the opportunistic pathogen *Pseudomonas aeruginosa* (Beenker et al. 2022).

Various secondary metabolites are also involved in fungal phytopathogenesis, survival, and competition (Elhamouly et al. 2022). The study of secondary metabolites using the Geneious Blast highlighted the presence of various pathways potentially implicated in Phain virulence. Among those, the almost complete fujikurin pathway was identified in Phain. Studies on *F. fujikuroi* IMI 58289 BGC (PKS19) involved in fujikurin production were up-regulated during the infection of rice plants, highlighting a possible role as a phytopathogenic virulence determinant (Wiemann et al. 2013).The PKS19-like polyketide synthases are also widely distributed in phytopathogenic and plant-associated fungi (Sbaraini et al. 2017). Thus, fujikurins and fujikurin-like compounds appear to have a significant role in phytopathogenesis of the fungi that exhibit those genes.

The partial pyriculol pathway discovered in Phain could potentially contribute to its virulence. The proteins implicated in this pathway are listed in Table S4. Pyriculol was initially isolated from the rice blast fungus *Magnaporthe oryzae* and was found to induce lesion formation on rice leaves. The biosynthesis of pyriculol in *M. oryzae* showed that *RED1*, *RED2* and *RED3* and two putative oxidase-encoding genes (also identified in Phain) are particularly implicated in pyriculol biosynthesis (Jacob et al. 2017). Genome-mining studies have shown that the capability of fungi to produce secondary metabolites is underestimated. Many biosynthesis gene clusters are dormant under standard cultivation conditions and a very large number of natural products probably remain to be discovered (Brakhage 2013). Many proteins were found to have similarities to the biosynthesis proteins of several secondary metabolites. However, a less than 40% shared identity does not clearly indicate the biosynthesis role. Further studies are necessary for the identification of these secondary metabolites, with chemical identification of the secondary metabolites, in particular, critical to elucidate the role of the detected secondary metabolite synthesis proteins.

Plants produce low-molecular weight compounds known as phytoalexins, which have antimicrobial properties in response to microbial attack. The first phytoalexin to be identified was pisatin from peas (*Pisum* sp.). It is a fungistatic isoflavonoid produced by the garden pea (*P. sativum*), which is a host for *N. haematococca*. Many fungi, including *F. oxysporum* f. sp. pisi, can detoxify pisatin to a less inhibitory compound. This detoxification is catalyzed by demethylation of the compound by pisatin demethylase (cytochrome P450 enzyme) (Wasmann and Coleman 2022), which detoxifies the plant defense proteins and, thus, functions as a virulence factor for the fungus (Khan et al. 2003). Although, large numbers of cytochrome P450 proteins are likely to be involved in secondary metabolite biosynthesis.

The identification of several potential pisatin methylases in Phain indicates the ability of Phain to survive by degrading and detoxifying its environment from the phytoalexins produced by its host. Previous studies support this finding and show that pathogenic fungi, such as white-rot basidiomycetes, *Heterobasidion parviporum* (Martínez-Iñigo et al. 2000) and others, have developed the ability to survive in the presence of major compounds produced by their common hosts by detoxifying those compounds or even exploiting them as carbon sources (Kusumoto et al. 2014).

Our investigation of the pathogenic factors associated with Phain showed the presence of three oxalate decarboxylases. Oxalic acid/calcium oxalate could quench the calcium ions released during cell wall breakdown of the host plant, thus protecting the growing hyphae from toxic calcium concentrations in the infected area (Heller and Witt-Geiges 2013). Previous studies on the fungal oxalate decarboxylase showed the implication of these enzymes in the early infection of *S. sclerotiorum*. Furthermore, the S. *sclerotiorum* isolates inoculated on *Brassica juncea* cv. RL-1359, for example, showed that the isolates that produced the greatest amount of oxalic acid were the more aggressive, and vice versa (Gill et al. 2021). The identification of the oxalate decarboxylase enzymes in Phain might indicate that its phytopathogenesis involves the accumulation of high levels of oxalic acid.

Plants produce reactive oxygen species (ROS) to counteract pathogen invasion. The phytopathogens use scavenging strategies, such as catalases, to promote infection and pathogenicity (Yuan et al. 2021). Thus, catalase is considered as a virulence factor, e.g*.,* in *Aspergillus* species (Rouein et al. 2020). Target of Rapamycin (TOR) protein was detected in Phain. The TOR signaling pathway regulates growth in response to nutrient availability and stress in eukaryotic cells and the pathway is involved in the regulation of the extracellular secreted protein composition of the white-rot fungus *Phanerochaete chrysosporium* (Nguyen et al. 2020).

Cold environments are characterized by temperatures below 5 °C and represent a harsh habitat for microbial life (Eskandari et al. 2020). However, snow molds, such Phain, can grow and attack plants at low temperatures under the snowpack. Cold tolerance is one of the main factors related to their geographic distribution. Snow molds develop mycelia under the snow cover and produce intra- and extracellular enzymes that are active at low temperatures. To survive, fungi have developed a variety of mechanisms for cold tolerance, including the production antifreeze proteins, glycopeptides, and peptides. These proteins allow the microorganisms to live in sub-zero conditions and function by lowering the freezing point of water and avoiding the growth of ice crystals in the frozen stage (Davies et al. 2002; Eskandari et al. 2020). The first ice-active fungal protein (∼25 kDa) was found in a snow mold, *Typhula ishikariensis* and previous studies on cold-adapted fungi, including snow mold *T. ishikariensis*, *Lentinula edodes* (*s*hiitake mushroom), and *Flammulina populicola* (enoki mushroom), have shown that their IBPs share 50–55% identities and are similar to other IBPs identified in ice bacteria and sea ice diatoms. Moreover, previous research has indicated the possibility that some IBP genes may have spread by horizontal gene transfer (Raymond and Janech 2009).

The cold-adaptation strategies are not fully understood. In yeasts, cold survival includes the production of antifreeze and cold-active proteins, compatible solutes, and an increase in membrane fluidity (Hassan et al. 2016; Gunde-Cimerman et al. 2014). In Antarctic fungi, incubation at low temperatures resulted in an increase in antioxidant enzymes activities, which would suggest that antioxidant defense could also play a significant role in microbial survival when subjected to cold conditions (Gocheva et al. 2009; Kostadinova et al. 2012).

The enzyme elongase, associated with very long-chain fatty acids, was found in Phain. This protein is a rate-limiting enzyme that catalyzes the elongation of saturated and monounsaturated long-chain fatty acids. Functional characteristics of this enzyme in mammals suggest an ability to elongate C16 to C18. This enzyme is involved in adaptation to low temperatures (Chen et al. 2018).

The trehalose pathway was identified in Phain, and appears to be a general stress protectant in the cytosol, and is known to stabilize membranes during dehydration (Goodrich et al. 1988; Robinson 2001). Several studies have shown that trehalose accumulates in fungal hyphae in response to low temperatures*.* Genes associated with cold tolerance in the yeast *Naganishia vishniacii,* isolated from Antarctica, were found to code for solute transfers, chaperones, were associated with photoprotection, trehalose synthesis, lipid metabolism, and desaturases (Nizovoy et al. 2021). Seven desaturases were detected in Phain. These proteins are involved in the modification of fatty acids in response to changing temperature conditions (Czumaj and Śledziński 2020).

The protein CSF1, also known as bridge-like lipid transfer protein family member 1, was identified in Phain. CSF1 is a fungal membrane protein that is required for fermentation at low temperatures. Previous research on CSF1 showed that this protein is associated with a nutrient transport system that exists on the plasma membrane and is required only at low temperatures (Masaya et al. 2000).

The stress response investigation in Phain indicated the presence of proteins implicated in the MAPK pathway. The MAPK cascade results in the activation of transcription factors and the expression of specific sets of genes in response to environmental stimuli. The MAPK pathways have important roles in fungal physiology, development, in stress response, UV radiation, temperature changes, virulence, cell–cell signaling, and fungus–plant interaction (Martínez-Soto and Ruiz-Herrera 2017).

In our study, the mass spectrometry analyses performed on methanol extracts of Phain incubated at − 3 and 22 °C showed a clear variation in some of the secondary metabolites produced. It appears that Phain adapts its metabolism to its living conditions. On the other hand, few of the metabolites were found to react to temperature and, thus, signify the ability of Phain to stabilize some of its metabolites under changing environmental conditions.

Evading oxidative stress is crucial for fungi to survive. Oxidative stress can result from exposure to biotic and artificial agents in nature or from the immune cells during host attack. Such stress, when coupled with antioxidant systems, can lead to fungal death (Yaakoub et al. 2022). In addition to oxidative stress responses, it has been shown that cryoprotectants, such as sugars, alcohols, and amino acids, are produced in large amounts to prevent cold-induced aggregation of proteins. These compounds maintain optimum membrane fluidity under unfavorable low temperatures. Previous research on *Listeria monocytogenes* at low temperatures has identified the growth-enhancing effect of glycine betaine (Ko et al. 1994). Betaine/glycine (C5H11NO2) is widely distributed among organisms. The role of betaine is to protect microbial cells against drought, osmotic stress, and temperature stress (Zou et al. 2016).

In our study, carnitine was detected by mass spectrometry analysis and on the Phain proteome. Carnitine is involved in energy metabolism and stress protection. In bacteria, carnitine can accumulate at high levels within the cell and acts as an osmolyte by helping cells deal with chilling-induced, salt-induced and osmotic stress. It may also take part in the intracellular transport of essential fatty acids to the membrane in response to chill stress (Angelidis and Smith 2003; Cánovas et al. 2007; Franken et al. 2008).

Sepiapterin and neopterin detected by mass spectrometry in our study were significantly higher in the − 3 °C extracts (*p*-value: < 0.05) than in the 22 °C samples. Pterins are widely conserved biomolecules and described as enzymatic cofactors in eukaryotic systems, are responsible for pigmentation, and have been implicated in nitric oxide metabolism (Feirer and Fuqua 2017). Furthermore, it has been reported that pterins modulate oxidative stress by scavenging free radicals (Oettl and Reibnegger 2002).

The analysis of genome sequences and metabolites identified by mass spectrometry indicates a wide range of cold survival, virulence, and terpene resistance strategies for this fungus. Phain appears to contain large numbers of proteins associated with secondary metabolites, and thus, new compounds with biological or innovation potential could be found with further study.

### Supplementary Information

Below is the link to the electronic supplementary material.

Supplementary file1 (DOCX 349 KB)

## Data Availability

The names *Phacidium infestans* Karsten and *Gremmenia infestans* Karsten (renamed by Crous et al. 2014) are considered synonyms for the same species. For publication purposes, we have chosen to use *Phacidium infestans,* as provided by the DSMZ culture collection. The genome sequence has been deposited in the NCBI repository as *Gremmenia infestans* (name recognized by the International Nucleotide Sequence Database Collaboration). BioProject PRJNA695287, BioSample SAMN17610551, and accession JAFEVB000000000 on the NCBI databank. The protein sequences used in this study are available from the supplementary file (SI-2) provided with this manuscript. The metabolomic data generated by LC–MS/MS analysis have been submitted to EMBL-EBI MetaboLights database (Haug et al. 2020) with the study identifier MTBLS8531.
