# Supplementary material for: Whole-genome sequence and mass spectrometry study of the snow blight fungus Phacidium infestans (Karsten) DSM 5139 growing at freezing temperatures
Source: Mol Genet Genomics. 2023 Oct 10;298(6):1449–66. doi: 10.1007/s00438-023-02073-7 (PMC10657286; doi:10.1007/s00438-023-02073-7)
Supplement: Supplementary file 1 — Supplementary file1 (DOCX 349 KB) [file 438_2023_2073_MOESM1_ESM.docx]

**­­­­­­­Whole genome sequence and mass spectrometry study of the snow blight fungus *Phacidium infestans* (Karsten) DSM 5139 growing at freezing temperatures**

Zerouki, C*., Chakraborty, K., Kuittinen, S., Pappinen, A. and Turunen, O.

University of Eastern Finland, School of Forest Sciences, FI 80101 Joensuu, Finland

* Corresponding author: University of Eastern Finland, School of Forest Sciences, Yliopistokatu 7, FI-80101 Joensuu, Finland; [chahira.zerouki@uef.fi](mailto:chahira.zerouki@uef.fi), ORCID: 0000-0002-3285-5818

**Supplementary information**

**Supplementary information SI-1**

| Table S1. Total scaffolds and contigs obtained in *Phacidium infestans* genome. | | | | | |
| --- | --- | --- | --- | --- | --- |
| Minimum scaffold length | Number of scaffolds | Number of contigs | Total scaffold length | Total contig length | Scaffold contig coverage |
| All | 44 | 44 | 36,805,277 | 36,805,277 | 100.00% |
| 500 B | 44 | 44 | 36,805,277 | 36,805,277 | 100.00% |
| 1 KB | 39 | 39 | 36,801,838 | 36,801,838 | 100.00% |
| 2.5 KB | 36 | 36 | 36,796,597 | 36,796,597 | 100.00% |
| 5 KB | 32 | 32 | 36,780,633 | 36,780,633 | 100.00% |
| 10 KB | 32 | 32 | 36,780,633 | 36,780,633 | 100.00% |
| 25 KB | 31 | 31 | 36,764,729 | 36,764,729 | 100.00% |
| 50 KB | 29 | 29 | 36,688,875 | 36,688,875 | 100.00% |
| 100 KB | 29 | 29 | 36,688,875 | 36,688,875 | 100.00% |
| 250 KB | 28 | 28 | 36,528,401 | 36,528,401 | 100.00% |
| 500 KB | 25 | 25 | 35,229,651 | 35,229,651 | 100.00% |
| 1 MB | 15 | 15 | 28,057,600 | 28,057,600 | 100.00% |
| 2.5 MB | 1 | 1 | 4,329,059 | 4,329,059 | 100.00% |

| Table S2. Selected protein clusters identified by BlastKOALA and KofamKOALA between *Phacidium infestans*, *Lachnellula* genomes, *Trichoderma reesei* QM6a (Trire) and *Saccharomyces cerevisiae* (Sacce). | | | | | | |
| --- | --- | --- | --- | --- | --- | --- |
| Strains | Phain | Lacsu | Lachy | Lacwi | Trire | Sacce |
| Number of proteins | 11381 | 11875 | 9157 | 8978 | 9111 | 6002 |
| BlastKOALA |  |  |  |  |  |  |
| Proteins with annotation | 3847 | 4156 | 3938 | 3778 | 3760 | 3632 |
| Protein families: genetic information processing | 811 | 878 | 846 | 790 | 837 | 944 |
| Genetic information processing^a^ | 687 | 760 | 736 | 692 | 755 | 822 |
| Metabolic pathways | 718 | 759 | 728 | 700 | 723 | 567 |
| Biosynthesis of secondary metabolites | 288 | 296 | 292 | 280 | 282 | 248 |
| Carbohydrate metabolism | 374 | 372 | 353 | 350 | 317 | 239 |
| Cellular processes | 247 | 260 | 250 | 250 | 225 | 287 |
| Signaling and cellular processes | 251 | 284 | 265 | 248 | 220 | 179 |
| Amino acid metabolism^b^ | 224 | 242 | 222 | 203 | 186 | 116 |
| Lipid metabolism | 158 | 170 | 160 | 161 | 144 | 100 |
| Environmental information processing | 168 | 172 | 164 | 152 | 175 | 201 |
| Metabolism of cofactors and vitamins | 107 | 113 | 103 | 99 | 106 | 103 |
| Energy metabolism | 117 | 139 | 123 | 120 | 125 | 97 |
| Glycan biosynthesis and metabolism | 73 | 77 | 74 | 73 | 81 | 79 |
| Nucleotide metabolism | 75 | 76 | 72 | 69 | 65 | 64 |
| All enzymes | 1381 | 1439 | 1390 | 1349 | 1390 | 1143 |
| KofamKOALA |  |  |  |  |  |  |
| Proteins with annotation | 4194 | 4393 | 4134 | 3755 | 3975 | 3617 |
| Metabolic pathways | 784 | 814 | 773 | 707 | 773 | 585 |
| Biosynthesis of secondary metabolites | 315 | 321 | 307 | 279 | 306 | 263 |
| Microbial metabolism in diverse environments | 175 | 178 | 172 | 153 | 165 | 108 |
| Carbon metabolism | 87 | 89 | 86 | 81 | 84 | 68 |
| Starch and sucrose metabolism | 25 | 25 | 25 | 24 | 26 | 17 |
| Fatty acid metabolism | 23 | 23 | 23 | 19 | 23 | 16 |
| Biosynthesis of amino acids | 100 | 102 | 98 | 90 | 102 | 94 |
| DNA replication proteins | 79 | 79 | 76 | 72 | 79 | 83 |
| Chromosome and associated proteins | 277 | 282 | 270 | 249 | 274 | 283 |
| DNA repair and recombination proteins | 164 | 167 | 159 | 145 | 156 | 150 |
| Transcription factors and machinery | 185 | 193 | 184 | 168 | 189 | 248 |
| Messenger RNA biogenesis | 165 | 161 | 156 | 150 | 159 | 167 |
| Spliceosome | 133 | 137 | 136 | 124 | 138 | 86 |
| Ribosome | 124 | 131 | 129 | 124 | 132 | 135 |
| Ribosome biogenesis | 201 | 203 | 197 | 181 | 198 | 214 |
| Transfer RNA biogenesis | 112 | 116 | 112 | 105 | 113 | 123 |
| Translation factors | 64 | 64 | 62 | 59 | 65 | 57 |
| Translation factors | 72 | 72 | 63 | 65 | 68 | 50 |
| Membrane trafficking | 331 | 344 | 338 | 306 | 337 | 322 |
| Ubiquitin system | 135 | 141 | 137 | 123 | 138 | 113 |
| Proteasome | 48 | 48 | 49 | 44 | 46 | 53 |
| Cytoskeleton proteins | 61 | 61 | 60 | 52 | 62 | 52 |
| Transporters | 142 | 149 | 146 | 141 | 154 | 133 |
| Mitochondrial biogenesis | 188 | 202 | 193 | 177 | 191 | 218 |
| Methane metabolism | 21 | 21 | 20 | 20 | 18 | 14 |
| Nitrogen metabolism | 16 | 15 | 12 | 9 | 12 | 8 |
| Sulfur metabolism | 15 | 14 | 14 | 14 | 15 | 9 |
| Steroid biosynthesis | 18 | 18 | 17 | 18 | 18 | 16 |
| Terpenoid backbone biosynthesis | 18 | 18 | 18 | 18 | 18 | 16 |
| Sesquiterpenoid and triterpenoid biosynthesis | 4 | 3 | 3 | 2 | 2 | 2 |
| Phenylpropanoid biosynthesis | 5 | 5 | 6 | 6 | 4 | 2 |
| Isoquinoline alkaloid biosynthesis | 6 | 6 | 5 | 6 | 5 | 1 |
| Tropane, piperidine and pyridine alkaloid biosynthesis | 5 | 5 | 4 | 5 | 5 | 3 |
| Betalain biosynthesis | 3 | 3 | 3 | 3 | 2 | - |
| Streptomycin biosynthesis | 7 | 7 | 7 | 6 | 6 | 4 |
| Phenazine biosynthesis | 4 | 4 | 4 | 3 | 3 | 3 |
| All enzymes | 1466 | 1510 | 1439 | 1335 | 1455 | 1163 |

**^a^**Contains unclassified proteins of the same group; **^b^** also Contains metabolism of other amino acids.

| Table S3. Large potential polyketide synthase PKS proteins identified in Phain. | | | | |
| --- | --- | --- | --- | --- |
| Protein | Length amino acid | pI | Synthesis Protein* | ID% |
| Phain_OT5_Proseq6436 | 2469 | 5.89 | PKS19; Fujikurins, *Fusarium fujikuroi*, S0EET5 | 63 |
| Phain_OT5_Proseq9569 | 2544 | 5.45 | HRPKS; Phomenoic acid  *Leptosphaeria maculans*, E5AE40  HRPKS; ACR-toxin | 52  50 |
| Phain_OT5_Proseq2030 | 2435 | 2435 | HRPKS; Trichoxide, *Trichoderma virens*, G9N4B2 | 55 |
| Phain_OT5_Proseq4400 | 2584 | 5.91 | NRPKS; Citrinin, *Monascus ruber*, A0A161CEU9  NRPKS; Benzaldehyde derivative *Aspergillus nidulans*, Q5AUX7 | 49  46 |
| Phain_OT5_Proseq8153 | 2549 | 6.07 | HRPKS; Squalestatin S1, *Phoma* sp., Q86ZD9  HRPKS; Azaphilon *Aspergillus niger*, G3XMD1 | 46  46 |
| 5388 | 2393 | 5.66 | HRPKS; Brefeldin A, *Penicillium brefeldianum*, A0A068ABB7 | 47 |
| Phain_OT5_Proseq054 | 2486 | 5.68 | HRPKS; Fumonisin, *Fusarium verticillioides*, W7LKX1  HRPKS; Squalestatin S1, *Phoma* sp. MF5453 | 43  41 |
| Phain_OT5_Proseq10592 | 2571 | 5.79 | PKS-NRPS; Cytochalasin, *Aspergillus clavatus*, A1CLY8,  PKS-NRPS; Pyrrolocin, *Fungal* sp. NRRL 50135 | 43  43 |
| Phain_OT5_Proseq5677 | 2576 | 5.66 | Solanapyrone, *Alternaria solani*, D7UQ44 | 37 |
| Phain_OT5_Proseq753 | 3125 | 5.95 | HRPKS; AF-toxin, *Alternaria alternata*, Q50LG3.  HRPKS; Squalestatin S1, *Phoma* sp., A0A3G1DJH7.  HRPKS; Azaphilone, *Aspergillus niger*, G3XMD1. | 34  35  35 |
| Phain_OT5_Proseq4534 | 2582 | 5.74 | HRPKS; Alternapyrone, *Alternaria solani*, Q5KTM9.  NRPKS; Azaphilone, *Aspergillus niger*, G3XMD1. | 34  35 |
| Phain_OT5_Proseq10228 | 2397 | 5.79 | NRPKS; Usnic acid, *Cladonia uncialis*, A0A0R8YWJ7.  NRPKS; Anditomin, *Aspergillus stellatus*, A0A097ZPE0. | 36  37 |
| Phain_OT5_Proseq11171 | 2596 | 6.75 | HRPKS; Alternapyrone, *Alternaria solani*, Q5KTM9 | 33 |
| Phain_OT5_Proseq984 | 2583 | 5.39 | NRPKS; Ascofuranone/ascochlorin, *Acremonium egyptiacum*, A0A455R5P9 | 41 |
| Phain_OT5_Proseq9515 | 2623 | 6.01 | HRPKS; Alternapyrone, *Alternaria solani*, Q5KTM9 | 33 |
| Phain_OT5_Proseq6910 | 2328 | 5.30 | Red-PKS; T-toxin, *Bipolaris maydis*, N4WHE3 | 42 |
| Phain_OT5_Proseq11252 | 2265 | 5.65 | Red-PKS; T-toxin, *Bipolaris maydis*, N4WHE3 | 42 |

*HRPKS, highly reducing PKS; NRPKS, nonreducing PKS; Red, reducing; NRPS, nonribosomal peptide synthetase.

| Table S4. Partial pathway of pyriculol found in *P. infestans* using Geneious Blast. | | |
| --- | --- | --- |
| Gene | Protein function | Full-length, identity with *P. oryzae* proteins |
| *PKS19* | Highly reducing polyketide synthase 19 | 39% |
| *RED1* | Short-chain dehydrogenase RED1 | 58% |
| *RED2* | Short-chain dehydrogenase RED2 | (two proteins; 42%, 59%) |
| *RED3* | Short-chain dehydrogenase RED3 | 48% |
| *OXR1* | FAD-linked oxidoreductase OXR1 | 50% |
| *OXR2* | FAD-linked oxidoreductase OXR2 | 45% |
| *TRF1* | Pyriculol/pyriculariol biosynthesis cluster transcription factor 1 | 66% |
| *ABC7* | Transporter | 63% |
| *MFS1* | MFS-type transporter 1 | 38%, slightly shortened |

| Table S5. Compound details generated by mass spectrometry analysis. | | | | | | | |
| --- | --- | --- | --- | --- | --- | --- | --- |
| Compound | Annotation level | DP**^a^** | Relative precision**^b^** (%) | LOD**^c^** | Retention Time[min] | Monoisotopic mass | Formula |
| Carbidopa | 2b | 20.7 | 7 | 28080 | 5.33 | 226.0954 | C10 H14 N2 O4 |
| Sepiapterin | 2b | 60.3 | 3 | 4478 | 4.83 | 237.0862 | C9 H11 N5 O3 |
| Neopterin | 2b | 46.5 | 3 | 5198 | 4.29 | 253.0811 | C9 H11 N5 O4 |
| Betaine | 1 | 6.9 | 8 | 24207584 | 1.27 | 117.0789 | C5 H11 N O2 |
| Carnitine | 1 | 3.8 | 17 | 554572 | 1.50 | 161.1051 | C7 H15 N O3 |
| Hexose | 1 | 25.0 | 6 | 134594 | 1.42 | 342.1163 | C12 H22 O11 |
| Sugar alcohol | 1 | 31.3 | 6 | 37693 | 1.25 | 152.0684 | C5 H12 O5 |

**^a^**DP is short for Descriptive Power, which is calculated as the ratio between the standard deviation within experimental samples and the standard deviation within the QC samples. Variables with a ratio higher than 2.5 are most likely to describe variation related to the experimental design. This smaller set of variables are in the remaining part of the report referred to as the reduced dataset.

**^b^** Relative precision (%) denotes the relative standard deviation between different injections of each QC sample.

**^c^**LOD is the Limit of Detection (LOD) indicates the lowest value of a compound that can be differentiated from noise.


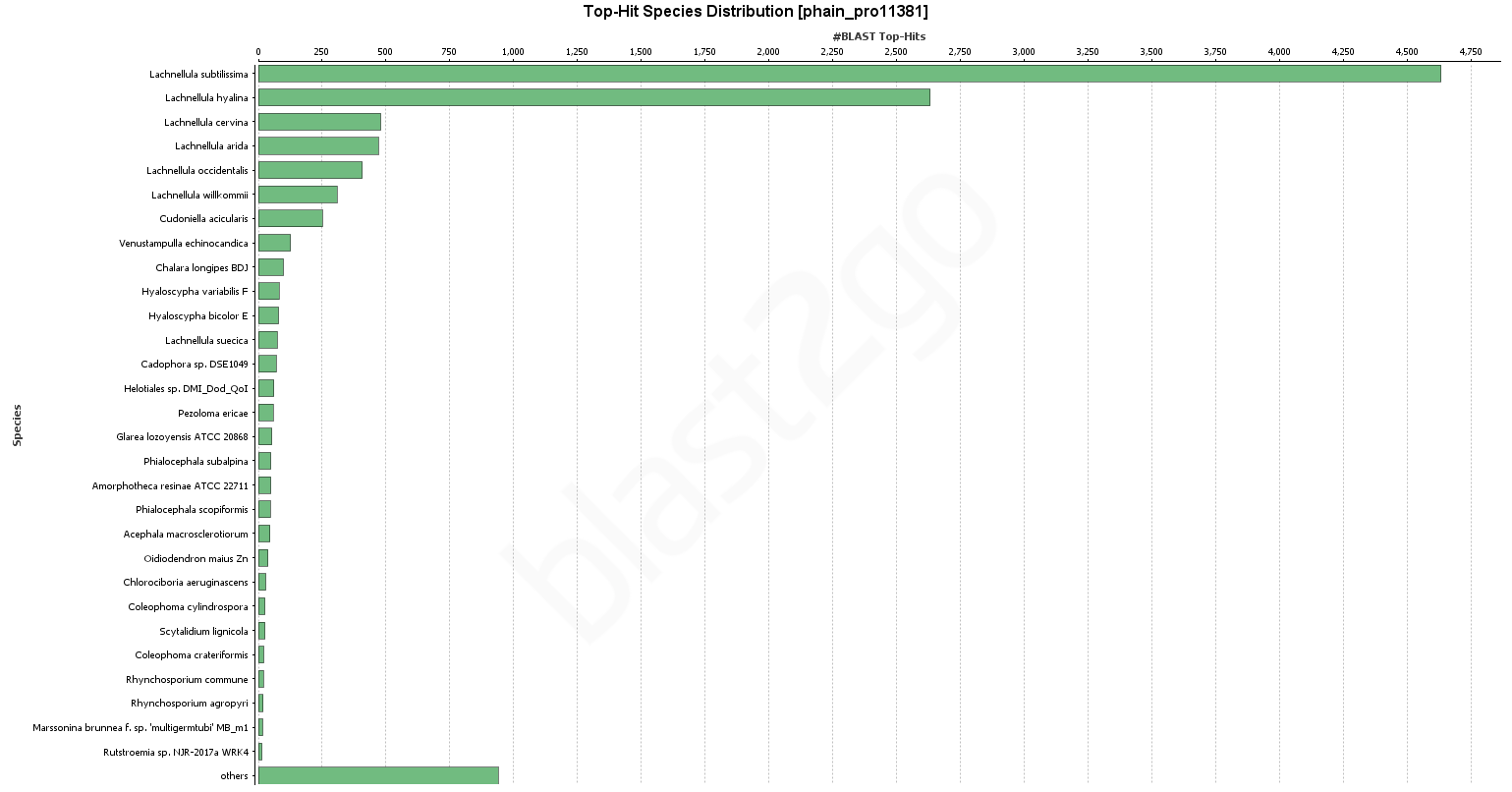


Figure S1. Species distribution of Phain using Blast2GO.


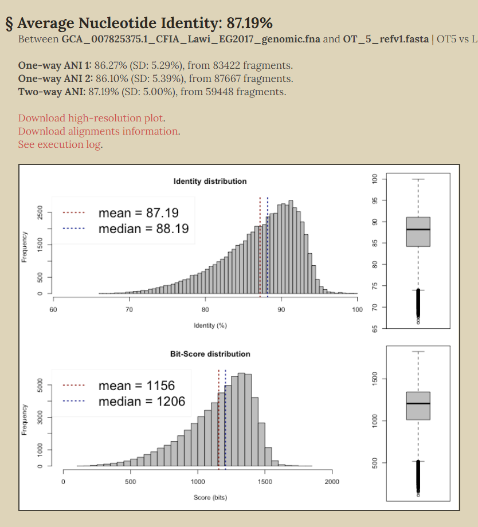

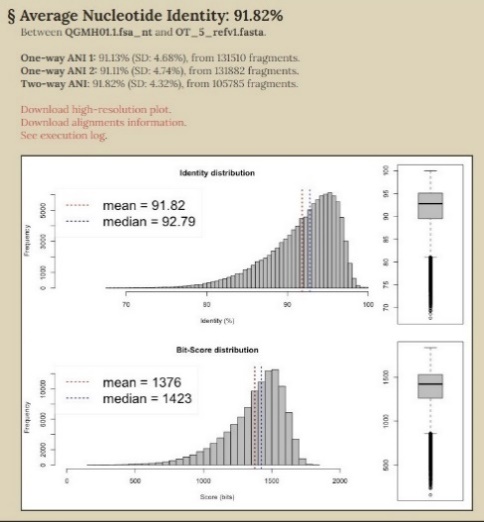

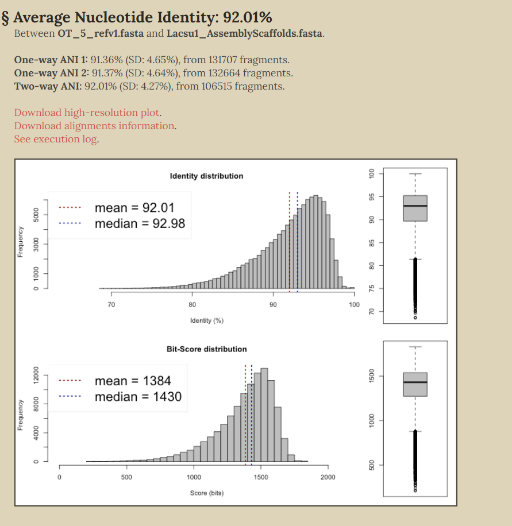


Figure S2. Average Nucleotide Analysis of Phain with the closest reference genomes. A) Phain vs Lacwi GCA_007825375.1, B) phain vs Lachy CBS:185.66, C) Phain vs Lascu CBS:196.66.

**Supplementary information SI-2**

SI-2 supplementary information file includes the protein sequences extensively studied in the manuscript. The annotation was generated by Augustus <https://github.com/Gaius-Augustus/Augustus>).

>Phain_OT5Proseq471

MKSISSLVAFGGLLLSASHVAAYGASSPGPVVYKGCYSTSTGLTMNSSYTFNTQGYCQTQCAPLSKSVLATKSSTDCWCGNELPPTADLVSDSHCDAPCAGYGSDMCGSSQDYWSVWLSGVASSASTATADDTSTTSSAAQSSTATSATSTIPAVVTAPGSTVVVTAAPGTSESSTASASATPKKGTNKAAIAAGVVVGVVVISAIAGGIYLFIRNKKRREVEEEYRRNAAVSTFIAGGGKPPNSSGGGSSFTDTRLDQATMAQRRMSDGSIADNEDYSRRILKV

>Phain_OT5_Prose5443

MVKAVVQIRGDSKITGTVTFEQDTESSPTTISWNITGHDANAERGMHVHQFGDNTNGCTSAGPHFNPHGKTHGAPSDEVRHVGDLGNFKTDAQGNGVGSVQDKLIKLIGPESVIGRTVVVHQGTDDLGKGGNEESKKTGNAGGRPACGVIGIAA

>Phain_OT5_Proseq10124

MPASTTVTTAGPGNGNVKLRSAMGKEEVKEKEEREEEKLGWEKHEKDFYWTYTEEPHRTRRMAIIKAHPEVTKLCGPEPLTKYLVLSVVLLQTTTAYLLRNTPFWSWTMWGTAYVIGATANQNLFLAIHEISHNLAFRSARANRVLAVVANLGIGVPYSASFRPYHLTHHKSLGVDGLDTDLPTSLEALFLDSILGKAFFCTFQILFYAVRPIFIYQVPFTSIHVFNIATQLFYDYLLVHFSSWNALWYLILSSFLAGSLHPCAGHFIAEHYLFDAPTASSTASSTSSSTKDGNTVQTDLPETFSYYGILNLFTYNVGLHNEHHDFPAVPWSRLWELNRVANEFYVDLPGHKSWVNVIWRFIGDKGVGMRCRVKRREGGRLVGGKGGAWKDAELEVL

>Phain_OT5_Proseq10228

MSIAPPPTVNLGDLHLPRILCLHGGGVNAEAFRLQCRGLFRSLNGTFRLVFPDAPYLSPADPGVMPTYAHLQPFRRWLRWEIEQANPGPEAICGDIDRALHEAMINDNAMGATGDWAAIMGFSQGAKLAASLLLRQQLRAEKLGKDQAGSDFKFAILLAGRAPLVALDADLSDSLALADADELTTGAFAQVTGSFLQGSDHVLRLPTLHVHGKKDPGLANHRRLLTDFCKAGTTRLVEWDGDHRVVIQAADVAAVTKEILALSKQTGVISLQVFLITTSAMELFLCGPQASLPSPKDLGQLRQILLGYNSLHASLTLTLKELPNLLRQLTAFDVSLKRASSAASIDFLLQWLDSGHLSLPVDDLPNIISLPFTVILQIALFLQHLAKNNTGSDYSQMVPELPRHGVQGFCIGFLTAAAINFSGNEEQLAEYTATSLSLAMCIGAYIDQNALYAEPPNPVCALSVRWKEGKVSRCQVENLLAGYAHAYISCITDISCVTITAQASDEKQLTEVLREAGLRAKKISINGRFHSAADHTTAAQTLKRFTKSVKGLQYPEVFQLQAPLRRNDTGGVISGGDSVTDIAIESILLHTADWYTTLRKTFDVFPTDYARVTTFAFEEGVVPLSLSQRNEANYHVNVTNGANGVNGVNGINENLEAKYPPHSVAVVGMSCRFPGADDVDKFWDLLESGVSMVREVPSDRLNLNNHRLADYAKTKFWGNFIDDPESFDHRFFKKSAREAVSWDPEKRILLEVVYEALESAGHFGPAAADHPNDYGCYIGAVGNNYYDNVACHPPNAYSMLGTSRAFFSGRISHQFGFTGPAMTIDTACSSSLVAINAACRAIQSGECSRAVAGGTNVFTSPFDYQNLAAAGFLSSTGACKPFDASADGYCRGEGVAAVVLKPLSAAIEEGDHVLGVIVGSAVNQNHNDAHITVPCSSSQTAVYNKVLGMANSSSSSVTYVEAHGTGTQVGDPIECQSIRDAFGGSHRKEILHFGSVKGHIGHTEASAGVAGLIKILLMMQHNTILGQASYSNLNPKIPALEPDMMAIPRQSQPWKTSSKFACINSYGAAGSNAVVAIREAKPFSSTPRTKRPTITTKQPFFMSASSEASLIAYARKLLAYVEVQRTFSENTSHLLSDILFNLTDRSNHKLAYSVSKTLIYLEDLEKMLSSIVAGSETPIKDAGSGPRPVVLVFGGQEKDFVGLSKEVVDNSALFRSHLDTCDFELQSLGLGSLYPAIYQRESIENLPTLHAVLFAVQYASAKAWIDSGLRVSSIVGHSFGQLTALCISGCLSTQDALKLVVGRAELIHSSWGEERGSMISVQSDYAVVNSILETINAESPDKLEIACFNHPTNHVVVGTTKAVDIFESHIAGDTRLQQNIRVKRLNVTHGFHSALTEGILPGLESLANSVQWNKPTISIELATEEHIDQELGAWLIPHHIRNPVYFSSAVQRVAQKFPSCVWVEAGQGSSVMSLVKNSVKGEGQPLYCPSFLNGPNAISSVAETVVELWKTGIPVQFWPYHRSERSHFEYKSLPPYQFEKTKHWLPFINQAAPEPAPTMAPATQTIVTHEFISFLEFSDSSKEAVFLVDPESERYMYLLNGHIASNQALAPASLYVELLSRAAMILTSNASFDTHVINMNSMQMKGAPIGLDSKKNIYIKLTRITPDVDYWDFEFSSKSKEGGEVQVHVIGKVGLGKRDDPALADTIMQWSALIGYKKCLSIMNNEDGEKMQGRHIYQALQKLIFFDEMYHGIKSISYQGHEAAGKVAATLDPKLSPKEALYDTPTIDGMMQFAGVLVNYFAHPSGKDVLLCQGINRIVTGGAFDITAGEWTAYSLLTEDTDERTVSDVYIFDKKSQQVVIAFIGFVFTRTSVSVLQRSLRSVNSGGSGANPTPAPAGVAPIPVAVTGVSAESSKASKVLEVFHNVTDIPMDEIALSSTLEELGVDSLLVTEVLNEIQTAFGLEIDLNTFLFFPNVKAVCDYIDSALGLGIRAEPPALAEAKVKDVAVRTLPTNSRDVESRPSLQRAQKVFADCKDAYERAAVETKAVEFWEKCYPRQAALVLAYVVEAFAKLGCDMASIKAGNAAPMIPHLPNHKQLVRQLYRVLEDAGLIIVNDQGQFVRTTKLVDPTPAATIFTQIIPEFPLHASVHRIVQVVGSELAECLTGEKDGLQIVFGNKENKKTLDDLYENWPLVRSGTIALGEFLEKAMANADKPGVFRILEVGAGTGGTTKYIIRHLQKLGISFEYVFTDLSPSLVAAAKRTFKDCPEMEFATLDIEKEPPASWISSFHFIISTNCVHATRNLTTSLTSLRKLLRKDGVLTLVEITQNMFWLDIAVGLFEGWWLFEDGREHAVTPEWLWKEHILRAGFNAVDWTDGEEPEARTIRVIAGFPTSSST

>Phain_OT5_Proseq10592

MPPQKLEPIAIIGTGCRFPGGANSPSKLWDLLREPRDLATKIPLDRFNINAFYHPDGSHFGTTNVKESYFLEEDPRHFDSQFFNIQPAEAEALDPQQRLLLETVFEAIESAGVEIESLKGSNTAVYVGLMCDDYSEIVRRDLNSLPTYAVIGNSRAIMSNRISYFFDWRGPSMTIDTACSSSMVAIHQAVQTLRNNESHTAIAAGANLIFGPEAYIGESNLHMLSSFGRSRMWDAAADGYARGEATGALVLKRLCDAIEDGDHVECIIRETGVNQDGRTQGITMPSETSQAALIRDTYMRAGLDLTEKKDRCQYFEAHGTGTAVGDPKEAEAVATAFFGDENNIIPDDMLFVGSIKTVVGHTEGTAGIAGVIKASLALQYGVIPPNLHFNQLNPAIEPFYKHLQVPTAVAPWPFVPQGQPRRASVNSFGNGKKTLHLISTLIQHQGLGGTNAHAILESFKSLEKNQCSIKKPKVKSSFALLPFVFSAASERSLVATIRNVSNYIKANESIHLGNLAFTLSQRSVFPFKAAFSASTGPELISKMDTKLALFDGNPGIPCAIRFSSSASGILGVFTGQGAQWATMGRELLRSSEAFQETIVELEASLARLPEKDRPSWSLQAELMSEASFSRINEAALSQPLCTAIQIALVDLLRIAGIRFDAVVGHSSGEIAAAYAAGFISAYDAIRIAYYRGLLAKYACGPDGKSGAMIAIGVTYQEATELCSLPQFEGRVKLAASNSSSSITLSGDVDAIEEIKVRFDGEKKFSRILKIDTAYHSHHMLKCSDMYMQSLRACNIQVQTPPADACSWYSSVYGGMKMISNHLLKDTYWRENMVGSVLFSQAVQCAIQTQNSLFMALEVGPHQALKAPVQHTFQEIPGSESFYASTLSRGQNDAEVFTEMLGFLWSHLKPSAVNLRAYGKSIRDNDGALKLMKGLPSYEWDHERVYWNESRRSMLLRTRGDPRHELLGCRLPESIDGEIRWVNFFSPNEIPFVNGHVIQGQKVFPAAGYVTMAIEAAMIMVRGKSIKLLEIEDLKVLRALGFDDDSTGIETMFILSSITSRNGAEEEITGNFACYSCLNKQAGNMVMMASGTVQAILGACSDISLPNRLPEDKTLTPVYSEPLYASLAELGYNYSGDFKSLSSMRRSMNKAVGLITNPLDSESASSLLVHPAVLDAMFQTMFVAYSSPYDGRLWSLHLPTGVRRITIVPTRCVASMGSKVLFDAESVGSDCNTLSGDVDIFTEDSTKPIMKVEGMAFKPFSEATATDDSELFSEAIWGVASPDGSAVTAEDQATEEDIELGQISERVAYFYYRNLVETVSVAEREKLNMPFHHRSLFNAASEIISQVSEGRHPFARAEWNLDSKGALIEKMSKYPESADFNIMRAVGENLPGVVRGETTILEHMTKDGLLDDYYKRGLGFDLSHDLLSRMVAQIAHRYPRMNILEIGAGTGGSTKKILPRLDRAFSSYTFTDISIGFFEKAQDIFKDYSSRMIFKTLDIEADSVPQGFEDHSYDLVICSNVLHATKSLRQTMANVRRLLKPGGYLVLLEVTNNEPMRIGFVMSGLPGWWAGADDGRTFSPTISLDRWNTLLQNSGFSGVDSATPANNPLPYPASVFATQAVDDRITHIRQPLLSTGIEKSIDYLLILGGKTPQTARLAEDLTWSLRKYCKHVTRVEGLDKLHDTDMFPMTTAISLTELDEPIFKTMTEEKWEALKLLFDLSRNVLWVTRGCLDNEPYSNMIVGLCRSVGYELPHLQIQLLDIESSEMPKADLLSEMLLRLQMKDYWEKKEQLHDIVWSTEPEYILRQGKLHVLRMMPSKDRNNRYNSSRRFVTKEADTQSLIRLENQEGAYFLTESRKTLPPVPLNSSPTLAMHRIEMSYSLVSPIRLAPQASLFLCLGSTKMNGVQTKVIFLSDATTSLVDVPENYSIPYSPPTDQEVHSLLTANSHLLSLSILGNVPSGSAVVLNDPEPYLVDALKTQATKKGISLTLIALDGYERDLPWVSINRRSSKRMIKLALPKGPSIFIDCASTDYAREIGSRIRQCLPKPCEERMPKFYLGDQASFLGFLKDAVAFSQSALPNPGNSSTAQCVPLREVANILNLPQLTTVIDWTSPASVSVKIQPADSANLFFQTKTYLLVGLTRDLGQSLCQWMLTKGAKYVVIASRNPQINPKWLEGLASQGAIVKVMDITNRNSVSKIYDDICKTMPPVGGVANGAMVLCDTSFPRMSYDELQTVLRPKVEGTQYLDEIFSTHCLDFFILFSSLMAVLGNSGQSNYSAANMYMTSLAAQRRKRGLAASVMNISGIFGIGYVSRVDDVTQKQLAKIKFKPMSEQDFHQMFAEAVLGGTPDSGVNPEFTAGLQQVSDDQNASWGKNPKLSHFIRSPEEVTTKRDNREVVASLQSQLLLAATEEEATALIEGNFRRILYSAYANKVVASLSTRIQRSLLLDDDPDIKIPLIDLGVDSLVAVEVRSWFMKELNVDMPVLKVLGGASISDLAAMALEKLQLEFVLNIGTPEGVGRKSAPMRSTSRTLSIRSDRSRSRGSSSSNTSLPTTEASTPPFSDKGLAPKFD

>Phain_OT5_Proseq10963

MYHTLKFTYLAVLATQIGVSLSHPSLEHIEKRFVPLSMGLASSWGAIAHTTLTSTGNTLITGNCGTCPGTAITGFPPGVCTGTTSAGGTAACLAEDACLSAYNNARAAGPTVALPAADLGGMTLPPGVYTFPTSAGSLTGAVTLDGATNATGQFIFLLATTFEAAAASQILLLNGAQACNVYIIVGSSATIGAASALQANILAYTSVRTSFVVSNHVTD

>Phain_OT5_Proseq11171

MPEKLPALRPMNGDDKDTREAIAIIGLSFKFPQEATSAQYFWEMLMQGRTASTETPKSRMHTGAFRLSGGRQFDKFTTSKACFIKDDIAAFDAPFFSISREEAETMDPQQRALLETTYRAFENSGLPMEKVSGSKTSVFVGNFTDDYKMMYCKDPEQPFQYGATGCLMSVLSSRLSWFFNLTGQSLSLDSACSSSLTAVDIACQNLLGKESDMSIIGASNLIFSPDMMLLLSSFNVLSPQGRSFVFDERANGYTRGEGHAVLILKRFSDALNDGDCIRAVIRSSGSNQDGQTPNGMAQPSRKAQESLIRHTYEKAGLTLSRTMFVEAHGTGTRLGDAVEVNAIGNSFKSSRGEGESLYIGAVKSSIGHLEGCSGLAGLIKTVLVLERGIIPPNSGFQKANIRIDEEFLRIKFPLAPTLWPTSDLRRASVNSFGIGGSNCHVVLDDAYHYMHLRGLTGRHNTTRHCPNQINGNLKSQKNGDAKAQDSFPLLLVWSSADENGINRWGKLYKDFFSECTAQRSLWDQSYYRNLAFTLACRRSMLLWKSYAIISPTSRLQLSDTIMSKPRLTPSTCPKLGFIFTGQGAQWPGMALELMGYPVFRKSLQDAETYLGSLQCCWSIKDELSKTEDYSNIHLPEYSQPICTAIQVAIVDLLRSFRIFPSVVVGHSSGEIAAAYCAGSISQEAALKIAYFRGLYTKLLSESSHVQGSMLAVAISEPRAAALIDEVAKTFGKLGLWIACINSKTNITISGDRDQIEALELLLDEIGQPKQRLKISVAYHSPHMVPVIELYKESITNIDTPKETISCPPIMLSSVTGQVLSPEKLNSSDYWIQNLLSPVRFGDAVSPVCVGSRNEPRASLDRSHRRTTLVDFLIEVGPHSALRGPVRELLEECSNQGRTTYLSAMTRGVSAAETLLSVAGQLHCSGYTMNFTAINNPDKSMQGIQILTDLPEYPFDHAKSYWLESRLSKNFRFATHTNNVFLGKPVSDWNPLDARWRNFISISAFPWVMDHKIQNQYLYPAAGMLTMALEAVRQTAGSEDNVRGYLIKEASFHTPLIIPAKGSIEVQISLRPENSKRSRENQINWFNFRIYSFQDSNSIEHCFGNVGVEYSRKANGVVKDRKDEDDFLHYQSIQTSTEKVSCHQISPEILYNSLKSAGYNYGPQFRVLETIIHGPRTCSATITISNDRSCNLHTIHPATLDAFMQVLLVSCIGKDLCVSSLKIPTHIKSLWISNRISKVPASSQETNILRASADLTLESVSSCQGNVYVLDTKQGSVFAKLEGLEATLAASTRLSIGGIPPKDPLTNMCHRITWMPDFTLLSNKELFQYCKEMQTIKPDLVQQFEQLDCFLYLAIHRTSQLIKDIHTFPNKTVSRYVDWICQQDRLYEASSMPNFRLEWKLLRENDEYFSDICLIMQKTSNLGHLCLRTWQSMPQLLRGEVDPLSLLFQDELLSDTYKELHETSNWLSSLGDIATHLAHLESGMKVLEVGAGTGATSRVLLEALSSQAQSGLRHVQYSQYDFTDISSGFFEQAKKEFQESKRLKFLTLDIEKDPGSQGFVLGTYDLIVASLVLHATRALSVTLKHVRSLLRPGGKLLLLEFVRPELSRFAFSMGLLPGWWLSSEKFRLSGPLIDSSKWSDLLLEAGFSGNDIELKDFESNNCSQMSLILTTATEVSKPEGHTVKQEIVMIAPELETSQLDISQTNAHLEIKGYKVTRTTSLKQALASSNPDAAYLCMIELFQPFLRDMTADQFASLQRLLQAAKTVIWVTRGGGPNAHPDYAMIDGLSRVLRNENSQLSFVVLAFEIAIHLTKTQIEKLGAVLEKALYGTVSKDGENTFIESNGRLQIGRIIPADEFSQPARSRSTKPMMKTMQYSDTGSTSLAMQDIGVLDSFYFRPQTTPETLLPMEVEIEIRAVGLNFLNVLAATGIVTERSFTHECAGVVVRVGKGCQNFQPGDRVCGWGTEVFKSHVRLNYECVAKVPKNSSFPEAACLPFSFLAAYYILDLVRARRGESILIHSAAGGLGQATIQMSRMLGLSNIFVTVSSAEKKSLLIDTYGIPEDHIFFSRDTDFAQSIKRISTTAVDIVVNTLSGEGREASWDCVASYGRFVDLSRTDRSSSSVRMRNSAANVSLFAVDMINLISERREILSVHFKKVIELFEERKLYYIKPLGVYSVENLGEALGHLRSGKSMGKVAVVLDKQASVPKVAIRPTEHFNLAGAKSYLISGGLGGIGQMVARWLVEQGARHLILLSRSGPTGHDTASFLQELEDDGVIVKTPACDVSDAEALQALLEQYLVEMPPIRGCIQASMVLADSLFDNMPFLSWVSSTAPKTNGSWNLHTLLPSDLEFFVSFSSLCGTFGQVGQANYAAGNTFQDALARYRTSLGHRNSYALDLGIVSGIGYLSRNKEITERLMSSGQFLTITPDHLRSLLDTCIRKKRQPSSPTRIVLEESQILVGLQTPHMKQQRSPLFRHLYLYQESSSNPTTFAAPELVGLKSRFLAAAPLEAALVIAHSLRTKVAHLLSTSAASLTNHDIDIFQPLLNYGVDSLLGVELRNWLAREFAADIPIFEIMGGASAIGIGSSIVKLSKLKDNS

>Phain_OT5_Proseq1309

MDSSYAHAQAMGPSPFFYYNPDPKPDNRQHGHFSQQPSNIQVPIYHPHMYQMPSTPIYSRPNSSCSQAPMQPVFSTNYAANMTPLASPRPMYHKPTILIQEHAPHLMMESEANDMYYYPSTPPLSASGSAISSPTSCDVLPTPLNTSFCGLEGFEGVKEGCEGEVQSENLAGNEWTRCGSPPMTPVFIHPNSLMTFGSANDLQPATSCPSLASPSPSPYPRSVISEQDFDFCDPRNLTVTSGTSVSSSNTAALSSNPILAKTAAEFPPLPTLCAGDDEEHRYMLGGETYNNQVPETQDTNQNLNLSFTAPSHHGLPTFDHLSDLDSEEDFVNGLVNFPATDNVQFLGSKRQRTNSGSDLVSIDNGPFISEDEFEDFEEFEDSEQFAVACLPSPPASGSETEVKKEKKEKRSKRSKKACRDDDEDSTEFDNLVRSRKYTVPMDSASGAPAQESTAEGQQNNSAPSQSGSSEQHATNSVMGSESGATPTQAPVNRRGRKQSLTEDPSKTFVCEICNRRFRRQEHLKRHYRSLHTEDKPFECHECGKKFSRSDNLAQHARTHGSGAIVMGVLEEGELPSEHMDSGDGEHIHSLGSVLFNVAAAASGSDSEHSSEGGSSGTDSQSRKKRKRSE

>Phain_OT5_Proseq1467

MGGASYAPQQLYYDGKVQAATSGKIFDSIDPSTAQPLAKVHAASPADIDLAISSAQKAFPSWSLTPPIVRSRILLKAVSLLRSRNDEIAKVETHDTGKPFSETSTVDVVTGADVLEFFANLVASGGLSGETTQLRPDAWIYTKKEALGVCAGIGAWNYPIQIALWKSAPCLAAGNCMVYKPSEVTPLHGQILAEIYAEAGVPSGVFNIVHGDGAVGAVLTSHPGISKVSFTGQVSTGQKVAAAAAEGMKYVTVELGGKSPLIILPDTELENAVDGAMMANFFSTGQVCTNGTRVFVHKSMKAAFEKRLLEKIEYIRSLEPMDPNSNFGPLVSELHYKKVLSYIQHGIDTDKAKLLSGGPQKPTKLPNGYGDGFWVQPTVFTDCTDSMKITTEEIFGPVLCILTYDTTAEAVARANNTKMGLAAGVFGKNLNECHDVISKLQAGITWVNTWGESPAEMSVGGWKMSGMGVENGRKGLDAWVREKSTLVEQGGVVGTVFAKL

>Phain_OT5_Proseq1552

MDSYSYNDNSATWIKPRLPSSSSSYGVSKALSARLNPVDNKANSSHLSLLNRLIRFIIRRKIPEISIPWEERYGIPLGEGATYTVEKVRLETSAESLRSKWVATKRAKVVVPKQFTTGLSFNDQAYRRLQAVLLELEILSHPGICHHPNIAPLIGYSWDETASGYAPILVIELAVFGDARSFLSLTELSDEEKLSFCGDVASGLEALHTCDIFHGDIKLENILVYAGTKSRFLAKISDFERSPQIETGDQTICRGTEIYHAPEVQAAHLKNKPLLASNSELRHFDAFSFGLLTLEVLSGVKWYGELSGGNILVKQVLSGTATYGDIVHTSLQILKSSSPVCPALQPIAREILQCTNGFSEGWAPIRNIFRQDRLDPNEEPAASMLIIRPEVGENTHYSILEISSQINISKEWLGDSMWSELEWTATHESRPPRRGQAAFELFVLYGLAVYSPDISIEDALDYVTSAALSGYGPAFIVGKRLFAANDLSIPQVFQEGPKDTRLGQQIMSLESKPNDEYYSSAVQLFWSQHIRNLSHMSISKLISKYDEHSETEFMKWLLQNQSDLDAKEFRVFAAENFLIHQAVGRASCEVVSLLLDLDLDINIQTPDGLTPLHLACRFADPTMIYQLLQNGANPCLLASDNTMPLHWIVLLDEPQVHPVAKALIQASTAIIFPCSSTYYFDELGLLLKGTPLAWACRCRNLCAVKVLLDLCANLLSEKDLEEVCLPFSLAMVCPQITGSLLQYNDYYLSLNPAQKTEVFRNIGMSNSSNFQRWLVHGSYFDSVHGEIIDCLSRYGCNLSLDTTRPRTMVIVDGKIVTGEYSPLTRAALSHNLPLAKEFLRLGVDVNDRDADGYSALEIAISTSEFVGSPDSTIKFVDFLLQHGAATGSAENVPGRCHPGRTESALILACKSFASVRLVKLLALASPSEIDQKYSGDTPLFLVSYGGDDDVLSKIQALVEAGANIQIECNHRDQLMGCCQTPLAASLFALNWPVAKYLLECNSTTDFGTTGAHKQSVLHLLVYLSFTIEARNRPGEIAVLVSVIKKLLQHPIARENNLLNTPNFLGVSPLRLAVFYGLPRLVKIFCDDFYGPCTDLKAIQEDKISLQWYLSDTFRDTFAGNRISPFGIPDGTGGVDMSLEECKIDGDLYPCIEDIAYLRRIKEVRDLLEVKSGVPVILEALTATTPAGTYAKEISDEMRVARESTLAFCMR

>Phain_OT5_Proseq1771

MLPSPRRAFAAHSSFPNSGHRSTFLYHVYELSKPAFTYNTNTNLNKFYISTPPKPTRPESAAPAAIAKMLDENLPTFYIRPSSSDNPLSNTIFLTQNGSEPQAEYTLRRPDPNTPASKNCYAVALYDSYNPDVLYAEVLVQPDWTQPTLSQAEIRSQNGVPPAPVPIVPDNFTIQLYNPDQQVKIRQVAGSWNSSAYWEFDMPQNSFKMPSASALDRSQSDPAALDITPKVAFKWKKDGKLSKDISCYLSGKTVDGRKSKEPDIPVAMYNSGKEQNNLTIYQPNMHRVDVEDAKGLEVVFLLSAAVIKDIFFNPSRELFNISSPKPRTNTNPLRKNSGPVIGGKAASPPVMSGAVSSQPPSASQAPPQQAYRPQQQQQQIPPRQQTSRPPPANPRTQWEIDAETARLKAEFEAEQKEREKIERKEQKMIKKMLEEEEMEAKRREAEVAKETERLRKQYGVPAAASPPNHVSFQQPALPPRQHSSHVRPHSNSLGQPQQFSQYQSAPHSQPVPYQRPVSTPLAPQQQQQPATQGSWFGSNNPSQHSGPYLQAPGNNASASHSGFFGLGGGKKVQKKRSVFF

>Phain_OT5_Proseq1811

MRFSTLATWAGAASLVAATPLNEEKAEKRDTPGFTSGQPIDGNGKGGPILGGTNHQLDLQNPDNLAAQSTDNGLVANLKWSFSDSKSKIFNGGWTREQVVTDLPASHDIAAAQQHLKKGALRELHWHRAEWGYVYSGQVLLSAVDEKGEYQVDKLNVGDIWYFPKGAAHTIQGLADENEYLLVFDDGNFDATGTTFMVDDWVAHTPKSVLAKNFGVNESVFTNVPATDPYIINATVSTAGPTGPSSGLTGNSSYVFHNSGTAASVPVPGGGGNLSIIDSRNFPIAKTIAATVVSLEPGALRELHWHPNAEEWLYFHSGTGRATVFIGGANARTFDFSSGDTAVFPDNSGHYIENTSKTENLVWIEIYKSDRVVDISLTQWLALTPSDIVASTLKIPISVAQNLKKEKQLLVKGN

>Phain_OT5_Proseq1952

MTSTKTSGDQYRSYQDDRSAKSQDTVYTTSNGAPMPHPYESQRAGENGPLLLQDFHLIDLISHFDRERIPERVVHAKGSGAHGVFKATKGLEDLSIADMFTKGKECPVTVRFSTVGGEAGSHDCARDPRGFSVKFRTDEGNWDLVANNTPVFFLRDPAKFPYFIHTQKRDPSTHLTHADDSTMFWDYLSQNPESVHQVMILMGDRGIPDGYRHMHGYYGHTTKLVNKNSEWVYAQFHMISKQGTKFLTQEQAAEKGPDYSQKDLYGAIESGDFPSWTLEVQTMTAKEAEELWEKQRINIFDLTHVWPHKQFPLREIGEFTLNENAVNYFAEIEQAAFNPSHYVPGIEPSADPVLQSRLFSYADTHRYRIGANYQQLPINAPRPNYRMANFQRDGAMAFNNQGARPNYMSSIEPIHFHERKTNLDKVHGQFTSDAITFLSEIRPEDFNAPRALWEKVFDEKAKERFIHNISGHMANCKKEEIIKRQIAIFREVSEDLASRLEKATGVKGYDGISGLTFNGTHNGMAKDSGNKAANGMSRASIRGSGNNGAPTKGDHQVNGANGQNGIAAH

>Phain_OT5_Proseq1964

MSATLFRTSTAARSALRAGAATRAASAASTSFVRGKATLPDLPYDYGALEPTISGKIMELHHKNHHQTYVNSFNTATEQLQAAESKQDIAAQIALQPVINFHGGGHINHTLFWENLAPSKNGGGGEPAGQLKTAIETSYGSLSSFKTKFNAALAGIQGSGWAWLVKDIETGQVQIRTYANQDPVVGKYKPLLGIDAWEHAYYLQYQNRKAEYFSAVWDVVNWKTVEKRL

>Phain_OT5_Proseq2030

MAEKINGTNGTNGTNDTNGFYNSNGVNGVHNSNGINKPNGHNNSNGNGAKSSSEIDVSESSYEEDPICIVGMACRLPGGIRSASDLWEFLSCNKSAQGKVPKERFNINAFYHPDGSRAGVMDADGGYFLQEDVRQFENSFFGINNLEATYMDPQQRKLLEVVFECFESAGVSMDQISGTNTGVYVGNFTVDYLTMQARDPDYMHRYNATGSGTAIMSNRVSHVFNLHGPSFTIDTACSSSIYCLHNAVNAIKNGECDGAIVAAANLITAPEQHLGTMKGGVLSPTSTCHTFDASADGYGRAEAVNAIYLKPLSSALRDGNKIWSVVRGTAINSNGRTSGITLPSAKLQEAVIRKAYSGADLSFDDTDYIECHGTGTAVGDPIEVDGLQSCFGPRKSPLVIGSVKANLGHSEAASGLTSLIKVALALDKGKIPPTYGVKNLNPKIHLEKANMSIAMDLGEWPRDIRRASINSFGYGGANAHAIVESLESYLDETHEVAPIESACTAQVLVLPLSAASKPSLETRLKQTEEIIQRGDGDLVKSLAFTLSQKSPGMSNKKFVLAKLRANGLSELVPPDSPEIVDAKSASLPFSFIFTGQGAQYPEMGKQLLQTSEPFLRTIRDLDGILKSLPKETSPEWTLEQTLLDSPLTSQINHVTRSQPICTAIQIGIVNLLRSWGVTPEAVVGHSSGEIAAAFSAGLLNAKEAILAAYFRGYAVGKLKSRGTMVAAGMGPDRAEQFIEEQDLRGKVCVACINSPESVTLSGSEEGVEILLAELQKNNLFARKLQTGGKAYHSHLVKEIGPLYESLLAPHLDGNSTLRGPAVTMFSSVGYESGKYLYLAKQARKAKYWRDNLENPVQFSSALQDLIGEGKTHLVEIGPHPALKGPVQHVRASIKVDKKILPYSPTLVRDQDADVCMKKLAGSLYLHGHALQWQNIQELPRQNQVILHNLQPYPWDYSAGVLWAESRPSVELRNRQHVRHELLGSAHVAGSGIDWSWRNVLRVNEASWLLDHKVESQIVFPAVGYLAMAMEAIAQIKGLKSAGSEKSARPSTSFEFRNVSISAALVIQDEQDMGTKEIELHTAISWRKISTTSKSTDWCDFSVSSWGSGQGTLHCSGSIRTGGPLPLKGTIEVQNTDGFEKWPMDRWYKKLAGEGLCFGPQFQSLTSLSTDGGRVRSDALATTNMKRRVGKETDTEYFVHPIAVDACLQAGIMGGTGGNLSTLKAFLPVFISECRIQSLGETNNEEATIHTNMTTTGFATRRIDATLLGPSGNVVVDMKDVRLSLYTGKMGDVKTSDSIHLERHPILRVNWKPDVLSLHPGAEPQLDQYIDTFISRQQPDLVDNETQAAIGALLDLAGHSNPRMRVLELGPDCDCKPTNWLSLLHKDTSLPRIQSWNTGSLADNGDLTVRNEASGPFDTILVSKLAESEKLWKYAPEKLISLVGDGGAIVTRRTDSALSSLAAAEFAVVEIRKQILLAVRRPNHKSLLDKDILIISGNKPSSAVVDFTKSLSSYLKQTAGAAQVNNVSIADLVQASVSSKMICISLLEIEREFLATMSPEDMNRLRMCTDVATNLLWFTGAGMLTTPDPNLTLSNGLSRALMLEQPSLRFTIYDVGPVGLLDNTSTFAYMDRILTVYEDVDDKEFIQVDGLLYTSRFGPDFRVNTEFRRRIGEQEPIEKEPLSTVGLAQLSIDKPGVTDSLHFQQIREVATTPPTGFIDVAIKAVSLNAKDIYTMSGHVETKTGTSSLEFCGVVTAVGPKVENVGLGDRVVVMAPNYFGTTERVPAWAAHKMLPGEEHSVLCTIPMAYSTAIYAMLDRGHLRAGESVLIHAGAGAVGTAAISLAMQIGATVYTTTSSKVKRDYLINELGVPDANIFNSRDASFVEGIRSATNGRGVDLILNSLIGDLMHASWDCLANFGRFIEIGKRELVDAGKLEMHMFLKNTTFTAFDLTELYYHEDQFYRDIWIEKTKQALELYRSGNAIPGPITKFDVSETAQAFRYFSVRDRIGKVVVSLENDESRVSVAPSKYLTVLDPEKVYLLIGCLGGLGRSLSRWMMARGARHFVFVGRSGCDKPSARELVTRLEAEGAHVTVTRGDVAQAADIVASVEACKATGKLIGGVIQAAMGLQEALFTRMSHEGWHTSIQPKWAGTWNLHNALEGNDEALDFFLLTSSVSGSVATATESNYCSANGFLDAFARWRRTQGKKAISIGLGMISEVGYLHENPEIEALLLRKGIQPLKEEEFLQVIDLSLSATGGDTEATDGRAPKIDCAHMLTGLEPLGLRSLMDQGFDVTTIGNMQDPRSAVLSAALLGNAESGDAGNASQAGLANAPSWLKEISAAAVKTLVSEADAISLIDAVLRVMRKRFSSAILMPVDQIDNAKPLAQFGMDSMIAAEFRTWIWGAFKVDIPFLDLLSNQKSLGSLAEFATEKLVEK

>Phain_OT5_Proseq2238

MATGECPIKKANVGGGGTRNNDWWPDELKLNILRQHTAATNPYKDFDYAAAFKTLDYNGLKKDLTALMTDSQEWWPADFGHYGGLWPDNVSLDKARRLLWPIKQKYGSKISWADLLLLTGNVAIESMGLPTFGFAGGRADQWEAEESVYWGGETTWLGNEVRYSEGNEGVKGAGVVDGDQHKKDHSDIHSRDLEEPLAAAHMGLIYVNPEGPDGVPDPVASARDIRTTFGRMAMNDEETVALIAGGHSFGKTHGAGPSENVGKEPQAAGLEKQGLGWESKFKSGKGPDTITSGLEVIWTSTPTKWSNKYLEYLYKYEWELTKSPAGANQWTAKGADAIIPDAFDPNKKHVPTMLTSDLALRYDPAYEKVSRNFLENPDKLADAFTRAWFKLLHRDMGPRSRWLGPEIPSEVLIWEDPVPAASHPLIDDNDVAALKKEILATGVAPTKFISTAWASASTFRGGDKRGGANGARIRLAPQKDWKVNNPKQLSEVLSALEGVQKKFQSGSKKVSLADLIVLAGSAALEKASGVAVPFAPGRTDATQEQTDAESFKHLEPYADGFRNYGKSTSRVRAEQFLVDRAHLLTLTAPELTVLVGGLRALNANYDGSSYGILTKRPGQLTNDFFVNLLDSNTAWKTTDNETYEGTDRKTGAKKWTATRADLVFGSHAELRALSEVYGSADAQEKFAKDFVAAWDKVMSLDRFDLASSSPQGKARL

>Phain_OT5_Proseq2261

MSTLLHQPNWVNGAYQSASKERIHVINPATESAIATIDSTPLETVNSIISSSLDNFSSGVWSKADASTRFNVLAKAATLLRLRLPEFIDLETQQTGRPIREMKAQLSRVPEWLEYFASLARTHEGRVTPFKGPVVNTLTRLPLGVVVQITPWNHPLLIATKKIAAALAAGNSVIVKPSESAPLSVLKMGQLFQEAGLPDGTLQIVSGYGRETGKFLCESSLISKIDLTGGLATYAAIAPTASKHLIPIIAELGGKAPVCMFPSISVEKGVKAALFASFIASGQTCVTGSRLLIHSDIYDEFVDLLVRRTKNLRVGAPTDEQTQIGAVISRASVERCSAFVSQSIEEGGKVLCGGSPTSVDGKGYFFQPTLIETKASSNLACNEVFGPVIALVRCESEEEIVKVANATAFALGASVWTNDFSQAHRVAENIDSGIVWINGHHLNDPSSPWGGFKESGLGKENGIEAFESYTKVKSTIINYGQEPTWFDDEVENARYG

>Phain_OT5_Proseq2352

MSFFKYGSALLLILAAVGESLIIPKSTVPVTDYDVLVIGGGPSGLSALSGLARVRRKTLLLDSAEYRNQNTRHMHDIIGNDGKAGTVPAVFRYNAREMIAKYPTAYMKNATATSIVSVNNGSSFVTTDANGTTYSSRKIILATGLSDVLPDTPGVKEAWQRGIFWCDWCDGYEHRDQSFGILGSIVDAVGSVIESETLFHDIIAFVNGTFTDENVAILDKNRPGWATQLQGYGVVINNASIKSIDRIQDGAVVHNVTTDTEYDKFQVNLANGSSIERDAFLTNFPSIQHSTLGSSLGVKVYGEKMIVDPGSMRTAALGVFAVGDANSDNATNVPHAMYSGKKAAVYVHVEIEKEHAATFVQKREVENIHERMGNDLEDLWKRLSSD

>Phain_OT5_Proseq236

MAPQNKLKLPKQLIDQLGLGDDTVTSHRKSGPPTGKRKDLRKAARAEKKVSRNGPSPAKRKKLYHVQEEVNSPFEDSIPENRKKQAREESESSHKPKSILKATKKADPPHNAPQPLKHTRPVRSVSPPAKISKSVKNKLAEDDEEIASLEKKLGLKGKKKLPQSFKDDGLDELLEGLDEGEDSEIPESKKRKAEGDEWLNRKRREAREARETSGQQFKDREEDEEDDLSDQNSEDDDGASDMSLDGPEFPDDDSEDDFGGMGDDGMILDADDDFASDDGDEEDEQPSRPRVRENPYAAPIANSDAPQKYIPPSLRKPASSDSEALIRLRRQTQGLVNRLTESNMISLLGDIEKLYRDHPRQHITSTLVDLLLTSVCEPTSLPDTLIILPAGFIAAIYKIVGMDFGAQIVQRSVELFDEHYRRSTVEPTGPAVLTTYSSKETSNIIMLLSELYNFQVIGSNLIFDYVRMFLENLSELHAELLLKIVRTSGPQLRHDDPSSLKDIVAMIRTAVTSAGGEHNLSVRTKFMIETINDLKNNKMKTGGAASAITSEHTIRMKKTLGTLNTRSLKASEPLRIGLKDIQGSDKRGKWWLVGASWSGDAVEECKPSAAGSVSTSQVEDAGTSDLVQLAKEQRMNTDVRRAIFITVMSATDYQDAYLRLTKLKLKKVQEYEIPKVLIHCCGAEKAYNPYYTLVAKKVCSDRKLKTAFQFCLWDSFRKMGESDDDEDVAEDEEDAMDIRRIFNLAKMFGSLIADGSLELGVLKKLNLSYLQSKTKSFIEVLFVTIFLQSQRQSQSKRDEQAVVNILSKAKDNPELIGGMQYFLRKVVSKTDIAGGKTEKETVRWACKLAGVTLEAFVAMDKAVIRPTLTLPTVVAPSAIARWSKRGYASEAEEKDLVIIGGGVAGYVAAIKAGQEGLKVACIEKRGTLGGTCLNVGCIPSKALLNNSHLYHQILHDTKNRGIDVGDVKLNLGQMMKAKDTAVSGLTKGIEFLFKKNNVEYIKGTGAFTGEHEIKVNLSEGGEQTVTAKNIIIATGSEATPFPGLEIDEKRIITSTGAIALDKVPESMVVIGGGIIGLEMGSVWSRLGAKVTVVEFLGQIGGPGMDAEISKAAQKTLKKQGIEFKLNTKVLGGDTSGEKIKLDVEAAKGGKQETLDADVVLVAIGRRPYTAGLGLENIGLETDDKGRLVIDSEYRTKIPHIRVIGDCTFGPMLAHKAEEEGVAAIEYIKKGYGHVNYGAIPSVMYTHPEVAWVGQNEQEVKASGVKYKVGTFPFSANSRAKTNLDSEGLVKIIADAETDRILGVHIIGPSAGELIAEGTLAIEYGASSEDIGRTSHAHPTLSEAFKEAAMATYGKAIHF

>Phain_OT5_Proseq238

MASTTTKPGGRAAILSRREVEGLIANGRYIIIVDQQVLKVDAWLQYHPGGDKSILHMVGRDATDEVHGYHSAETLKLMMKYRIGTIDGRWINFIPPIQGGKFRPYVEGVADDEEGQDEDKDLCSDISRAPSPIFDTEILSARHRGTSKNDRLVSSASSASSVSEPDDGMTWLDTLTRERIDLDIAKYPSLDLATQDEVVRKYRLLDERIRAERLYDCNYSAYAIETCRYTFFFSMFLLFLHWGWYIPSAAFLGCLWHQLTFTVHDAGHMGITHDFHTDSLIGIFIANYIGGLSVGWWKRNHNVHHIVTNSPEHDPDIEYIPFFAVSHRFLESLTSTFYEKLMPFDAVAEFLLPYQAYLYYPILTFGRFNLYVLSWGYLLRGLGPRKGPAWWHRWVEIVGQVFFWYWYGYVVLYKSIPTNADRFAFLMISHMITMPLHAQITLSHFSMSTAELGPHESFPQKMLRTTMDIDCPQWLDFFHGGLQFQAVHHLFPRMPRHNLRRTQALVQEFCIDVGIPYALYGVVDGSKQVIGRLGEVGRQAAILSKCQNTMAKRGDLLHGH

>Phain_OT5_Proseq2500

MRGAALAYVIIVRLLNLHFDTHEHRQPASQPASTVGNRIDNHNSDEAPEASYTIRDDQEGHHHSGSINVTEGQPRRRAIGAGMPTPKSPLDRPSSSHHRRPGGSRRSSSEKSVTKHGMKSGQAIQGNTKDSMSDPVSSAAKAPASTPTLTQTSSDKKDKRSYDANTVRWAESRAVVAPELSGADVPHAETTRDERGGNHKGSIGTGGSAIPKNTSIEELERVRAAVNAHIQLNQQRDKLSRHLSERGEQRNDAPRATGGKARVSPINEETAISSPSLEEAFASPPPLGPETTSTSSAATIRGASTPAAGTTARTPSYPFPSMRTPRQFSYTGHRPFTALSPTVNPANYAGGSFDGVQRDSVMSAPITPASTLNFQPPGTSQLRDPNFETPNLYDISLRLMSEPGLDSWWTTVAKIMSDSYKADRVTLTIPADPTDVENVPWAQKATFNINEEDDLSLGYMARGSSLLPSSVDTNETSNSEDAGDSPPLHLPSIHSLRPGLSSRHSFTAYEDTKREPISFSEASNGSAARPTMSRAKSYATSRHDVPPRTGTLQNAKLSLQSLEHHLESEDAKSAEWEDFEVAQREVRGRIFPVLQALDYEADQLIDNAGVMRVLDRGKGIVLTRDYPYIDKQESMDEKSGGSGSARQHSQRGRKSRASPNERPKRGKTPDLGTRIQSFLGSKGKHPHTRSLSRSQTSDKGRSIPTSASSHTHDQNDDAVPTAKYEEYEQRPPSPWSQSPAPSPAVRVETDENPFFANTSIDEDTFDPKTTTPREYSQSSQLEAIGIDRSWTVLHIPLMHPLLSKPVHAFRLDAAAMEQKSAGRGKPPDKKEKDKPKETEAVSDKQTPVAILSLLSPVIPYPSNLKHSLDFLAPHLATSYSLCRHHSNLQTEIAGLSRKGPRQAGFGAVVSGRRHLDDRVIFSQPVSSPIDDVGLQVSSGTGGSITSPSDYSGMSRSAAGSPAVTPGWEQTTVGIHRDKRHGGSPGNATGESYFSSRSRPALGRIDTGSASSVTGARPSKESSPADKRSHQHNRSIEGIPETHASDSKDNNEQGGKSDQDEVRSSTTTPRQEIRKHPERSAREIHSEASEAREASPSRHQAEPSASPRRQAIRMASSQSIRVDRPHTQLHSYGADFTATFQSLPPTTSVQPAKLQPPPKAQSRPGSVSTPTDMPPPSDRVKGLMLDSLPAHVFVAMPPTGEIVWVNNRYLTYRGQTVQDLYQDPWASLHPDERDEYLQAWTHAVRSGEQFSKQVRIKRFDGNYRWFYTRAVGHRDHRGVIVQWYGTHMDIHDQHLAEVKAARQEEIEASEAKHRQLANLIPQITFAATEDEGITFANEQWLSYTGQSFDDALGLGFMDYIHPEDLVKCHIPRDQPPTPRQAPKKKPAEVSRVPSQSSSTGQSSGQSSGKSSLVSDAPSEPTVKGIHQSLSRNNSSSSGSLYEFPIADLAELARTGVIKVTTDSDGRQSYETEVRLRSKTGDYRWHLVRCVEVENIALGSGDGSWFGACTDIHGHKLLEKTLKETMDIKARFLSNMSHEIRTPLIGISGMVNFLQDTTLNDEQSDYCQTIKSSADSLLNIINDILDLSKVDAGMMKLSYQWFHTRSLVEEVNELLYSLAITKRIEINYMVDADVPEMVKGDKFRIRQVLLNVVGNAIKFTTAGEVFSRCKVFKDASVGENEVMLEYSIIDTGSGFTEEEAEMIFKPFSQIDGSSTRTHGGTGLGLVISRQLVELHGGKMEGTSVPEEGSTFTFTAKFSLPSEEDHPEPPSTPMMSRDISSRASVQSLASKDPATAAQVLKQASSIGVIPDAQITQSPATVSPKTESSINSPAVSSSSSVPSVRSNRSYATHRSSSSSVAQSLAHFSQATRPGSTELAQMKLSLPEKQSSNSSVPAWAQNRAELGGMTPTPTSGASRRNSPSLSDLKHFRPPMYSILIICPQKHSREATTQHIEMTLPKDIPHQITALGSVAEAQEMIGGDDPVIFTHIVLNLGFNEEIVTLIDNIFASVSLPQTSVIVLSNPVQRQEIIRMATDYDYDQLAKDNRLTFVFKPVKPSRFAVIFDPDHERNLSTDRNRSTAQQQVADQKQSYLDVTQRLGNKGLKVLLVEDNLVNQKVLLKFLSKIGIAVEVALDGVECVNMVFSRPHGFYSLILCDLHMPNKDGYETCREIRAWEKENGHPRMPIIALSANVMADVMERCVQAGFNNYVTKPVDFQALSGTMGDLLDPLSS

>Phain_OT5_Proseq2671

MSENEEQHISELSTLLSHETIRIPEARVCPDNVQSLGIRSVTGLVTLLCFTFMIGTALITAPQIRILESIICRQYYERDPEFTGSLDDIDEHLCKNNKVQQDLTILSSRMLLLDAIVVTRLTHYKALFGIFEVIGHFSLPDNAFFIFKFVATSTYIIALALALTYAHLANLDVDLDQYFNHGLRKVPYVEICVHIIYHVSCSLPCSVICNYTNDKSGNSFFDAILLETAVLDTIRGYLVALFRDLPLQRRRAACDTPNQDDIVLGLFSLLFLISGASFVAWTPSKPVALLGVLTMTAGTGFPDLCRSVATELTTRPGVMSIVQFLPPLLARRVQVMAVGDYDVVTAVGRGIEDRLVFSHEQDRDAGRETAEWGRDTFCGANVLRKLCERHFGIIAPAQRDPESGSELERPTHYDRRNSSAAPSAIVAAIQATGRDAILRGSGGSNSAAVCILETHADTVSDPVRGLARMVQVSPKLTLIDLTIRGLSPGKYWATIRETGDISRGAVSTRGIWTDPKEGALKPRGFLGTVTVGKNGMGSVFIDKPIEIWEMIGRGLVVSKQHEGEGKFEKNDEDTLVGVIARSAGVWDNDKTVCSCSGKTLWEERKDEVRKGML

>Phain_OT5_Proseq2676

MDIALEFCDTYAFDYVYSALLPATSSPYNLNDGFSNATTFDAKAASSWQWQPASQLLSFAPTDAAYMSQWSRDNIYRQFLSLFLIMWLAGGLIYFIVSTLSYVFVFDKTTFQHPKFLKHQIKQEISQACRSMPTMSALTVPFFLAEVRGHSKLYDTLSDEPFKYYNYLQFPFFILFTDMFVYFIHRALHHPFLYKRLHKSHHKWIMPSPYAAIAFNPVDGWMQSLPYHFFPFFFPLQKFAYLALFAFVQIWTVFIHDGEYVANSPILNGAACHTMHHLYFNYNYGQYTTLWDRLGGSYRKPNDELFRRDSKMGEKEWARQVAEMEALVKDVEGEDDREYTEAPKLSLKKVN

>Phain_OT5_Proseq2735

MAPIRSLLIGLLPFVSAQGCPYAGANRRDLLVGRDDTSSLDTLASSFGKTSVISDAAGAGTRSKDWWPKQLRLDVLRQFSPEQNPLGADFDYAAAFATLDYEALKSDIMSMLTDSQAWWPADFGSYAGLLIRAAWHSAGTYRSIDGRGGAGMGQQRFAPLNSWPDNQSLDKARRLLWPLKQKYGKSISWADLVVLAGNCALESNGFPTIGFAAGRPDTFQSDESVFWGDETTFVPHGNDIRYNGSTDTTERADKLEKPLASTNMGLIYVNPQGPDANGDPKSSALDIRMTFGRMGMNDSETVALIAGGHAFGKTHGASSTTVGPEPEAAPLEQQGLGWSNSFGTGNADDTITSGLEVIWSKTPTNWSNDYLESLLGNSWTLVKSPAGALQFEALNGTLDYPDPFNKTFRHATMLVSDLALREDPSYNVIANSWLGDEGFKELTDAFAAAWFKLLHRDMGPRSRYLGPDVPKQSFIWQDPLPAASFPALSDADAEDLKTQILAAPGVNVSSLVSVAWGSASTFRGGDKRGGANGARIALQPQASWAVNNPAQLKTVLDSLNTIKTASGKVSLADLIVLGGNAAIEKAAADAGHTGVKVPFTGGRVDATQEDTDIATFEFLNTQGDGFRNFRNSSGWSLARTEELLVEKAQLLTLTAPEMTVLVGGMRALNTNYDGSSNGILTSSPGKLTPDFFVNILDINNVWTADSTNELYTAKDRTSGAQKFTATRADLVFSAHAELRAIGEVYAQNGGADNFVPDFVAAWTKVMNLDRYDVKK

>Phain_OT5_Proseq3181

MSVETITTISPTTNKPILTRNGISTSDLELLPQVATEAFQSFRKTSLAERQAIVKKALKLLNDKQDVLAKELTEQMGRPIAYGAKEVATAVKRGEYLLKISEDALKDTDGEAEKGFKRFIRKVPVGPVLILFAWNYPYLILVNSLIPAILAGNSVILKPSPQTPTIVEHISSIFTEAGLPQNVIQYFHCGSPLTIESIVRDPKIALVCFTGSVAGGLAVQKAAADRLVNVGLELGGKDPAYIRSDVDIAWVAEEIVDGAVFNSGQSCCSLERIYVDETIHDKFVEAVQGVLKGYKLGDPFNKETHVGPVVSKRSKEMIEAHIKDALEKGAKDETPDNASFNSPPSDGNFVKPTLLTNVDHHMTVMTEETFGPVIPVMKVKSDKEAIKLMNDSEFGLTASIWTKDTAKGAELAESVEAGTVFVNRCDYPSPDLAWTGWKNSGRGVTLSKFGFDQFVKLKNFHVKDYPK

>Phain_OT5_Proseq3339

MKIITSMEKLCIFNLEPAIGQTIAGRALVLGRCPLQEAELRLLCRREKAVVPLWEQACSNIANLFDANSQPYFQVQTHGRRLKLKASTALTTTKESKMRAQHGSTRYFCNLAGCTSTFRRQPDVVRHMKSIHGPKTPCPKGNCGYSTGRADKMKEHVQKKHKIPGGPAQGSPSIIDGRKNGTYDCMSSNNTDSNVTSSVDAFNTFGISSINASSFNSFSSPTNVPDFNVSNAVHFLDPINLSTMNPGMSMVTGQYGIDEWIYDRSFNPPNPWYIYPSWPGLTNAIPVLWEGVPAQGDLTPIIPLIQRSLMK

>Phain_OT5_Proseq3354

MSKPALLKAAILVVSTTASKDPSADSSGGVLSQVFEEEGAGKWEVSEVKIVGDEVLDIQIAITAWADRENALNLIITTGGTGFAVHDTTPEAVTPLLHKQAPGLVHGMLAASLAVTPFALMSRPVAGVRNKTLIITLPGSPKGAKENLQSILKLLPHACQQAVGADSRTLHAGGVKKLEKEAGVSAGHQPHSHAPDHSHGHSHGHGHSHGHAMPVRHTAPNENPRSNDPALGPTRRNRSSPYPMLAVDEALNLIQLHTPSPQVTSARVDGTLVGSVLSADVAATEAVPAYRASIVDGYAVIASEDGSSSQGTFPVASISHATPGDIPELKTGQIARITTGAPLPPGATSVVMVEDTVLKTMTDDGKEEKEVEILAEGVKDGENVREVGSDIKIGEVILRKGEEISAIGGELGLLASVGKAEVTVYKKPIIGVLSTGDEIVEHSRPGELRLGEVRDCNRPTILSAVRGWGFEAMDLGIAKDKPGALEEHLRDALRKVDVIITSGGVSMGELDLLKPTIERSLGGTIHFGRVSMKPGKPTTFATVPVKNNAGERVSKVIFSLPGNPVSAVVTLHLFVLPSLHHLSGVSPPGLPKVLVTLDHEFRLDPQRAEYHRAIVVMGKNGLLYASSTGGQRSSRVGSLKSANALLCLPTGKEPLKKGDKIEALLMGKVRIDVE

>Phain_OT5_Proseq343

MPALVEDVKPQGRLLLVSNRLPITIKRSDDGTYAFSMSSGGLVTGLSGLSKTTTFQWYGWPGLEVPETEVAPLQKRLKDEYGAIPVFIEDELADRHYNGFSNSILWPLFHYHPGEITFDESAWAAYKDVNRLFAKTIVKDVQDGDLIWVHDYHLMLLPEMLREEIGTSKKNVKIGFFLHTPFPSSEIYRILPVREELLLGVLHCDLIGFHTYDYARHFLSSCSRILYVEKPQDRSRSELIANRETTTTPNGVEFRGKYVTVAAFPIGIDPEKFVDGLKKKEVQDRIAVLERKFEGVKLIVGVDRLDYIKGVPQKLHALEVFLTEHPEWVGKVVLVQVAVPSREDVEEYQNLRAVVNELVGRINGRFGTIEFMPIHFLHQSVAFDELTALYAVSDACLVSSTRDGMNLVSYEYIATQRKRHGVMILSEFTGAAQSLNDALIVNPWNTEELAGAIHDAVTMSPEQREINFKKLEKYVFKYTSAWWGESFVSELVRISEHAEKKQKRSGHGQDS

>Phain_OT5_Proseq3693

MYRPFVMSNTAPEKTGEANTCPSSNRQIPATLPVNPWDPSQFPEGGIKAWLVVAGSFCCLFCSFGWINSIGIFQAYYQANQLRDYSSSQVSWIGSLELFMMLAGGIVVGRLYDSFGPTWILIFGTFFHVFGLMMVSLSTNYYQIILSQGICSPIGISCLFIPATTCTTTWFMKNRAFALGIVVAGSSTGGVVFPIMLDHLVFQVGFGWAMRVCAFIILVFLVFSILTVRSRIPPMPKPFKLSSYYQPFKEAPFVLTTIASFLFYLGLFLPINYIQVQALSYGVKSSLADNLIPILNSASIIGRVLPGYVADKFGRYNTQIVMCLFSGIIVLALWLPVTSNTGIIVFAALYGLSSGAFVSLIPALLAEITSMREIGLRSGLEFAILSIPALVSNPIGGALIEYNNGGFQDLQIWTGVILIAGGIIQLPVHASSAIQLPPKILPLAPLDHSRIMRLLEYKNDREFSLIDFGVEIPDSYAILSHTWGADNEEVTFQDLMDGTGKSKAGYDKIRFCGEQARRDGLKYSWVDTCCIDKSNHSELSEAINSMYRWYHNAAKCYVFLSDVSTNDSDHNPDQANPSLLSWQSAFRDSRWFTRGWTLQELIAPLSVEFFCSNGKRLGDKKSLEKQLHNITGIAAPAFQGVTPLMFSVEERISWAKHRQTKREEDRAYSLLGIFDIQMPLIYGEGAKKAFNRLHRELQQELYENSRKRHLDEVAPVSNATFNPAKRLKDSQNHRIEATTQQSLIDQLYFTRIDEHLTSLKAAQATTCRWFFTTPEYISWHDMAQQAAHGGFLWIKGNPGTGKSTLMKFLFESSKDNAKSDRSQITLSFFFLARGTDEEQSTTGLYRSLLHQLFKNGVDLKDSLEWMTSDGAESVERNGWHEEALKQTFTHSIQKLGNRSLMIFVDALDECDQNQVGGMVGFFEELCDCAREAQVHIQICFSSRHYPSIVIQRGVEVTLENEIGHTDDIKHYIKSKLRLGKSKQAESLRSEIFEKSSRIFLWVVLVLDILNSEYPNSVISIKKIRGRLQQIPQKLTELFEMILTRDGKKLELLQVCLKWILFAIRPLKPQELYFAIQLSLDKECSTYWDQEDMELDRIEDFVRSSSKGLAEVTRNKASQVQFIHESVRDFLLGKYGDQWAGISSSFVGDSHETLRDCCLAQLNASINQDITIPNPLPKASEAAQLREIISIKFPFLEYSVLNIFHHANSAQRDAMDQSDFLHNFPLEKWILHNNTIEKHGIRRYTPSVSLLYILAERNSADLIQIHPQRTSCFEVESERYGLPLFAALATKSHEAVQTLLQVQAAEVQPHDPLLHRLCTQYYESRNNPVNISRKFLFSQKKGVCFSVAELGEKLILAVICALGKFDVKSKDVSGRTPLSWATENGGEAAVKLLLKEGAELESRDKDSRTPLLYAVIKGQTAVVKLLLEKGAKLESTDKFGQTPLLQAAKNGHTAIVELLFKEGAKLESIDDSSRTPLLWAAENGHTATVELLLEKGAKLESTDDSGRTPLSRAIDFERVAVVELLLGQGAKLESIDNSGQTPLQRASHGGLVEMVKLLLEKGAILEHKDNTGQTPLHLASEWGRVDVVKLLLEEGADAAAIDGDGRTPFQLALKYNCTEIIELLGYDCFQITDIRARC

>Phain_OT5_Proseq4400

MVHSPSNLHEQGLTLLFGPQDPDINEAYTKKLRSTLLETPSLEWILDATTNLIHHWQSIFQTLPELLASPGRKHLEALNEWLRRGKFPEELFPLPNIIVTPLVVITHLLQYLGFLKEVHPELAPSDSLQAALRLPIETVGLCTGMLSSAAVASSENLAQLEKHGAVAIRMAMAIGASVDAGDVDTDLKGKWQSVAVGWTSPDARKELVKILKGFPEAYLSVISEEKLATVTTPKNVAAPLLKQLRDAGLIFVETALRGPFHCGSRNQEATSLIKLFDSDSNFQFPDVSLLAFPTRSATGAEYFAGDKLHHKAAQAMLTDQADWHQVFTSLQASATNSRSLVVAFGRERCVPQWVVRKLGPRLMQGANVDLKSCRLPPQFDRVLKSSGDEAVAIVGLACHLPGGDDLDGFWDTLCAGKSQHVEVPTQRLDFQTTAWRDYDSERKWYGNFVRDHDAFDHRFFKKSGREMASTDPQQRLMLQVAYQAVEQSGYFTMPTTPDANIGCYVGVGVADYENNVAGHPANAYTATGNLKSFAAGKISHYFGWSGPGITIDTACSSSAVAVHHACQAILSGECSAALAGGTSIMSSPEWYQNLDGASFLSPTGQCKPFDIDADGYCRGEGAGAVFLKKLSTAIEDGNQIFGVINASSVNQNENCSAITAPSVLSLSNVFSSVIHKAGLDPKQISVVEAHGTGTQVGDRAEYDSIRKVFGGPGRTESLSLGSVKGLVGHLECASGIAALIKTLLMIYNGAIPPQPSFNAISPKLNAMPSDNIEITKKFKAWDTDFRVALINNYGASGSNASLIVTQAPSFRSKDLDVERQSILKSPFWLCGLDGQSLQSYSTKLVQLLRSGKNTDHRFTIANLSFQLCRQSNRSLSQGLIFSCSSVDELAERLAAFAKGQGEISSVSRQVPPRPVILCFGGQASKFVGLDREIYDQIKLLRTHLDLCNEICLSLGLDSIFPAIFQKTPVEDIVRLQTMLFAIQYSCAKSWIESGVQVAAVVGHSFGELTAFCVSGTLSIKDTLKMIADRARIIQEKWGPEKGCMIAVDGNLTDVERLLQQTNNTNPDETTTIACFNGPRSFTLAGTSTAIELLREAITTNQASSSMKTKQLDVTNAFHSPLVDSIMPDLATVGDDLIFREPTVPHERASKDGTSALPIPGFVGSHMRYPVYFNHAVQRLAQRYPSAIWLEGGSNSGITSMASRALGSPKSSHFQPVSITGPEAFKHLTDATTNLWKEGLNTTFWAHHIAQTSDYSPLLLPPYQFEKSKHWLARTKRQVRNDSQAPQIQQAPKGLWNFMGYRDAAKTHARFQVNTSSEKYLTYVSAHVIAQTAPICPSTFQHVIAVDALESLAPMSNDRSLLPELQGMESSAPLCVDASKLVWLDAKRMGENDLTWDWSITSTTIGGNTSDANLHVSGRIVFRYVKDAAYDFAKYERLVDRERSLAILDGPEADQVILGSRNIYKAFASIVQYNHDEYRGLQKIAAKGNESAGRIKKQDEKGTALSVGLADTFCQVAGIFLNCMTDCGDGDMFLSNRVDQWIRSPTVSRDSRPELWEVYARHHSPSDKEYVSDMFIFDSLSGELVWVVLGLHFVKVPVNGMARLLSRLSSAAKTTRGSLTPPSTPPEMANAEDSHPAFSSKPAESIPLPKLARQKKVKINTTPRRDISSDARGLLCNLLGLDAEEVKPKSDLVELGIDSLLAMEVAREVENEFKTKLELEELMEMTDFQSLVDCLKYKLGISDNLAAEEEGEDEDDESEAGIQTPADSAVSSYNEISGHVNGNRTSPTTIPASVILDTFADSKQLTDHFIEENNFSGYMDQVLPKQNELVVVHIVDAFEQVGCSLRTAEAGQVLDRISYLPKHEQVMAVFYGLLEKARLVDLDGSKITRTAIHVPPKSATTLLQELLSESPEHSYDHKLTSLTGSKLADCLTGKTDGIQLIFGTPEGRELVAGTYALSPMNVAWIAQLEHFWKQLLLRLPSQSEPISILELGAGTGGTTARLVPILAASGIPFQYTVTDISPSLVAGLRKRFKHHPFMRFQLLDVEKPPPAELLHLQHVVLATNCVHATHSLVRTTKNIHGLLRPDGFLCMLEMAKNIPWVDSVFGLVEGWWLFDDDDRVEQQHALAQPSLWEKTLRSNGYGHVDWSDGELPENTIQHIIIALASGPSYDRVPILPKRLSNHTTNFAARQAVVDSFVDKYTRHFSAPVSLPVSDLVINLSRCVMVTGATGSLGSHLVEYFASQPDIHKVICLNRIGSIDGAVRQRQALESRGLLLSKEASSKLQVLETDTSKPMLGLSAADYKSLAKSVNLIVHNAWAMSMTRPVRAFELQIKTMRNLIDFARECACQRQPKDPKIGFQFVSSVSVMGYHPFVSGKALAPEERVAVDSVLPMGYADAKLVCEYMLDETLHQHPDNFRTMVVRIGQISGSKRNGLWNPVEHLAHLIKSSQTLNVLPDLDGVLSWCPVNDVAATLGELVIGDTTAYPIYHIENPVRQPWRDMIPILAEALGVPQANTISYKEWIRRVREFPPSLVSDNPAARLGDFFNADFERMSCGGLILDTAHSKEHSDTFRGLQADSYGSNTLD

>Phain_OT5_Proseq4412

MEKVLDGARQKASEAVSGIMSTNKKTADMKPNMVEPTSSDRLTSDFGIMKFDHERIPERVVHARGAGAFGTFKLYESAEDVTNAGVLTDTSRSTPVFIRFSTVLGSRGSADTVRDVRGFAVKFYTDEGNWDIVGNDIPVFFIQDAMKFPDVIHAGKPEPDTEIPQAQSAHNNFWDFQYLHSEATHMFMWAMSDRGIPRSYRMMQGFGVNTYTLTNAQGVRHFVKFHFTPTLGVHSLVWDEALKIAGQDPDFHRKDLYSAIDAGVFPTWKFGIQVCPESRQDEFEFDILDATKVWPEELLPVRYIGELQLNKNVDEYFTQTEQVAFCTAHLVPGIGFSDDPLLQGRNFSYFDTQLSRLGVNWQELPINRPVCPVMNNHRDGQLRHKITKGKINYWPNRESAVQPASQAEGAYIDYPEKIVAIKQRLHSVKFSEHFNQAQLFWNSMSPHEQAHIINALGFELDHCDDPVVYDRMVERLCDIDIDLAKAVAEKAGSPTPTKQGKPNHGLKAKGLSQMDFTPEALGLPPTIASRMIAIIIGPGFNLVEYEGVKAALSAAGALVFTIGPKRQPVLSAGGKKSVAPDHHFEGMRSTMFDSIYIPGGEHIAMLQKQGRVVHWVREAFGHCKAIGATGEAVKLVQTACGVEGMAFSTNAEVVDSYGVVTAAGGFEPSGVKEALSMVKGAKGFIDAYAYNISQHRNFQRELDGLTSMVAY

>Phain_OT5_Proseq4534

MAETSQPAQESAGLDGGILEPIAVVGMSMKFPQDAVTEESFWQMLLEKRCAATEFPEDRLNIDAFHSPEAGKRNTISTRKAHFLGEDFRAFDAPFFSIPPLEAATIDPQQRGLLEVTYRALENAGIPMEAVRGSDASVHVGSFTSDFKTMVWRDAQQIPKYSATGTAGSILSNRISWFFDLGGPSMTVDTACSSGLVALDLSCQGLWTGQSRMGVVAGSNLILSPELNIALSSMSFLSKDGQCFSFDQRGNGYGRGEGFGVLILKRLSDAIQDGDTIRALIRSTGVNQDGNTPGGVTQPSRVSQAALITDTYRKAGLGMGLTRFFEAHGTGTAIGDPIEARAIGDSFRDQRPEKETLLIGAVKANLGHLEGASGLAGLIKTILVLEKGLIPPNASFENLNSNIDADFLQLEFPLQCTPWPSEGPRRASVNSFGFGGTNAHAVLEDVRSYLLARNLVANHCTVESALIQRRNTLTNSVNADQLIPKLLVFSSADEAGMRRLSQVYSQYFAEPGRNDNTQRFLDLAFTLNSRRSALPWKSYLVATSISELAEMEQIVSKPLQASGQKPNLGFVFTGQGAQWYAMGRELMVYPVFKASLLRAQNYLQKLGCNWLLTVELARDKSTSRINEPEFSQALCTAVQLALVDLFESAGVVPSVVVGHSSGEISAAYCNGSLSMESAMKISYRRGALASKLAREAKSKHRMASIGLSQAQFTTELEKLKQEGLELNSLTVSCINSPKNVTVSGPDAQLDILVAHYDKTDVFARKLMVDVGYHSPQMQEIAGEYLASMDNLTRGTRKTKTIMVSSVTGAIIDADVLCTGEYWVQNMVSPVNFLGAMKECCSRPGSTSPTKKLDRSHLREIVTHALVEIGPHSALQGPIRDILASVNRAGEVSYASALVRNRSAVESLLATAGRLHCQGISVDIDALNLLKTKDSTVPRVLVDLPHYPFNHSTVYWEESQMNKEFRFREHLNHELLGNQAPGYNELEPRWRLIIRSDELPWVEDHKVNGSILYPAAGMLAMAIEATKQLTGNDLPLGYEIKDAVFHSPLLISTSKENVETQICLRPTATSSNSKSIWYDYRMYVHKTDEGWTELCTGSIRADYGKTQSEVDHGVEGPMFLDTLRRNHSGAANRCNGLIESSDLYRMLKEMGLEYGSAFQPLHDIRYSQDGEATARVALFEAESPAATEAQRHVIHPTTLDGIFQMIFVALSKGSAAGVQTMVPSRIDKVWVSNTGAGTPFKGSLEAYTKSHQFSKRSARGLVSVLDSEGQLMVHVDGFEATAVSTQTNADTQEEARQLCYNMTWKWDPETMDKSQIQQYCKTLARPDLQNAERYKDAEFMALAFGSAALRKLRVQNRLPCPRMEDYAVWLQMALDDDLASLSVKEAAKRRAQIEDPTYLGYVCKHLASSSLGQLFMRIGHHLPNILSGDIDPLDVLFSDEYMFDEFHRELTEGSGCFDALGKYLEALAHKNPGMEILEIGAGTGATTAMLQETLASQPNAPSYGQYDFTDSRPFFVDRARETFKPYSRMAYHVLDLEKDIVNQGLKEGSYDVVVATSALVSYKDSGETIRNAQKLLRPGGKLILGQITVGDAVRPGFANGLLPEWWSKSPETPCVSEEKWDEILKENGFSGTDIIFRDFEDEQFHDYSIIISTAKPVAPVRPFPGEIVAIVESNSESQRVISQYLGQELRKFGYNLSKILTLEQAASLSDHNIHHYIVMHDLSAPLLRNLTSDGLSALQTILSSAGSLLWVMPGGRQGPVTPACGMVEGLCRVSRQENPNVPLVTLALDVSPRTRTENFACNIARVFHLTVSHKETGDFEPEYVEMDGCLHINRLSTAASLNDHIYVRTNYPRREQKFGDLPAVKVDVRTPGLLDSLEFIEDGKASLPLGPNEVEVEVRAIGVNFKECLTVLGRVNTDTLGNECSGVVSRVGENWDTLKPGDRVALCATDTYRSFARSSIGCAAKIPDSMTFNEAAAIPTAFCTAYYCLIESARLEKGESILIHAASGGTGQAAVQIALHLGAEVFATVGSKEKKQLIMSQYQIPEDHIFYSRDLSFADGIKRMTQGRGVDVVLNSLAGDQLVATWECIAPYGRFMEIGRRDIDNHGNLPMYSFLRNASFTGVDLAAIVQQRPQLIQKMMREIMRLVEAKKLGPSYPLTVFPISDMEHAFRILQSGKSSGKIVLEATTDAIVPTSLKTRPSYELSKDATYLIAGGLGGIGRSIAKWLVDRGANNLILLSRSGPVGNEKGQALVTELRAKGVRVVAPACDIASLSALKSLVQRCAKTMPPIKGCIQASMVLRDTTFASMSFQQWEESVRPKVQGSWNLHVSLPPGMDFFVLLSSASGIFGNPGQSNYAAGNTYQDELAHYRVLHGEKAVALDLGVVLSEGVVAENSQLMDRLMRQGVMLPITQEEVQSLLDFYCNPALQLSPQWCDQVILGIDIPAKVVARGGEVPVALCQPLFRKMHQIEFSGQSSVRKDENSTDFKTMFASVGTLAEAGMVVSEALRKKLSKVLGIAEENIELSHRVESYGVDSLVAVELRNWVSKEMSADLAVFEIVGGATLVGVGLTAASKSAFKQSTWT

>Phain_OT5_Proseq4716

MSPSSSSSIGGKINQEIDSLPPAIAEKLPHVPESKEELKEDLKQAETKSLQGLRSLAAGGFGGICAVVVGHPFDLVKVRLQTAERGVYSSAIDVVRKSIARDGVRRGLYAGVSAPLVGVTPMFAVSFWGYDVGKGLVRRFSTVQDNQLSVAQVSAAGFFSAIPMTLITAPFERVKVLLQIQGQKQLAPGEKPKYSGGVDVVRQLYKEGGVRSVFRGSVATLARDGPGSAAYFAAYEYIKRRLTPVDPVTGKASGDLSLLAVTAAGAAAGVAMWIPVFPIDTVKSRLQTMEGKPTVGGIVRGLYKNGGVKAFFPGFGPAIARAVPANAATFLGVELAHQFMNKTFG

>Phain_OT5_Proseq4847

MAAIQNGSFTSPLADAKASQILGSPQLDGILSKVSELSGWTIALTILAVLVAYDQCSYLWQKGSIPGPTFKIPFIGPFLESVNPKFEGYMGKWASGELSCVSVFHKFVVIASTREMSRKVFNSPSFVKPCVVDVAHKLLGATNWVFLDGKAHVDFRKGLNGLFTRRALEIYLPGQEDVYNAYFARFLKVSKDAGNKPVPFMPEFREVMCAVSCRTFVGHYISDEAIKKIADDYYHITAALELVNFPIIIPFTRTWYGKKASDMVLEEFTKCAAKSKVRMAAGGDITCIMDGWVKSQIESARYRERQEKGLSMEGIEKPPHMLRDFTDFEIAQTVFTFLFASQDATSSACTWLFQITAQRPDVLDKIREENLRVRGGDRDVPVTMEILDEMVYTRGAVRELLRYRPPVIMVPYLAKKAFPITDSYTVPKGAMIIPTTYPSLHDPEIYENPDVYDPDRYVTGDAEVKGAKNFLVFGTGPHYCLGQVYAQNNLALMIGKAAMHLDWVHHATPLSEEIKVFATIFPKLDLEETRLKAASPLLFFHFISKKIQASKATPYVQAVPKTLASGFDMLFRTPPDGV

>Phain_OT5_Proseq4931

MAPQTPTPPAPSMAKASGSKKLDPKKPHITDERITKANWYKHVNWLNVTLIIGVPIYGMITAYYTPLYLKTAIFAFVYYFMTGLGITAGYHRLWAHTCYSARLPLKIFLAAVGAGAVQGSIRWWSRDHRAHHRYTDTDKDPYSVRKGLIYSHIGWMVMKQNPKRIGRTDISDLNDDPVVVWQHRNYIKCVIFMGMVFPCLVSGLGWGDWMGGFIYAGILRIFVVQQATFCVNSLAHWLGDQPFDDRNSPRDHVITAFVTLGEGYHNFHHEFPSDYRNAIEWFQYDPTKWSIWIWKQLGLAYDLKQFKQNEIEKGRLQQMQKKLDQKRQTLDWGIALEQLPIIDWDTYQTEAKNGRALVAVAGVVHDITDFIKDHPGGKALITSGIGKDATAIFNGGVYNHSNAAHNLLSTMRVGVLRGGCEVEIWKRAQRENKDISFVSDSAGQKIIRAGSQVTRIAEPVASADAA

>Phain_OT5_Proseq5001

MLRVLAKSRSQSQVLRTNFKSSTTVAIGMAPASKRSNGSLPGGYREDSSKGPMLRFEESLPKLPVPTLEETAVRYLKSVHPLLSNSELASTTKAVQEFIKPGGVGSKLQEKLLARREDPKHKNWIYEWWNDAAYLTYRDPVVPYVSYFYSHRDDRRRRDPSKRAAAISTAILEFKSQVDSGTLEPEYMKKLPISMESYQWMFNCCRVPAKPADHPAKYSPAENKHILVIRKNQFFKLFYEVDGKQLNTSELEQQFKTIYQKAERAPAVGLLTSENRDVWTEAREVLLKAYPSNAAALKDIESSSFVICLDDASPVTLEERAHQYWHGDGANRWFDKPLQFIINDNGTSGFTGEHSMMDGTPTHRLNDYVNEVIFNNKLDFSDPTIRSSLPDPTPIKFKVNKEVQAEILRAEKDFTEVIGAHELRVQAYQGYGKGLIKKFKCSPDAYVQMVIQLAYFKMYGKNRPTYESAATRRFQQGRTETCRTVSDDSVAFCKAMNDSSLDPKEIAALFHKAVKAHVEYISAASDGKGVDRHLFGLKKLLGPGDEVPAIYTDPAYSYSSTWRISSSQLSSEYFNGYGWSQVVDDGWGIAYMINENSIQFNVVSKGLGSERMSFYLNEAAGDIRDLLLPTLEPAKAKL

>Phain_OT5_Proseq5092

MSTSGGLAGLPLKPNLGFNWVFLVELIVCGILTLFFLFYLNRLFATLVSFGLRAWTWHKYRVYVDIQALQISLLGGRVFFKGVKYHGNNETILIHSGFVTWCYWLRNVRDLNVGRDDDVGRKRVSSDSDSDGRRNPHQNVDKVEEGGARGQGNLPCRLNVSLNGVEWFVYNRSAAYDSVVAAMTKDDKENVEAVPTQQSGHRESASKLRKRDKVSDDVEFASKNSVDLDRDTSRSEKASFMMPEADPKSHESSASQTETSGGHGNENGTEGAFILRFLPIHIECNKAAVVLGNENTKSVLITKATRAAGVIDGTKSKTLDQYRQLINFQFEHPVIQMKPNDDYKEDQTAAAIRIKLGEADQPQPKEHHAHSHTFLHRQRRKAWHTLQDLIPAFRSSVDSISSSDEQPETSTQARPGQNSWQGLSRYLDEGEQDDKARWSSVEYATVSTILDSPQAYMSFYWDVVGTVPDQTVQADMKRDHDSSNINGDPPPEWGLDLSLKGAIVNYGPWADRQRADIQRVFFPSLCKDATPAKNLMPGQFRVPTEFKLYIEFDDETTLRVPTKEESKNWKWTKQADSMGAQREDQKNRGIRGRRRKADKGSPGPEIRPFGWVDIKIGANATVAYTMDMVAGPSGFSNTLELDLPNTEITTSVNHGLLWKSVDNRLSCDLSNPLQWNGPRTWKFDINSSGLELFILREHIFLFIDLIDDWASGPPPEYLTFTPFDYLINLHLEDFRLYLNVNDSNIINNPSDFDDNTFIVIYGSVLNAKQCVPLHNYRPYRNDVPFDVDMKHGGLNLHVPPWNTQATFLNSTELATLKELKINGKYQYCATTSTSNTDTLLLDVHGYAPTAQFYGFVIRYFLKIKDNYFGDDIRFKTLEEYQDVLRARREGSDDSSNHPPNKKSNDLDVIIAVTADESSVILPANLYTAKEHVRIDIATLAADLRFTNYYMDLDVVLSTLAFSLGSEQDGSATPISATSSTQLFIDGVNANGNRLFGLPPTEPTYICNWDFAVGAVTGECTIDFFGRLSNGARAFAFSFDDDENALPSISEVILHDITFLRASVESIRLWLHVEDAAFLLSSGTISVNFNDWAGSHYSKKLKLLIPDLKVGCVDAESASRHRSRAQHPVETHALLQTTLSVAMIQRKLGFEKDRQLQQEHVKRHDQRTHRADFLLYPSLLDDSMLEPGDPPAMSVPPLPIPVSVDEVAHQENRSLASRKSSRLLGKRVVSRKSSFLSIRSSGQKSNSSIIRPQSSWRSREQLQPDSRSRSVQINSSADLRTPLRDISASTDHRSSFYSAVGDHSRGMPPSSVTFSSSYIAPYFPLEGVEPDSKDLPPMSADTENLDSKETQFSLGDIAHDRVDEDSTHVSFMIELPTGIAVVCNPEAVKAFACLITALQAVDPIDIIDEIQVDSMSEIFDLKRQKSITGTVLDFSVRTPSISLRFINSSGNLGYQQAMHDQYDFSMTRLAITSRSETSVKSFTPQGARKSSTAHLSLASADLSAKERYNDIDDPQAAINGTLEDVACWFKTDGNTSAAVAFKGIQVATASSKIEYLASLLHRTGVLANDIVETFSSVISKQRNRVQLFTYLVATAGQQATDPLFLTRPSYVLRSAPDHLRTTDSWKIITRLHHMYTSLHASTKHDIIMRCLNNTESVPDDARQRVGAGFDQWRSWDLNDTNSCLVMSRIFGAKDDVSSTSATSSPLKLSFRTELIRLVLDPGPKQNEISLIDITSTFESKTAPFEAEAPSPTDDTSTTQTSIVQLFCGDASINLNWELCELAEDIMKLYRQSEKRSKPKRAESPSPVKTSRTSLTPGHNLHVVVATERGSITIDTINIRATSICEGLNASFVMVNKSGIEVKKSATLMLAAEAATSKIRHHSQELTVYQIRFPSIFGSYGSQIIKEIPVNLLNVAGNCQQLTFDVKQDILALIEVLDVFIGDEVAHLYRLKKQMPSTTPSQVQASEQPRKPTPINRINVALFLDMYDISIPLLQSLTYNISGVVARASLAARSDSEVVFDFDIKEHSHDMQTVMATKHKSISLLQMPPTNGHITCYMLEKETTVSVFASVEPVELDAAAVHSLLTALNRPEMSSVVSDVKEEVKVVQAHLEEILGPSKKTIKKDVKESKPLIYDAHLTLAGFDIFANAQAREDSNSARLEFKLGCVQLVLANRVEQNGPVLAFPELRASLQHIMFELSRPKNLGIMESCGNLEFAACLTATSKLSDTGDVVRSFHLKSDALKINLFAETASSVVDVMGYLQDKIKDVDLSREKNYFRKLRKSRLHSTTNDEQPEAEGSKAGSTGRFSSLYSLELLNIQVCWLVDTHDKNKTSQLEEEDLVLSLKRIALSTKRENTARLTIEDLQLQMVPASHDKIQRSLNSALLPEVIFNVGYVSTPDARRFAFQAAGKSLDLRMTSQFILPAAALQKSIFSATEKVRAATATWMTQTPGPPTESSRRQPFFGKKRMESLLVDADFAGAVVYLSGKKVHEASKATSNSKGGRAPQAGRYGQFSQNDANSNTVLRAPGLAWRVEYKDNGVDEPSLNAEVKVDASSNILFPTVVPLIMEISSTVKEIVSDDDEKKKLLQPKANPQKFMSADEDNILTADPSTVLGRTRLNLGVRICRQEFSLSCQPIARVAATARFDDIYMTINTVRSTEHGHFFAISAAFRKLEASVQHVYSRESTGSFEVDSVFLSLMNSKHVSGTSGVSAILKISPMKVLVNAKQLQDFLLFREIWVPPEIRQSSPTPAPTSLTAQSQTHLVQRYQQVAATGAFPWNATVSIAELDVQLDLGQAIGKSAFMISKFWISSKKNSDWEQNLCMGFDKVGVDSAGRMSGFVALQDFRIRTSIQWPAREMALNQTPLVQGSLGFSQLRVKAAFDYQAFLVADITSFEFLMYNVRNGPHAKGDRLVAILDGEAVQVFCTTNTASQAIALYQAFERLVQEKRANYETSLAEIEKFMQRKSHQHPTSTPARDSLHPLGEDKSAKSPISLHTDVIVTLKAVNVGAFPNTFSDHQVFKLEALNAQARFAVTMDNGKIHSILGLTLGQLRIGLAGVKTIDVPKSVGELSVEEVISSATGSRGGTILKVPKVEAIMQTWQVPDSNHIDYIFKSFLEGKVEVGWNYSRISYIRSMWASHSKALAQRLGKPLPFSAVKITGVPDNESGRRKDGEQQKITAEVNMPQSKYDYSALEPPIIETPQLRDMGEATPPLEWIGLHRDRLPNLTHQIVIVTLLELASEVEDAYAKILGSF

>Phain_OT5_Proseq5217

MATVVQGSIPPSIENGTLVAQHGISSQVNGILSGWSSLQIIATVLLILIAYDQFAYIYRKGSIAGPMFKIPFMGPFMQAIHPNFNEYLLQWASGPLSCVSVFHKFVVLAADRDIAHKVFKSPAYAKPCLVPIAEEIMRPTAWVFLRGKAHAEFRRGLTGLFTNKALATYLPVQEKVYGDYFDKFVAASEANGGKPMAFMGLFREINCAMSCRTFFGDYISQDAVKRIADDFYLVTAALELVNIPFSIHVPFTKTWRGKRIADAVAAEFTKCAAACKANMASGAEPKCIVDQWVLHMMESKKYNDRIAAGEVDIERPVNLIREFTDEEIGQTIFTFLFAAQDASSSSTTWLFQILAQRPDVLDRLREENLAVRGGDRHRPFDLEMYEALPYTNAVVKELLRYRPPVIFVPYLATKAFPVSPSYTVPKGAMIIPSCYPALHDPEAYPSPEVFDPDRWITGDAGSKTKNWLVFGAGPHDCLARKYVPLTMAAMIGKAALEVDWVHHATSRSEEIRVFATLFPMDECQLVFTKRK

>Phain_OT5_Proseq5268

MSYGNSMQDLAALPSPARHTLTSLKLGFFNIVETLRYSLPAMDSLTRTLPASWFCSSALYQLERRAVFLKAWHFLGPVTRFQSRSEKVTYEIAQVTVTVENRSPEHDGIGIDGITVYAEDENIKIKSHLTPSGLLFATLSEDTPSFEDFFEGLEELTNKVDFTKLPHRRSISYEGNFNWKTMIDGFQECLHCQYTHPTFSKFYPPTFYSVTNHKNFSQHMADPSKQSDGLFLYFFPICTLNVYGGGMSSFRTLPSTKPGVSRMEFDYYHAGTDEVFEEYFKFVRQVAIEDFELCEKAQENLERGVYGEGILNPVKENGVQFYQQRVKKLVYKQHASEKSAKDEHINATQVPVGVVEESKVIAVQ

>Phain_OT5_Proseq5388

MTVVKDHSQPVSNGPSSDSQSHTQAPLAVIGMSCRLPGKSNSPSSLWEFLERGGIASNTTPSSRFNLDTHYVGSPKPNTMRSPGGMYLENIDPQEFDAPFFNISREDAIAMDPQQRQLLEVVYEGLENAGITLESLDGAAVACFVASFTCDYGDMLGRDPEDRPPGTLVGLGRAILSNRISHFLNIKGPSVTLDTACSGTLQGIDIASRYLQTREVTTAIVAGANLFLSPEHNMDIGNMKTAHSLTGRCHTFDKKADGYAKAEAVNAVIIKRLDDAIRDGDPIRAVIRGSATTSDGRTPGIASPSSEAQARAIQSAYANAGISDYSITSYLECHGTGTQAGDPIELNGAASVFAATRPKDDPLRIGSIKSNIGHSEPSAGISGLLKTILALEKGIIPGNPTFDDPNPKIDFENLRVRPSKATIPWPDVPYRRASVNSFGFGGSNAHVVVDETASYLGREPDAHCSSYLSGEDNLFAEDDTVSRPVTLVFSANDDKSLKSYVKALSDHLINPNVQIKLPDLAYTLSHRRTRHFHRAYLVTQDPTNIDQGKLVVGKKGLGVPKIGFVFTGQGAQWPRMGKSLLDTFPSTRILLKKLDDVLQSLPDPPSWSLLGELTEPRKPQHLRQPEFSQPIVTALQLVILEILDNWGVKPEAVVGHSSGEIAAACAAGYLTQEQAIKIAFYRGRAVVLDGSKASLGMLAVGLGPEKVQEYIGDLYLLVQIGCFNSPNSVTLSGDSAALETVRERIDKDKHFARLLQVDVAYHSKFMESIAACYKELLLQNCDISADAHAIDTVTMYSSVIGRQLDYACDAAYWMTNMESPVRFDQAVQAMLSAPAGAPDFLIEIGPSGALAGPISQITALKGSSVQYCAALTRGKDSIEALFAVAGRLFISGGNVDLAKVNADERDLSGRQPRLIVDLPNYVWNHSTKYWHESEASKDWRFRKFPHHDLLGGKVLGTSWELPSFNKTLRMKDQPWLRDHGMGPDIIFPAAGFISMAIEAMYQSHNALKPDTDVSSVDQLSYRLRNVRFDKALVLEEENDAKVTLHLSPHPGPKETWYDFKVVSLNEGSWTEHSSGLIRLETLSNEVVSKDKLAPLKYAKPGSEWYKAFNTIGYGYGPEFQKLLSVETVAGSLTTRNHISLTEPASILTQSRYPMHPCTLDACIQAFIPSVWRGDRSAPDTVLLPAIIDDLVITNAAKTSDIGIAIATSKYSGRGRKDDKRNWYTDISMYDPETGNMMLKINALRYHKLPTGTDVGAEHTITRSMWNPDIDFLSAEQLANLSVDKSGSTVQRIIDLIAHKKPALKVMEVDLGSTDTSSIWFAEVNEELRPTRALYSTFNFLTADANNFVAVHDDLSRHRDSTTTLVDVTAVDFTFEESEFDLVILKGDKLNQRLLKSVLQNVRGILSDGGYAILAENSSIITDSGSGSEEDVVVVNSENILNADQIAAIATANDFYRTATVSCESCTSVNIFVAKPKITVLPKTRELVIANMTDQKPTTEMTTALEVAGWTILESNQPLEDIKPKSTVLIQDELFNPVLKSVSSAQWDSVKYLVSQGCKILWLTQGSQMNVTTPDNALIHGMFRTVRQEDTSLRLMTLDVHESTGPATIPAVVKILEIIAGTLPKTFVDNEFVERNGVVFVNRIVPDKLVNQFKDDEGGRGAQPVVKSLHDVQGIANLRAERLGSLDALQYTEAPLTSMEDEHVEIEIVAAGLNSKDITTTQGNTPGNEHLLGMEGAGTVTRVGKAVKTFKVGDRVAMVTQATFANRVQCPVQYVHHIPNSLSFEEAATVPIAYLTSMYCLFKVGNLQKGQSILIHAAAGGVGIACIYLAKYIGAEIYVTVSSDEKRSFLKETFGFRDDRIFSSRTTDFARKIKASTQSKGIDLIINSLTGELLDESWRICADSGTMVEIGNKDILDRQYLSMEPFDRNCSYRSVAIQKLSPSTVTSLLSRCFDLLSGGHIKPINPATRFGFDSIPEAFAYMRDGRHIGKVVITDGEKGMTTTVPIRPAMHEISLQSNVSYLIVGGLKGICGSLAVYMAAHGAKYIIAMTRSGISDERSQAIVRDCNALGCEVQEAKADVTNTEDVESAFKAATQPVGGIIQGAVILRDKPFEAMTLEDYNTAASAKVQGTWNLHRSSLAHKSPMSFFTLLSSMSGIVGQKAQANYSAANVFMDAFASYRHSEGLPAHALDLGIIEEVGFAAREGGIQERMDNRIWLGVNESMVRRMFKYSVLQQTQPINPESATQLIIGIALPQQKGSDLETDARFSPLFSNHNSGDQHKGSKAGDEDAKAVQTFLLLHRNKADKSSLVAAGLEVLSMQFTKTLRLTEAMEPAKSLSAYGVDSLSAVEVRNWVRVELGVELTTLDITNASSLFGLCEKMIAKMPAVGECVVPV

>Phain_OT5_Proseq547

MPKTSDQKNAELSDTPRPLILPDRQESIHPALDPSRTNVPVTPGFLPSEYDAEGSDHPSETSYFAHDINKVRSAIAQSPTAAASGAKSNQDLLRRISLTSGLGRQDSLVDMDPRAANPSLSLSGGIISATFCIPHTLKYRKGADWGLDSRRGTSALFDSFAYLSSDKTPWNHTLVGWTGEIIPADEELTPPNTPPANPNKIPLNKNSAPIPVDAFAKPVEPATSEGLHISKEDIKALETQLARDKKGRTVPVWLADDTEGVSDGSLYLTDQSRWRRFAEHELYTLFHYKQHEPTNGRAERQTWADYYRMNQKFANRILEIYKPGDIVMIHDYHLLLLPSMLRQRVPHMYISFFLHIPFPSSEFLRCLPRRKDILEGVLGANLIGFQAYSYSRHFVSCCTRIVGYPSDTAGVDAYGGKVSVGVFPIGIDAAAVQHDAFDNPVIGEKVTALRELYKDKKIIVGRDRLDTVRGVAQKLMAFERFLDMYPEWRDKVVLIQVTSPTSVEEEQEDTGNKIANKVSELVLKINGVYGSLEFSPVQHYPQYLSQEEYFALLRAGDVALITSVRDGMNTTSLEYVVCQRNSHGPLILSEFSGTAGSLKDAIHINPWDLTGVAQQINYALTMGEDKKMAMHSSLYHHVTTKNVQAWSTGYIKRLLTVLSAHNTTISTPLLDKAVMLTQYRAATKRLFMFDYDGTLTPIVKNPSDAIPSERVIRTLKALASDHRNAVWIISGRDQEFLSQYLGDIPELGFSAEHGSFMRHPGSSEWENLAETFDMSWQKEVMECFQKYTEMTPGSFIERKRCALTWHYRPSDPELGANNARKCQTELEKTVGKKWDVEVMTGKANLEVRPTFINKGEIAKRLVADYGSEVGEPPEFTLCLGDDFTDEDMFRALNGSNLPLDHVFTVTVGASSKMTLAHWHLLEPSDVISSLALLNGGDSDSADLGPLAVVEGRIPESMPGEKH

>Phain_OT5_Proseq5572

MRTATIITLFSAGLTVATPLHQLFHAHAKKALVTDIVTDIVTVYVTATGEVPTPAAESTVVVAPTSVAVASTSAAATSIAAVFAAVVSPPVKEHVHSHDVSVAVSTEAAPTTTSSSVVEVSIPATTSSAAPTTVAAPTSVAAPSSVVTPSSVAVVASSSAVAPVASATDYASTALFNHNQHRSNHSASDVAWNQTLADFAATSAAKCIFKHDNDGYGQNIAAWGASNGVPADSNAVANAITEAWYNGEVGDFPFNVASITLDSEGLYNGHDVLHFSQVVWKGTESVGCASQTCGPDTVLGSASYNSVYTVCNYYPAGNVGGEYITNVGTATGAVAKMTESISV

>Phain_OT5_Proseq5677

MAPISVTEPHASEALEPVAIIGMGCRWPGDSESPSELWDFLEGKQHAYSKFPKDRINGDAFYHPDGSRPGSFKTEGGCFLKSDVRQFDANFFGIHPKEVLTLDPAQRKFMEVVYEAFESAGVPLHKLSGSTTGTFVGNFNYDHQLMQYRDAENALPYSVTGSGVTILSNRINYVFNLKGPSMTLDTACSSSLYALHLACTAIQTGDCTAAVVGGSNLILTPECQIFSSVLGAVSPTSVCHTFDSRADGYARADGIGALYIKKLSQAVADGDPIRGVIRGTSFNANGRTGGITHPSPDGQEACMRRAYARAGGLDMSLTGYFECHGTGTPVGDPIEVSAIGRLFSDVKTSSKPLLIGSVKSNMGHSEPSSGIAGIMKAVIAIERGVIPPTIGIETLNPNVDLKDGRLKIVTESTPWPDLPVRRASVNSFGYGGSNAHAIIESAETLLPGYRSRHQASKESSGMEAFANANGISRLSSSSSLTNSQTNGNGHTNGSSNGNGYHSPNGNGYRSKPTKESKKNLLLPFSAHDDKTLQANYDALAKAIGNWNLADVAFTLSARRSMLTFRSFIITSPEEAKQDLPEQKLSIAKRKSAAAPILGFIFTGQGAQWPQMGQALMREYPSFLVTIRRLDKYLDDLDEAHGRDWSLEEILNQDPEDSQVHNAEFSQPLVTALQIALVNLLSRWNVTPHAVVGHSSGEIAASYAAGLLTEEAAMIAAYLRGQMVAQNEKTGLMMAVGGQLSEIQPLVDGFDGQIVIACHNSPESYTLSGDTDAMLKLKAVLDEKKIFCRALSTNNNAYHSHHMKDLGSRYEQLLDSLMPKNTAAKSIKQSSKSKSTQANLESPVLFHQAVEEMVTQVPVDILVEVGPHAALQGPLRQLSKTLDSDIKFPEYLTAIVRGNDNAKDALTLAGNLFTRGYDVDLAEVNATEGRGGNQSQYGKVITDLPHYQWQYSKEIVLFENRYTREWRLRMHPRHDILGSRIPGANKREPTWRNMLSANNVTWLTDHRLGKDIVFPSTGYLSLVLEAATQIAEVEGYQAADVEYYDIRDVSLSKALIIPEDDRGIETLFTMRPASLNTVSRHQWLFEFVLTSVSSEEGQDIFSEHCRGLVEVSFEHHDFPENRMVNDQASSSVKKIINAAQWYETFARVGLCYGPVFQGLSNIYAAGESNMSEAQVGLKPTAKTMKGESRYLIHPATLDASMQLSILAAHKSVATKFERAFMPTSFESIKVWPKMARQSDTSANSYASANLKGVRGLSADIVLQDMEGHRMLEATNIFLTASDQSAPKLIDDPSPYTRIVWKPSFDHLTTGIMEQMYPPVILSDDAVIPSLNQLALHQLIHFKASNPDIFARGSDVPHLQRLLDWTTEKLNIAGADLNSGAGKIMEYSHDFRAQEIERLTDILTPQSSEARLMCHLYKNLPAIYSGEKTGIQVALQDNLLLANYETGQVYREGNRRLASTVALYAHQNPGLKILEVGAGTGSATNELLPALKGDSPWRQYLEYRFTDTTTSFLSDAEERFAQFGGMTFGAFDMERSGESQGYQQDWDVVIASNVIHATSDIKSTLINIRNVLKPGGKMILLELTQAQLSAGLVLGTFSDFWKADHDPGYPRYDGPFLSKQLWRTVLPEAGFQGLDFYLDDYAGDNISTTVLCATVAEPQTSIPRAITTSENEGITMVYRETPNSFMDPMASTLSSGGVAVEVIPLAKVGTARYRRFVFLLEASNPFFIDVTPQEWSDLQSALKLSASSLWITKGDLLAGREPLHAMISGLIRGMKTENSSLRFNILDLDKTPESSDTKLFSFVQELESRVSDTTRISDDSEFRYKDGIVYISRLTADDILNEKSKAIAQKSSATEKVPLKDLKTTPLQLAIEKPGVLSTIYFKPDPVFATPLGEDEVEIEVRFSGVNNKDIAVLTGRHHSDSSSDECSGVITKVGQSVKDLKVGDSVYCQSYSKFGNFVREKAAFCQRLETRDTLEGTSTLPIAFSTAIYGLEDLGRLTKGESVLIQSATGAVGLAACQIAYMIGAEVFATVGTEEKKKELLSMGYGIKETNILWSRDRFSAKKLLQQTGGKGIDVILCSARGELMHDYWRCIATCGRFVEIGRTEILDNGKLSLDVFRRNATFTSFDLEVMSKEKPQVTTSLMKRIQQLKSQGYIKPLPVQRFHVSEIDKAFMTFGKGTHIGKLVIDYDDMSDQGLNVRQEPFTTKFDPNASYLLVGCLGGLGRSFSAWAVSRGARNLIYLSRTGAAKDEAKIFLDGLKKNGVDVSIVKGDVTSLSDVQAAVKSSKLPIKGVVQGALTLNDGLFESMSLERFNATVLPRVTGTLNLHEALKDSPLDFFEIWSSWTVIFGTATQSNYLASNSFLDAFARHRRSLGLPCTSLALSQVLGIGIVSYMPEYQQAMIRNGFYGNDENEFLQYCEAGIMTPTPGETQDPTFKYDPQTLGHLLVGIEPAGLQNVDRKYPLSEMLWYRDPRFKNLTQATSLLSAGNQDKHAASGEEGTALDRIRMKISRLLYVPLDEVDIDKAINDYGIDSMIAAELRNWFFASFSKDISLLKLLSATMTVQKLAEEAESEDKGE

>Phain_OT5_Proseq5771

MSVIQVEDLVSYQLRTNYLNTIADGVGERLITINDSFLNTAGFKAAGWRPNAANIKRTHSPPIPTAIASEYFQAAPRSAGLPTGLEDENEEGGMVTGGGAGDTVGPGIATKRRRRREQMEEEDSSDLSDDSDEEGDQRAAQQIKFSKMPIRNRSGSSPIRGSNLRQTSTIASPTRTPGARRGSQSALEAVKERARRDTVTSSEMSSENEFDASAFTRQRDATRNAAKASRALHSQTSDPNVGVKRQQSDLLEEEEEDDSDVSDMDSEFEGSIDSTSILDSVDGAPIKASPSDAIIGTVPKELTRSPTVKSKPAPPVLQALPPPRPISMVQPKSLLSAAIKARQTKAASPFESFASLSGQGEPNPLLLRIWAPFATSTTKPYEVLIRRSVHMNEANDRQVTVADLIGLSLWRYAEEKLEPVIPPEKLNVNWWTLRMVEDEEVDYDFPALERNKPLVSFTTENNRAARSRSNSKPFDEFALVQATDEQFQQNQSLTPQFKQESVQTAPAEDDFPSRITPTPQLSNIGASPLLSTQNRPNPLLTTMSALRQNQSLADLPTPGGAPSQPTARLGAQKLLRIHIHSADAAPGQMITLDISTDTYLAEVLDVVCKKRQLDKANHVLKLTGSGTVVLLDRTIESIGNRADLDLYRRRFATDGPLTMSGSPSSSSPKKPMMADRPEYGKIRKGQMLGAHPLAQEAVKQDELGNSNYRKYIVWRKQPMRFVGMNERVIAIDGEYLHIMPSSTGKTMFEGQGKTTTVHFSNVVGCKVTRRHPTNFKVVVYKATETKRYDFEAKSADEAAEIVFEIKKGFSPYHNV

>Phain_OT5_Proseq5870

MAPKALRALTALLASASFSQYAFAAPTGTSTAAVPTALLGYNPANVVKNDDTDDIRYTLVPGQTNAAVVGTYLDFTNVENPQPIRGTKGGTDPGPHTDAYAQLNPDKLAPPGTDHGSVDNAQWPLGLSHEKLGLNRGGWSRQQNIDNIPAATDMAGVDMRLEEGAYRELHWHKAAEWSYVLNGSVRVQAVNEKGETFIDDLNAGDVWFFPPGVPHSLQALEGGVEFLLVFDDGEFSEDNTFLATEVFTRNPKEILSKNFDVPLSAWDDIPPGELFIFPGTKAPTDISKQNVVGSAGILPREDSYTYHLSKQGATFDLEGGNIKILDPTTFPIASMFSVAIVTVKPGALREVHWHTTSDEWNYFIAGSARIGIYAAVNNAQTFDFHAGDTGYIPKQMTHYVENVGKDDLMFIEVLQADHFSDMSLGQWLGSTPPQIVQDTLNLTNTTVSAFKKEKQYIVSGDVAE

>Phain_OT5_Proseq6240

MRPSLPLLLATSALSNALAAHHQKRVSGTSIVNLGNNTGTPTHLASGILYGIPDTAGQIPGTLYEDMGFNYARAGGAQVAAPGRGWIWGVAEYTNSTAPYPGDDGDWSSWDAYLTRWISDIKANDATTGLSIDIWNEPDLTYFWNAPQAQYFQMWGRTYHRLRTELPSVKLIGPAFAGQPDASNSWWTGFLSFVASNASVPDEWVWHMEGGGGDMEGAYGALFNLLKTYNLPNHPINIDEYATYDEQVPAGSAWWISQLERVNAIGLRGNWLSGSQLHDFLASLLSKPDATTSAYSATAGGYFPNGDYQVYKYYNLNMTGYRVGSSPSTDLKLDTYATVGSDTVKVLTGVRITTGTWQVEINDLSAVGLATSGTLNIHTWSFPATGGHFGEVDGPTDLGWASAQLGIAYAGKSESGVPRIDDGQVGVGLNTTRHDTTRHGAKETVEHWKPSYQSYYCEIAGIIGELKDCCRIPRFRFHFISGGDPKPTPITKNVTTLSIPPIDPINHPTNMSSPTVSLSWPSSSLFRFPPSNDPLVLPPPQAGSTLSAPPFSIPSSLYIAALNVKVPITIASIYAVTVVSLNAYNRSGGNKPWRISKTRAFFCFVVLHNVFLAVYSAWTFVGMLGALRTTVQRPSAPAGFAGTVDSLCKIHGASGLGNAIAYNGSESKWISQSPSTILLTGAGAPDSTDVGRLWNEGLAFYGWFFYLSKFYEVLDTAIILAKGKRSSTLQTYHHAGAMMCMWAGIRYMSPPIWMFVFVNSAIHTLMYTYYTLTAFSVPIPNTLKRSLTTMQIIQFLVGVAYAAFHSFVSYSIPVRVPYLKDAISVTANATSSAAATATAASIMDIIKKLLFRAVGGEGVAENVNAAHTPGIIEQSNSLAGTAQYRTEYKTVPCIDTNGQTLAIWLNVFYLAPLTFLFGRFFVKSYLRRTDKMTGHASENVEKAGLDAVKGVERTFNGGLANGGKMNGNGKANGKH

>Phain_OT5_Proseq6247

MASAKYSLPPLPYAYDALEPSISAQIMTLHHTKHHQTYINNLNATLASQATATSKQDIVASLHLQNALNFNAGGHINHTLFWENLVPASSPNAQPTAAPKLAAALTARFGSLEKFQDKFTAVLLGLKGSGWGWLVQDSETGNLELTTSRDQDIVPVGKKPILGVDFWEHAYYLQYLNDKASYAKGIWKITNWKVLEARYESGYERVFGPLAGLRSSI

>Phain_OT5_Proseq6335

MSAPPPPPPHGHNPKSSGLPDGKYDIFIIPPHSSGSGFLYLPSLQPNVNSFAAGFASALFLVVVGQALAPAVQQWWTSIKGSGGMGMILLMMAIGVGAWALGRTQSDGGPGPGSGPGGGNPPPNPGAHSYTNANAHSNGYASGPPPASEPPPASGPPPASGPPPGAGAGGPKPSSWQRTNPPPGATGASAKPSPASGAAKGAWEKAREETKRKEEERKVKEAAQKRKEELEKRLKEAREKEAREREAREKEAREREAREKQQKEDEAKAKAKQKEDEAKAKAKLEAEEKEKQRAKEKLEAWEREKQRVKVKLQERLKAKLEAEEKEKQRAKEKKEEEAKPKSSSYAFSATGERTNPWPKGRPPSPVTQRSQPPSPTKRPPAPTAKTYLGTDEDGYSFRPYDRPNKPMHKKAPSSIYSESSYVSQSTSRTTPPPSHRGPYSTKDPDKIVIKAVYAFNNAFLKTPTSQLVSGVGSVTDGLVLRITTEGLFIDDDVRGVPQREWDVKAWTMKLVEVWCPSFRQGPAGNSNFASARVASHSAPNTNKTFRRLWGLDKDKAATSEETDALLIDMLQLCRDNCRLRATSRSSAASSSAYSSTASSNSYDDRSSVSDSAYGSSVGSSSPSSGKRTDKGLAFQTGELKSARLHVLRASIRDQEGKKYVFIITEEESWKVAVGLQKLRKGSQVRALGVSGMSPNDAKGTLEHLGWV

>Phain_OT5_Proseq6431

MSHRNSCDRCHQQKVRCLRNESRTGERDTKIPAHFPPAQCERCTKAGANCVYSLKRSRRSSPGPSLHKNSKANLSDDISRAQWLEYDLDGPFFADSSIGEFETADLALLAGNGFGGFNANPPLVSPISLPGSNFNFSAGPSGPVYPADGAEAAEAHSSSWGAEAGSVSQSPSPTEPEPGINDGEVCEKEQNSADTDTLSSQLIALSQRSMRAARRLGRPGLAPLTVLSPEVNEALEDTNTLLRIIDSMGCTPLDRDGKKPSPTSTDHGLPFLALACHQNLVSLFQVMCNAIQQVLQSKKEHQHQQNQSGQYKDVGASSVAQFAMVLHLLTHLVNRIDRSLFPNSHLISHNAGMSTGGHSTTIHSKEEAPGSLPRGKLLVHVNNIAGSISNDHRKLKEVTQKLQTEIEHSEFVE

>Phain_OT5_Proseq6433

MADISGVLLSPSFVICAVLVLLIGNTLLFVISTLRPKHFPPGPSGFPGLGNLLQVDRAFPFLTYAAWGKTYGLDTPIGVKKGAANIVVLNSTRPIHDLLEKRGATYGDRPPTFEMNNSVVFRDVPRTAIMQNHSPWLVQWRKEFHGAFGAAAVKRHHPVYDAEAARLLIKLIEFPKVTGKDLESILVCWLISVPCIGVCGRRPDYMSDLGFTVDDFRHCITEYTKLIVPSIRDVFPVLRYFPWLSGLTEWKEKAVGVREGYLRLSSQFVSAAKEQREALDAGRSITWESMIAKMMRDRRDKKDTFSENELGPTAIQVVTAAMNTSTAKFSTMLMVMAKYPEVQQRVRNEVLEASEGEAPKATDIPNLKYTEAFWNEITRWRPVAALTPAHAPSKDDVYNGYRIPKGTPILMNNWNISHSEADYEEPDEFIPERFLRHPFGMRQDEAHNPSRIEASGGRVTYEFGAGRRLCPGMAFAKQTLLLGLAKILWAFEILPPEGKEIDVSLEGFVQKFALEPKNLDVVFKLREGLSEKDITEHYSQAYDAEAEVLGWQNGLYK

>Phain_OT5_Proseq6434

MADKDLPRLDVEFPTLDGLTLRAWLFPASKRGPGMIMTPGFNMPKDAQLPDLAKWFQERDITCLLYDPRGIGASDGQPRNDIDARQMAEDLHDAVTWLLKEPLVDETKIALWGLCFGGNVTLAAAAFDKRIAAMITIGPVIDSSGNPDRRQPILELVMHDRAMRLEGEEPMYLPYINEDGGIANGLEMPADMMPALERLGVPVENRISVQTYHKGMSWNILSLVEYISPTPVLMVTPEFDKSCPTADQLKCYALLKEPKELDILKGKGHIDWLFGDIESVLNRQLDFLKRRIVF

>Phain_OT5_Proseq6435

MKALVGAKSGGYCLENIEIPVPQRGSMLVRVHAVAINPHDAKIIDYSHTPGSLGGCDFAGTVAKLGEGVTRFKEGDRVLAVSFGSDALDKSKGAFAEFALAEEDISCHIPEAFSFTQACSIGLSMATAGLALFQAPGLELSMKGGMGQTVLVSGGATATGTMATQLLKIAGYTPIVTCSPANNALCESYGAATCFDYHSPSCGADIRGHTDNSLRHVLDCVTDTDTMKMCYEAVGSSGGSYIALEMISTTVKYTRRDIHADWLLANAIIGDGCNMTGAYGRPPSPEHRQFGRQLFALAEKWLHEGSIKHHPLEIKNAGLANIPKAIEELKLGIWHARKLVLPLESY

>Phain_OT5_Proseq6436

MGSLKIDDFALEPIAIIGSSCRFPGGAVSPSKLWELLEKPRDVVQEIPASRFNTKAFYHPDSQHHGSTNVKHAYLLDEDPRAFDRDFFSINPKDAEAMDPQQRLLLEVVYEGIESTGYSMPQLRGSSTGVFVGCMSFDYQYTTIRGIDSLPQYSATGMAASILANRVSYFYDWTGPSVTLDTACSSSLVALHQAVSALRNGEVKMAVAAGSNLIIGPEPFITESKLNMLSPNGRSFMWDSSADGYTRGEGVSAIFLKTLSQAIADGDHIESIVRGTGVNSDGRTPGITMPGSESQAQLIWDTYARCGLNAACETDRPQYFEAHGTGTPAGDPVEARAIHNVFFPDGKEGQLLVGSIKTLIGHTEGTAGVAGVLKASLAVQHGRVPANLHFKNLNPKIRPYYTNLRIPTETIPWPAVPHGSPRRVSVNSFGFGGTNAHAIIESWDGVGSLANSSAMNGHASKVQAAGPFVLSANSATALAASSGALASYVRAHPETDLSQLAYTLFQRTDFPFRAAFSATSTEQLADKLEASKESLKKSSRTATIPEVLPPRILGVFTGQGAQWATMGKELYSSSELFRRAIEQMQRSLDSLPTKDRPDWSLVDQLNAPAETSRVGEAAVSQPLCTALQIALVDILRAAGVEFSAVVGHSSGEISAAYAAGYLTATDAIRVAHYRGVHSHLAQGPGGKRGKMMAVGMSLKQSTVFCSEFDGALVVAASNSQTSCTLAGDAEAVDEAQARLQENGTFARVLQVDTAYHSPHMKPCASPYLESMKQCGVKVQKGWKHCRWYSSVLGPNGRGRSFDQADSVLLEGQYWVDNMTQTVLFSQALARALNEDQYFDLALEVGPHPALKGPCSETIKMLTGLALPYSSVLKRDQSAVESFSNALGLIWQLFPSSYPMITFDGIRRAFMGGKPRKLTILKSLPAYSWDHSGLIWKESRSSRIFRTQSQPRHELLGHSITHGKHDKCEVHWKQLFRVNELPWLAGHSIDGEVLFPASGYLAMAYEAATRLVDEKEPLHLVELHNIDIVRAMRLEEDSSGLEVVFTIRITSRSNDCITAEVACYSGAVDSAQPLDAPQAGLTVHFTGGVRLWLGQPHKDDLPPRIKPLLPMDTLDMGQLYSSLGRVGFNYAGLFQAPSMLRRLNHAVVTLSSPPENSQIRACMHPAPVDTAIHGLFAGLSFPGDGRLGSIYLPTRIESVRISMMPSKVHATELTADAAVTSTDSTTIKGDVDLFDAAEARTVVQIRGIHLTAVGQRPDPWLYAGTKWVRDINYGIEPGSGADISDAEVVLCEQLSRTAYFYLRELRRKILPQEMLLMGKHRKHMMSWVLKHLLPQIEAGEHPDVLPEWKNDTLEMVHQWRASQSPDNIDMNILNAMGKSLVGIVRGTTPSLKVLTQDGMLDRLYVEGVGLKHGNLDLAAMIKQLAHQHPRMNIVEVGTGTGGTTRAVLNAIGNQYASYTYTDRSDFFESAGTVFSQHGSKLAFKALDIERNPVEQGFTDGTFDLVVASNCLHATRTLEGTLRHCRQLLRPGGRLVLLEITRDFLPMQLLMSTVPGWFLGIEDGKLSAPTISIEQWNELLKANGFSGVEISSSPSFCSAIMASAVDETAQLLREPLDVAPEALKSLPCRDILIIGDGVSSKLASQTQAVLRSALPSKTITLLTVLEGIQTPEGAAVLCLEDLDSPVFRNMNEKKFRGLQDIMGKAEAILWVTSGAKSGKDPDANMTVGLSSTLRLERTDLKLQFLDVDNPALLDPSMLAKMLLRLTFVDPSNTEELLWTQEPELWLKDGALYIPRVLPLDTVNRRSAARHRKVPQAVSLESKDTVVILDRSSGAFGLQAVPLSTTKGDVMRLQVKASSMNTLSCDEYGPVYLCIGYDISSGEKVLALSGVNSSLVTISEDYVLYRWRDSISTGEELEELHTFLALAQARHLLRNLKGATWIHGAVNHLREAINVIARGQSLAVFQTTSDIAKASEPNFIHPYANERDIQHMSPKDLQTFINLEAFGNEGLSTLMLGLLPASANVSMQVADLAVGLSLSELRSLAKQQVRVNSQVTVLGVETLSIGEVSTATSKELRDAVVVDWSTADTVSALVPPLEHRGLFSPDKTYLLCGMTGDIGISVCLWMVEHGARNVVLTSRNPNVSTSVLDYLSQKGATVRPMAVDIANIDSLRKAYHDIKSSMPPIGGVMNAAMVLRDRLFTHLPWEEFAAVLTPKVLGSKNLDEIFQNEQLDFFICFSSTTSIGGSIGQSAYAAANFYMASLVQRRRQHGLAGSVIHIAILTGFGYIFRRDSEHAETIYKAVLPRFDSQSEGDLHQLLAEAIVCGRPNSGQTAELITGIKTVFQGEWRDDPRLSGYIGQQEEQDDSSKAQTGYVSVKDQLAATEDPAECLVILEHCFSQALGKLLETDPETLDSNTTVASLGIDSLIAIRIREWFLKETGVDVPVLKIMSDSYSISQMCNDVLVGWRKLNKS

>Phain_OT5_Proseq6439

MSSYVVTGVSRGIGFEFVRQLSENPASTVFGLVRNKAAVESKVAAEIGRKNIHIIEADTTEPEALKKAAEYVSEKTNGTLDYIIANAALQAKTAMVGFDTLSKDPKALEEDLMEHYRVNSIGPVHLFNIFMSLILKGRAKKVIAISTGISDPEMTLKADIFQATSYSMSKAALNVAVAKFSALYREEGVLIMAICPGAVETGSLQIETEEDGKLAMAMFGKFKEYSPTFQGPAKPEDSVKSVLGLVNKATVDGGYAGVFISHTEGKPYL

>Phain_OT5_Proseq6910

MSPSATTTIPVGEGTNGSVGVTPDTHNNGYVNSATNGQSNGNGPSNGQVEDPTLYTTGYTHGYTTGYISGVAQSDTPQQMPIAIVGMSCRLPGNVTTPDEFWELCSRSRTGWSEIPKERFDNKSFFHPNPGKSGCQNTIGGNFLKEDLGLFDAPFFSLTAQEAISMDPQARILLECTFEALDSAGIPKHEIVGKDVGVFIGSSISEYEAQLFADSESIPMYQASGCSMAMQANRISHYFDLRGPSFTMDTACSSSLVAIHQACQSIRNGESKVALVGGCHLNMLPEMWISMSMSRLFAEEGRSFSFDNRGTGYGRGEGCGMVVLKPLDQALKDNDTIRAVIAGSGINQDGKTPGITMPSGEAQESLIRSVYRTAGINPKDTGYIEAHGTGTKVGDPIEATALHNVFGEDRTARKPLFLGSLKSNIGHLEAASGIVSVIKTAMMLERGFILPNYDFKKPNDKIPFSKWGLKVPVSQAPWPRAKRFASVNNFGFGGTNAHAVLERAPFSKKNETEAADPLDFQSRRLFVLSANDKTALEAMMKNMGIYLEQRPEIFQSDLMANVAYTLGQRRSFMQWRVAISSPTSFELIETLNSGKVSPSRETETRIGFIFTGQGAQWNAMGRELYAQYPVFAASLDACDQCLASFGATFSVIAELNKDAKTSAVNEAHVSQPACTAIQIALTDLLQSWGVSPTAVAGHSSGEIAAAYAAGILPLDSCMAISYQRGMSIVSLKKKFPDLKGTMMAVGGSKEEIYPLISQLKTKEVRIACFNSPSSLTLSGDVSAIDELQTMIEQKQVFNRKLQVDVAYHSHHMKLIAEEYQESLRTLVPPKSTNVKFYSALLGHLVDGAKLQPSYWVDNLTQSVRFSEALTAMCAPADGHKTGVNMLVELGPHSALAGPVKQILKTCGPNAMKIPYVSALVRNKDAVETAVNMAGTLFVKGAKLDLGAVNFPRPRKPPTLLVDLPRYPWNHNTKYWHESRMTQKQRNRATPRNDILGSLAVYSNDLEPTWRNTIRVDDLPWLRHHQIQGLTLFPMSGFVAIAVEAASQRAASKGTDFDKFILRDVSVHAPLMITDKDVETTIQLRPFQEGTLVSSEAWDEFRIHSWADGQGWTEHCKGLVAVKSNKSEEFDGARIAQDSETLLRSTMLEVNSAKTNSVDKKVMYDSLSELGVSYGPTFQVENCKANDSCSTAQMTVADTAQEMPQGFQTGVVIHPAFLEQLIEMYWPILGAGRTSVNTVYLPSSIGRLSISRDVTELLERPGNTLRAFCKGAAPLLHPKPTQVSMFATAGDDSAEALITLDDLTISPIIERGLASEADAYRELCYKLDWEPILQPVSDESSNKSSPELSNGTSNGNHVNGNSNGVVKEPELLGEDSKEETNGVSEKVNGSAIPEPNGSVSNGPSNGVSSDSSANADEPAAFPETEIVIIHGDSDSQKLLATKLADILELSTGRRPIAGALAEIDSSEKICLFLSELDKPLLSNLSESDFLALQKTLTTVEGVLWIVRGAYAASVNPDANMVAGLSRSIRSETLLKFATLDLDSRIPLSDELTVKAILDVFKAAFGSKAETNGELEFMERQGSFFTPRIINDAEMNEYVHKHTNASVLEATRFSQPERPLKMVIGTPGALDTLHFVDDQTLEAPLADDEIEVEVKAIGMNSRDIMAATNQLETDGFGVECSGIITQIGNQVTDLAIGDRVASVTVARGVYSTYTRTKAAFALKISDGLLFEAAASIPVAYCTAYYGLIDLGRLGSDESVLIHGAADPAGLAAISLAQMVGAEVFAAVGSIEEKDFVSSTYGLPDDHIFSSRGASFGPIIRQATNKRGVDVVVNSVATDTDTLRELWGSLSSFGRFVVVGKRDASARLETNRFDNNTSFISVDLMSVAAERPKIMERVVSNVYALLQNGKINPAASITVFPISDVETAFKVLQSGTSLGKLVVAPQPGDEVKATLSNKLNMLLRSDATYILIGGTGGLGRSMARWMVEKGARNIVLVSRSGCATGKVKELIDELATVGANIVVRRCDVTNSSSVDNLVNNELVGLPPVRGLVHGAMVLDDVLFEKMTFDQYTTVMESKVRGAWNFHNALKNDSLDFFVTISSAAGAVGNRGQAAYAAANTFLNAFVQYRVALGLSASSIDLTAVSDAGYLAENLEAAAEVAKNLGSDTICEAEVLALIGAAISGRLATACNHHTITGMRITSSTPFWTPDAKFKHLRLAAEAAAAENSTGQAAAISFHAALKASKTAEEGQEVVCKGLLSKLPSVLMLEEEDMDVTKSLANYALDSLVAIEVRNFITREFEANLQVLELLSSGSIETLAKTIVVKSKLVSF

>Phain_OT5_Proseq753

MACIPRPIAVIGIGCRFPGGANSPEKLWEVLSEGRDTWSEVPEGRFKWKSFHHPDPEAPGAHNHRGGHFLDEDIAAFDGRFFGIPPQECEALDPQFRIQLEVTYEAIENAGLHLEHLRGSETAVYVATFGQDYAGMQDRDLDTISKYYMTGRGLAMASNRISYAFDLRGPSVTLDTGCSGSMVAIHQACQSLRVGEAKIALAGGVSLIITPDLMVPMSSIGVLNKDGKSYSFDSRGAGYGRGEGAAMIVLKHLDDAIRDGDNIRGIIRNSGVGQDGKTPGITVPNREAQLNLIQSVYRQANLNPHETKYVEAHGTGTIAGDGAEIAAIRGAFASDGSTPVHVGSAKPNVGHLESASGVAGLIKAILMLENGAIPPSINLVELKESIQGSDISIPVTPEPWDKTALRRISVNSFGYGGTNGHVILDSASTAAYLTNEHVQSLYDEEPYPQVLVLTAKSEKSLVKALGNFRQWLSQNCDLGGDAFRNLAYTLSTRRSLFRWRCSFTGTSPKDVLSRLSLKSPKPTRSVNKTQNVFVFTGQGAQWFAMGRELLGFESLYLKSLRDSDKILKELGASWSLITELLRDVSSSRVNESQIAQPATTALQIALVDLLSSTSVHPDVVIGHSSGEIAAAYAAGALSQRSALKVAYYRGLLQVTGSVKGAMLAVGLGEKSVSQYISQVTSGKLVVACSNSPESSTVSGDEAAILELKVILDTASVFARKLAVDTAYHSHHVESVAEEYLHNLAGLEHTSTTSVRLISSVTGEETTSGFGAEYWVDNLVSKVRFQAGLEEVCRKTSLISPQGGLTFIEIGPHNALSGPLRQTMSSLPLSAGYSSVSVLARNKDARYTLLETLGKLFESGSSADINFANLLFEPNLHQKLLSNLPSYSWDHSATHWRESRLSKAHRFRRYPCHDLLGSRIPSSTSLNPAWLHNIGVDQLPWLQEHVIDGFTIFPASGYISMAIEAMLQSSFEKHGSVNISSYDFKDIKFPAALLIPESPACVEIQLSLNTMDSLQDNQKSKWEEFKIVSVSVQGISIEHCHGSIRVHFSTELDEVEGAREQELNSSTQVTRLSELKSICNEELDHSDIYQDLNSRGNSYGPNFACINKISLGSTNAVSVMSIPDTSQCMPARFQQPHVIHPATLDALMHHPVTIFNRNTKASSFMIFGVDELKISSRLASEPGTTLTLATTTAGCGSRFPSADVSAFQTGSDPCLGPVIQMKNLKLRATGDSGENMPDKDWERNISYTMKWDLDADHMTSSMFIPGPDSFEADRAQEHKLELLNQAAAIYVRSCVEKLTVNGPPKLPGQFPHLFKWMIRFQQSEECKSLLQGVSDTNVESILKNAQKLEVEGELLGRVGPELEAILTGKADALSLMTENNLLYRLYSDDASSRCYSHVHAYVKQLVFKNPNMRVIELGGGTGSTTSPLLEALSPNGVLPFEKYVFTDVSSGFFERTRERLKKWDSGIEYKKLDLNGTLSEQGFEEESYDLVIAGNCLHVASSMDNVMSSVRKLLEPGGRVVMIETTRVVPFYNTFLGVFDGWWAGIEDGRVDSPTLTVGEWHSTLLRNGFNGVEIVANDFEGKAQRSAMMVSKASERTEAVGIKANTTLELVLGSSWTAQAPQFAFDLSSGFSQHGFHVNIEGLLTAQFRDDIIYVVLDNGSDPILTTDASPVFEQVKGLLTSANRVLWISIQENPLANLNPEKGMIAGLARVSRAENPFLHIVTMDVQESVFENPDRFIRTIREVVNNDFLHTSNSSTKELEYVYRNQEVLIPRLVPSARLNDRVQQASGRVTLKTQLFHQPNRPLKLHVQKPGFLDSLSFIEDNFFEEPLPASHIEVQVETCGLNFKDVLIALGQINKQIPMAAEFAGVITKVGSQHQKLFQIGDRVCGIGGSPYASTIRLHGHRASHIPPSMSFEVAASIPVVFATAYHALVDIANLQQGRTILIHSAAGGVGQAALQIAQNIGAEVFATVGNAAKRQLLVKEFGIPEDHIFSSKLRTFKKGIHRLTNGKGVDVVLNSLSGEALQESWASIARFGVFVELGKADSLSNTGLTMAPFDRNVTFASVDLQLICDYRPEKVASLLKTVLNLFSAGTYHQINPITMIPITNIEEAFRIVQAREHVGKIILGADSNTLVKAPAVTADDLIISQNGTYLITGGLGGLGLEIARMIATHGARHIALLSRRHLSPSELLALEEEFEPLGAKVIVLTCDITDLSKLRDSVSSFGSDMPPVKGVIQASMVLRDCVLSKMDLEDFKLATAPKCTGTANLVKALDGQPLEFFLMLASIASVVGSISQANYAAGNNYMDTLAQNNSVAKGPTTHFVSVDFGPIDDAGTIANSQRTKDGLIRQGYILLKLKQVLALISYAISHSVREDKDNQIILGVDHKSIVESDNEYTLKNPMFSHLSKVQGGQSVKEDGSPVQTIENLIASTQNTAKIELLISEEIARKISKLVAMDYEEIELERRMAEFGLDSLVSIELKNWITQTFRAKLQASEIADAAHILALGSIVASRSALISNEPPRMKSGNGDHSTNLSQNTTVAVKGNSGNTITLPKQPLPDLDHSLDQFLDCLLPVFTPEEYTLYEGYVNDLRKPGGFGRKLQARLSQLFHDPQVDNYLSGFYAPNMFLKNRQALVPWLNFFATHFMSPVPHTAAQRAAIISASAFQFKQKLESGEVGHEYLNEQPISTEAYKWYFNATREPGNPKDAMKKYPGNDYLVAFRRGHAYKISLRNGDEATSYNTLLDAFQRILDADEKPESWVGVLTQDTRDHWADTRTELQNLSKENRAWIHDIEASAFVVYLDEARPQIASERGPHFLHANGRNRWSDKTTQFAICDNGISATIGEHTMFDGYTFIRLYDFVCNAILTFNPQDPPRPLRKLPSAINMSEGYTFTTSPAIEKHITRIRAQLTSDAAKFEYRAFEIPTINRSLFSLHKCPPNSGVQLAIQLAARRYWGHNPISLEPISQNHFRNGRIDLNLTVRPPIAAFCAAAADRQPPTAELRKLFFDAARTHASNVATVSRGHSFDRHFFALQSVVRDGETMPALFTDTLYRSRKLPPQLMTNCLAAGALEGGDVFDHPNGMSINFQTEEKFVKVSIWGRVGTVDEFREILEESARDIKAIVDM

>Phain_OT5_Proseq7825

MQRKISTKPILKKIGIICDNCWQEQTEYACGHLKQVVTPLQMCKAHRKLDSYFKDYQLRTATLDQNCAPCEKKHEKEEWGRKEKEMEGLGREQKDAHEKREREHRVEVEERLKEVQRLKSLSDEDFERELKDVEERVIGKKSRARETASPRSFWEAKDIENLPGL

>Phain_OT5_Proseq7993

MPANQRQQSKQSPASPTSRPTAKYTNKDGSKFITVPKVFNPSDSETSPAMAQTTTKPNGQAPSPPTNPSGAVPSVNRKKQKRRDKQAAKLAAEQPTRIHTNGAHAPGKAHFHEHGLDARYEDNGQFDPADGEQDYSDEDGDGYSGSYGNDGSSANGHAIPTSDPSGKKSRKKKKSRAQPGTLHHGHSHDGTNGISHNDVSLPLPPLSASDMPRGPGISKEKIWNTSSQEERQRIKDFWLSLGNDERKSLVKVEKDAVLKKMKEQQKHSCSCTVCGRKRTAIEEELEVLYDAYYEELEQYANHQGDGGPTPMMPPPRRFGALGGLQPPNRLPPTFNGQQPSRGRIVEQLGDDEDEEGSEEYSEDDGDEDDYSDEEPEEIPRTNNAPDFFSFGNSLTVQGGILTVADDLLKNDGKKFIEMMEQLAERRMAREEDAREYASQSYGHPPNSAMHPHVHPHPHAHNHPPSQEDEEYDEDDEEDEYDSQDEDYDDEEEMVGDRPCGCEACSNLMQDTMTEEQRMEEGRRMFQIFAARMFEQRVLTAYKEKVAKERQQKLLEELEEENQADSQKKAKKARDAQKKKEKLAEKKKAHAEEKAKLDAKKAAEEAAIRAAEEKKAEEQRLKIEERRRKKEAQKKAEEEERLRKEAEKARRLQEQRDRQAELERKQREAKEKERKEKEEIRQREREAKEAKERDSRERKEKQERDKREKEAKAKTEKESREKQKREELAAQQAAAQAAVAATQSSKRPQPPVPIPSSLQSHAVSVASPHIPVATPAIPKAPTPIKLRTSSQQGSTGTGPQTPQGNGLSQNTSPHSSTPMQGSPGPIGPPGKNQGQQPFLHHPQATSPIHAALKSPPGIPPNTFAGMQPMGLGFQPGVPMMAPSFGGRMQHEPMFAHQPPMGGQFRPMAGPNGMPMHPQFVMPQMQQGRGFPLQHGPPGFPPQMQNGLGGIGQPIGAQKDGNPSQSHSRQQSGSYEKPFETPSSISQAQPIARPGPIGRPSSVVHGHHQGNPSLSDVDELSNRLGSRALLDESDEPLDAGVNGRRTNGMPNLNTRPGFSHFGMDPGFPSPMSSFNTWGAPQQNPFGPSSLPGSNYNGWGPSANGTFGSLGGSSAIRPPQSRPVAVRNLICRACHNLEGSTPDGFYDINLIKEQIEHLNSLNEQPVSEQELLEMCETEGNSMNGGGSFEVRTDGNGQRSIRYENDIPSAHRPIGAPGEIGSPIVGGGSMARFAHGPPPGF

>Phain_OT5_Proseq8148

MGVLTGLRYWLQAMVYMISPIPEAKSLKSPNPSSPMPASIFKPLLHPREKETTAEVDGYFLKHWNFENEKARKKFVAAGFSRVTCFYFPEALDDRIHFACRLLAVLFLIDDVLEKMSFDEGSAYNERLIPISRGDVLPDRTIPVEYITYDLWESMRQHDKEMANDVLEPVFVFMRAQTDRTRIKPMGLGSYFEYREADVGKALLSALMRFSMGLKTTPDEIALARPVEMNCSKHISVINDVYSYEKELLAEKEGHAEGGALCSSVGILAKEADVDIAASKRLLVYMCREWELCHEQLVAKIRAQPNGRSENLRRYMKGLEYQMGGNETWSQTTKRYSMVS

>Phain_OT5_Proseq8153

MSPYGASANNEGNHRAIDPVTMPIAIIGISCRFSGDATSPEKLWEMVAEKRSAWSEIPSNRFNQESFYHPVGGHLSTMNVKGGHFMSEDIALFDASFFNFTTELASSMDPQYRLQLESAFEALENAGVSIEKLAKSNTSVFTATFQRDYHDALMRDPETLPRSFLTGNGMAMMSARLSHFFDLRGSSVTVDTGCSGGLTALHLACQSIRTGEASMALVGGTSIMLNPDMFIILSSLGMVSEDGISHSFDAEPGGYGRGEGIATLLLKPLEDAVRDGDLVRAVIRETALNQDGKTSTITSPSQEAQEELIRACYNRSGLDPRDTTYAEAHGTGTKTGDPIEAGAIGAVLGRSRPANKPLYIGSVKSNLGHLEAASGLASIIKMILAFEKGCLPPSIHFRSPNPKIQFDKLNLKVVTELIAWPEDSLKRVSINNFGYGGSNAHVILEAAEQQNPTQKSTNGITNGICSESKQSMVFILSAKDKNSLKTMASDLSGFLEAKTPYKNKLRMEDLAYTLGQRRSRFAWRLAASAKSLPGLIKAFSEDGTTPQHSSGAPRLGFVFNGQGAQWHAMGRELIAVYPVFKQCMLEAEQHLTDFGAKWSLIEELSRDAKSTRVNEAQYSLPMSCSVQLALVRLLASWGIKATAVTGHSSGEVSGAFAAGALNLRQALAIVYHRGVLNNVLIHTSKLRGAMMAVGLGPDDLHKYTLSGPAGKVVNACTNSPTSVTLSGDLEAIELLEETLLADKVFARRLKVETAYHSHHMVPLAKDYLSALDKDVVELGDFNGVLYYSPVTGEFVKTAQELGPEHWVSNMVKPVLFLDSFRNMCIESPASESLEPQTNIDVVVEIGPHGALAGPIRQILTDPKLKGFGITYASCLTRNEDAVHTMQSLASLLLSKGYPVDIAAVNFPRPTSGLKVLDLPKYPWNHSSRFWMEPRVNKAWRQRKRPGHDLLGSLVPGSTSLAPTWKHVIRIDEIPWVRDHMVGSDIIYPGAGFIVMAIEAMRELHLQEQRIVDGYYLRSVEIMKALVIPDTLEGVEVQLSFQPSDSRELDPSWREFHIYSMTTDNECIKHSSGSISTKYNHADNLAGLSRRQMKSTIDFDPDTSTRTTKVDTKDFFQSFAALGIKHGPSFQNLTAINVGQGESEAAFEIADTASLMPGGTQHAHVLHPITLDAIFQASYSTLPVADLQRMGAAIPRSIKSMFISSNIGAEAHHQFQTFQSLNDHSSQGFNVSMKLISKEDTSHTPVLEIDELHCRSVGKVVSQEIDSKNKNDSVVIQWEPSIEFSDHLGLQNLLRMSADSSEAILIANLKRATFHLINDALDRLTLDESDGLVGHQKLFFNWMKLQKTKAEMNELAPDSCSWLKTSEGVKQMLYGNVSLASVNGEMLVRIGENLSEILTNEVAPLELMLEDKLLYSYYEKAVHYDRSLEQVHKLVKLFGHYNPKGKILEIGGGTGSCTGKVLDALSDSRPGYDPRFAHYDFTDISSGFFEVAREKFDRWRDQMGFKKLDIEQDPSKQSFELGSYDLIIACQVLHATKAMNTTMTHVRQLLKPGGKLIMMETTQEAIDAQLVFGTLPGWWLSEEKERKHSPNLTVPHWDKVLKRCGFSGLDVEARDCEDDDNYAMSVIMSTAKRDDSPRFPEQIALVYADALPPQPWLEQLKVLLARLTSTNVTVERLDRLNPSGKVCIFLSEMEHAFLSKMDETRFEKIKALLTRSQGVFWITRGAALECKKPSVALQTGLLRTLRLEDTGRQYITLDIDSESEPWTASTTSAILDVFRFTFDLSIGKSVVDCEYALRGSGILVPRMYSDVAENYSIPAAELMDTKLEPFYQSDREIRLDVAVPGLLDSLAFIDAGSTDETLPDDFVEIRPEAFGLNFRDLMVSMGQLKEKVMGFECSGRITRLGPNPSHGLQINDRICALTHNGHYSNFVRVHSTGVARIPDDMTFEVAASIPMIFITAYHALVDTARLESGETVLIHAAAGGVGQAAIMIAKWIGAKIFVTVGSNEKRDFLTKTYGIPPNHMFSSRDNSFAAGIMAATDFKGVDVLLNSLSGELLQEGWNIMAYHGRLVEIGKRDIQLNKNLEMLPFHRAISFSAIDLIHLGNYKNRVVSRVLASVLELFSNHDIQPVQPISVLPISEIQRGFRILQAGKQFGKIVIKPQLGDLVQVLPTRKVLRLSPNASYLIAGGMGGLGRSIAQWLVRHGAKTLIMVSRSAASQNDAELYLNRLKAQGCRIILKNCNIANEGDLSGVLSECALKFPPIKGVIQAAMVLQDSVFEHMNFQQWQKAVHPKVAGTYNLHRQLGVELDFFVMLSSSAGVVGNTSQANYAAGGTFEDAFARYRASQGLPAVSLDLGMVRSVGYVSETKGVAERLAKLGWRPLGEQEVFHILETAMLHPRRDPRSSQIITGLEALENDDDIVWRREPRFWTPKHQNNQRHNSTAGHRSNDAITLQDGLSNADSSETAVALVTAAVISKLSAMFMLPNDEIKDSAPLSHYGVDSLVAVELRNWLNTHADCDISIFDVLQSVSLKALASKVVERSKHVVNVKS

>Phain_OT5_Proseq8468

MASKPKQKTAIVVGAGVGGIATAARLASAGFSVTVLEKNTFTGGRCSLLTHEGYRFDQGPSLLLLPGLFAETFADLNTSLSAEGVELLKCDPNYNMWFGDGEKFELSSDTAVMKREIEKWEGKDGFERYLSWLAEAHRHYEISVREVLHKNFTSIFNLLRPSFLGHLFALHPFESIYSRASKYFYTERLRRVFTFGSMYMGMSPFDAPGTYSLLQYTELAEGIWYPVGGFHAVVAALVRICERLGVDIWLSTPVSRILKSPDGKTATGVLTEAGDTLTADVVIINADLVYAYNNLLAPTPYSRSLQKRQGSCSSISFYWSLSRKVPELSTHNIFLADEYRESFDAIFDRQDIPREPSFYVNVPSRVDPSAAPEGKDSIVVLVPVGHLLNSSSSTTFTSSKGLPKEELNWPALIASTRASILSVIQSRTHCDALAPLITHEMVNDPITWEDTFNLDKGSILGLSHSFFNVLAFRPSTRARGLQGCYFVGASTHPGTGVPIVLAGAKVTTEQILRDLGMEIPWTGMGGVEGKGKEKGDMDRVRRNGGLISDLGIWVLAIVVLVVSVLYVNSGLVVTWDWDRKRGWGSGRLEGAGLGMR

>Phain_OT5_Proseq8537

MLSLRIQRSFRLVFAAIFLTGCLDQASAASIEYCSGLNTADSNKNSSIYQSNGLCSNFCKSSYAFAVVQDDGCWCSNYVPGSTTSGCTQTCPGYPLELCGGSGVYGYIALGNTPSGTKGGSSAAASSTSTSPSSTTPQVTVTATPSAVTIVDTLTTGPISQSSSTSTSVTSTTTQPATTPTPTLATTSPSSTSTSSSSSSSMWTPTPVTSLETVTGQVHTVTITPTSPPNSALVAPVSKNKSSSGISTGGAVGLTIGLVALVALISAIVYFCLKKRRKDNEAERLNPDTSRHGSSAGLGGPIPSRTMSENSRYVLGTNGREVVETWEDSAPGSRKSRLVPVDPRLDPFSPVYQRASDNKSRESVNTIRDDHDYSRRVHQQGPILRATNPDT

>Phain_OT5_Proseq8549

MPGELSEGTCFVALTKDHLDAKSVMDRVRSPKAGAIVLFAGTTRDNFAGKPVKELQYTSYEPRALQSMLAICKSVKEKHSLTAISMMHRLGIVPIGEESILIAVSSPHRQAAWRAGEEALEECKEKVEVWKKEEFGGKEGGIWRANRDGAIGVPVDGDEADEKEVVQGPHGPVIRPSRPGEKGHGPALKASLIRRLHHGRTVRLRKCGVVWAKTTSRNLSQQFAQVDGNERVAGG

>Phain_OT5_Proseq8679

MAYIARSYRPRVALASESPYDSDFQVLDIIDQDHTIKVYNIYNEKHQLPDSDTRTLARCLWQLIPPLDSVFCGDFNAYNPWWDPLYEARDIEGIALADWIDYHDLALLNTPDWAIVEDIGSDHLGIVFTIQGSRIGLADPQATRFDIKRADWTKFGEKLFCQLSPLQDSLYQLSYSLSRNYSLKSLEQIDAKTSYKVALDQLDDMAERYTSAILCAAHDSIPQIRLAPNAKPWWNDSLKTLRRDMNHARLASIRAFSDQSHKRYTSARNIYFQAIKTAKKDHWNQFLEKEDSKSIFKALAYTKPRRSELVPNLKLSATEFATTFTAKCNLFRQTLFPPPPETPSLN

>Phain_OT5_Proseq8681

MARPTNIDLTLAHRSIANHIQDWAVVEDIGSDHLGIVFTIQGSRIGLADPQPPDSTQNAQTGPNLLKKLRANRRKTSYKVALDQLDDMAERYTSAILCAAHDCIPQIRLAPNAKPWWNDSLKTLRRNMNHARLASIRAFSDQSHRRYTSARNIYFQAIKTAKKDHWNQFLEKEDSKSIFKALAYTKPRRLELVLNLKLSATQYATTFTAKCDLFRQTLFPPPPETPSPNWSSYTASSAWKWPKLALEEVKLACSSEIKSTSPGPDYITQTIITHAYSACPSQISLLFETLVNTGYHLKCWRQANCAILRKNNKPDYKQPSAYRPISLLSCLGKVSERILAKRLAYLAETTPNLLHPSQIGGRLKKSAINAGAMLADYVYSNRQKKKITTTLLLDVKGAYDHVSKNRLLIVLRDLALPDSLASENAIQFDPKKLELIHFHTGEAAKLAPLILPDGTIVPLKQTVKWLGIHFDALLRFKEHITIRTALAKSAFGRTFHTSPIQAMEVEASLSPPAIRLDAATRKYAFRLAKLSPDHPVNRQISDASDASDSSDSSDSSDFSSFSSPPKYGLQRIASTIAPLLSSKTEPI

>Phain_OT5_Proseq8866

MARPTNIDLTLAHRSIANHIQDWAVVEDIGSDHLGIVFTIQGSRIGLADPQPPDSTQNAQTGPNLLKKLRANRRKTSYKVALDQLDDMAERYTSAILCAAHDCIPQIRLAPNAKPWWNDSLKTLRRNMNHARLASIRAFSDQSHRRYTSARNIYFQAIKTAKKDHWNQFLEKEDSKSIFKALAYTKPRRLELVLNLKLSATQYATTFTAKCDLFRQTLFPPPPETPSPNWSSYTASSAWKWPKLALEEVKLACSSEIKSTSPGPDYITQTIITHAYSACPSQISLLFETLVNTGYHLKCWRQANCAILRKNNKPDYKQPSAYRPISLLSCLGKVSERILAKRLAYLAETTPNLLHPSQIGGRLKKSAINAGAMLADYVYSNRQKKKITTTLLLDVKGAYDHVSKNRLLIVLRDLALPDSLASENAIQFDPKKLELIHFHTGEAAKLAPLILPDGTIVPLKQTVKWLGIHFDALLRFKEHITIRTALAKSAFGRTFHTSPIQAMEVEASLSPPAIRLDAATRKYAFRLAKLSPDHPVNRQISDASDASDSSDSSDSSDFSSFSSPPKYGLQRIASTIAPLLSSKTEPI

>Phain_OT5_Proseq9011

MYLSSILLFSSLHILRTNAQFNFGVLGASAITNTGPTIINGDVGLYPGTSITGLTAAQVMNGVIHNDDAVAMQGQAEALTTYNMLAGLTSTDDLTGQDLGGLTLVGGTYTFTSSAQLTGTLTLDGGGTTDSRWVFQIGSGLNTATASSVLLTNGAQPCNVFWQVGTSATIGTTTSFQGNIFAMASITFATGATNSGGLFALNQAVTLDTNTINTSNCTVVSSNGTAVSSSSSTSSGGGNGGSGGTAGSGSAAGVSTSPPTTSGGGGNGGSGGTAGSGSAAGMSTTLPTTSGGGGNGGSGGTASSGGAGGTMTGNITNTSLTGSSLSTLSSAAGLSTTSISANSTITNTTLTSKTTASSLNTSSTAVGFSTTSISANSTITNTTLTSKTTASSSNTSSTAVGFSTTSISANSSITNTTLTSKTTASSSNTSSSAGFSTTSTSANVTIPTTFTTPTTFKNISSSGGNSRSGGTTSSGGGTGTITNTPLTGKTTSSSGSNSSNAVVSAENSGTRTGNTGASSNTSTGVQNVSAVETSTTQRFNSQISSTRWMNSTNLNRINVINMISTYASTSTTTFTVECTSVPTTIQTYLQTFTVYDVGVTTITCEVPKPTSVIVECTSAPSTVWTYDQTFTVTEIGITTIYCDIPISYQAITWDVPKPTSVIVDCTSAPSTVWTYDQTFTVTEIGITTIYCDIPISYQAITWDVPKPTSVIVDCTSAPSTVWTYDQTFTVTEIGITTIYCEIPIPYQAMTTTSTCGCTETITETITETIIERVPRYCGT

>Phain_OT5_Proseq9013

MEHQQNQPQPPPQGGVPGPTGRRLHIAHRRSPSELTPLMSMFSSPGMEQLAIQQQIELLQQQQQQIQATHQQYVNMGMIPPTQHLAPGGGYNPIQPQMQNLSPQSAYQFPNQMQQQQPQMNAPMGAPTQPLSHRRNQSALPNMGMGPPPAPSSGAAGASFGDFGGHNRDLSSVRGGRGGGPPGGGHQRRHSLALPEAKKAAELAQQKRTTSGFQFPIPGTTGGTPTSAERTESPSGEDKNTAQSPSVGNHVVSGANLRARGSAHGRSQSMAVGANNRGGSSMRGGGSFQFPPMQATPDAGASAQGQVQGQGNDFQRRGSSTGHNRSSSRNFEGNWRNQPPQGQVPQDQQPPMGNFQGQGQPQGGFQPGHRNRPSMNQSINNIGNFQYGGQPPIPGLPQAQMALIPQHMYPGQTLNPLQMHQLQAQIQATQMNGHVGLQGSQHAPQLSAQQQQQQRKTLFTPYLPQATLPALLGDGQLVSGILRVNKKNRSDAYVTTQDGLLDADIFICGSKDRNRALEGDLVAVELLDVDEVWGQKREKEEKKKRKDITDTRGGGSTNGNNNQSHRDNSTNGDDQPSTGEGTIRRRGSLRQRPTQKKNDDVEVEGQSLLLVEEEEVNDEQKPLYAGHIVAVVERVAGQMFSGTLGLLRPSSQATKEKQEAERQARDGNNNRHQESRQQDKPKIVWFKPTDKRVPLIAIPTEQAPRDFVEKHMDYADRIFVACIKRWPITSLHPFGTLVEQLGKMGELKVETDALLRDNNFASDEFSDAVTRSVGLDDWSLAKEDETAIAARRDFREEKTFTIDPNGANELDDAIHVKTQDDGKFEIGIHIADVAHFIKANSLVDREAKKRGTAVYLMNRACAMLPPKISTEICSLTPGQERLTVSVVFKVNATTGTVSDDETWVGKSIIKSSGKLSYKDVDAILAGYTDTKLEGAEVKDIQILHAVAQKFREQRLGTDGETIAPLRLIYQLDDENVPVEHNIFDSTSSHELIEELMHKANAYVAQKISRGLPEKALLRRQGPPNPRRLQTFADRMNKIGYEIDTTSSGTLQNSLFKVDDTNIRKGMETLLVKTMHRARYFIAGKTPPHLYPHYSLNVPLYTHFTNPSRRYADLVVHRQLEAVLSEGKIEYTEDLETLVKTTESCNTKKDSSQNAQEQSVHIESCRIMDKKREDAGGELISEGIVICVYDSAFDVLIPEYGFEKRVHCDQLPLKKAEFRKNDRILELYWEKGVPSSAYVPEDERPRAGASQRMTNAAAAAKQAAVAERAKKEHEEAQRKQMETGTISTDDVDALFDDEDDASDVAESLAGVSLAERPTQSVPPSPTKNSLTATGNLHRTRSESKVPTSESIDAKLSLKEKYLKLFTLREENGEYIQDVKEMTRVPVILKTDLTKSPPCLTIRSLNPYAL

>Phain_OT5_Proseq9054

MSATNGAELNGNGFHSSSSDGSNTPPETNGTTNGHTNGYTNGFSKIPEAGVEPIAIIGMGMRLPGGVHDSESFWKMLIEKREARCRVPTDRYNVDAFYNKTKKPQSVATDSGYFLEDINLDDFDSGFFNLGKKELDRLDPQQRLLMEVVWETLENSGETNWRGENVGCYIGVFGEDWLETQLRDTQEMGSYRITGHGDYVLANRVSYEFDFKGPSLVIKTGCSSSLIGLHMACDALRNGDCTSAVVAGTNLIMTPTMTIAMTEQGVVSPTGTCKSFDASADGYARGEAVNCVYIKKLSDAIRDGDPIRGVVRSTAINCDGKTPGISYPNTDAHETLMRRAYKVAGIDNFSETAMVECHGTGTPIGDPVETNAVARVFGKDGVIIGAVKPNVGHSEGASGLTSTFKMLLAMEKLTIPPNMRFKNGNPKISFEAAKLTVPLEPMAWPAGRKERVSVNSFGIGGSNAHVVIDSAAAWNVGRPKSLASNALARPELLVFSANHQESLKRITQNYEKFIEEKSANLSDLAYTLALRRTHMQYRSFTVAGLDEPLTFAPPIRPGNQRVLVFVFTGQGAQWAGMGKELLTDYPSFQDDIRAMDKTLAHYELLKPQATSLINRPEMSQPLCSAVQIALVNLFRSWDVKPQAVVGHSSGEIAGAYAAEAITADEAILMAYYRGQVMKLQGLAGGMAAIGLGRESVTPFLVPGVVIACENSPESVTLSGDADKLLEVMDTIGQANPDKLVRRLKVDMAYHSHHMAAIGEQYQALIQDKVSSRKPTVPFFSSVTGKAIRQAGKLDAAYWRSNLESPVLFSTAVKSILGPAVQNSLFIEIGPHSALAGPLRQIFSTATGSHAYLSTLQRNNNGTKTFLSAIGQLHCHGTKLDFSIMNPSRTVLPDLPLYPWHYERKFWNESRLSKAYRHLKFPHHDVLGSRVAEVNDFEPTWRNILKLDNVPWIADHKISTDIVFPAAGYLSMVGEAIRQLTTSEDYTVRNVTLTAAMVLEDSKDTEVMTSLRPSRLTVSLDSAWYDFTVTSYNGAAWTKHCVGQVRSGFETDLVDTTKLDKLPREVSTHHWYAAMRKVSLNYGPAFSGLDNISAGVNEHTAVADVKNWEREDESPYSLHPATLDPCFQLVSAALAYGQARKLNQMCLPTSIDLLSIRKSVSDVRMFAETVSSRKGVVIGDAFGATNGEPVMRLRGLRLSPVENDSGNDPDPHAAIQLEWKPDIDFLQNKDLIRTVKSVRETHKLGEKLTALCILETSSRVSGFKSEQPHLNQFIAWIAKQVSRIEAGDYALVDDAKDLAKLDQKARMSLIDETSTALKATDSPAAGTALARIVENIQDIIDGNVDPLELLMKDDILTEIYNFAGQWDYTDFFSSLTHKKPHLKILEIGAGTGGTTDIILKNLSSTFGERMYAKYTFTDISAGFFGAAKERFHNYAAIDYAVLDVSKDPIAQGFEANSYDLIVATNVIHATPVLQESLSNIQKLLQPNGQLFLQELCPDSKWVNYIMGLFQGWWLGEADNRFEQPFVSPQRWDEELKKSGFSGAETVLYDDEKPYQNNANILARPVIVDKSTKSVTLLSAKSTDSAVTELETILKDRGFKVDVIEYPQLPPAKQDIIAVVDLAEPFFTDIEAEKLKSFQDYNLHLQNENIGMLWVTQATQILCKDPRYAQAIGIARTARSELGVSLATLELDTVSTDNWGRVADVFSKFQRRTKEEEVDSDMEFAVAENIIYTSRFHWISVNKELSQTAASKMPKRLEVEKKGMIETLHWAQFPEPELKSDEVIVETHAIGMNFKDVLISMGIVDAQRSDSSGLGCECAGFIRKVGSAVTRLAPGDRVVVQATGTLETRVKANSKLCVKIPNSLSFEDAATMPVVYGTVVHSVMDLGHLEKDQTILIHSAAGGVGIAAIQIAQYIGAEIYLTVGNEDKVKYLMDTYGIARDHIFHSRDASFLPDILRATNGKGVDMVLNSLSGDLLHASWQCVAQYGKMLEIGKRDFIGQGKLGMSLFEANRSFFGIDLAVMAEERPDVIHKLLNKVLQLYIRGAIKPVRPIKVFEAAQIKDAFRYMQQGQHMGKIVLKMPLNPEELTGTSTTKQLTLRPDVSYLMIGGLGGLGQAISTWMAESGAKNLIFLSRSAGKAEAHKSYFKELETMGCSVQAFAGSVSVLADVENVVKNATMPIGGVMQMSMVLRDQAFARMSIDEWNAAVGPKVDGTWNLHKALEKETLDFLILFSSFSGLVGQWGQVNYNSGNTFLDAFVQYRHGLGLPASVLDIGCVVDAGYVSENQSVLDTLLSTSLHGLHEQDVLDSLQMMMGRSAPAPASPAKTYTNPGQVGIGLRSTMPLAEPGNRSIWKRDPRMAVYRNLEAASSSSAASDNEPLRLFLVDAHAKPAMLTEKASAEFLAREIGLRLFISLMKPEEDLDITLAPTALGLDSLIAIDLRNWWRESFGFPVTVLEMMNAKSIVELGELAAKKLLEKLQPSEDKPSDP

>Phain_OT5_Proseq9060

MASATSFYDFKPLDKKGQPVPLENYKGKVLLIVNTASKCGFTPQYEGLEKLYKSVKEKHGDDFEILGFPCNQFGGQEPGSDEDIQSFCQINYGVSFPIMGKTDVNGDKANPLFEWLKEEKPGIIGIKRVKWNFEKWLIGKDGKVKGRWASTTKPESLEKAIEAELKTGPAATTGELKGEEVKADL

>Phain_OT5_Proseq9131

MVSFSSLVLVASAAIAGIYAAPHTSAGEVLAPRDGTPSSTGTNNGFYYSFWTDGAGDVTYSNGAAGTYTVTWSGDAGNFVAGKGWNPGAARTINFSGSYNPTGNSYLSIYGWTTNPLIEYYIVESFGTYDPSSAATQSGSVTADGSTYKILETTRTNEPSIEGTATFQQYWSVRANHRTSGSVNVTAHFEAWAALGMTLGTHNYQILATEGYHSSGTATMTVS

>Phain_OT5_Proseq9152

MVADDKPASTFKYSEKPLYTTSNGCPVENPEAFQRIGTNGPLLLQDFHLIDLLAHFDRERIPERVVHAKGAGAYGEFEVTHDISDITSIDMLSSVGKKTPCVARFSTVGGEKGSPDTARDPRGFSIKFYTEEGNWDWVYNNTPVFFLRDPTKFPLFIHTQKRNPQTNLKDATMFWDYLSTHQEAVHQVMHLFSDRGTPYSFRHMNGYSGHTQKFTKPDGTFVYVQIHLKTDQGNKTFTNEEAGKMAAENPDWNTQDLFDSIQKGEYPSWTVYAQVLTPEQAEKFRWNIFDLTKVWPQKDVPLRPFGKFTLNKNVENYFAEIEQVAFSPSHLVPGIEPSADPVLQSRLFSYPDTQRHRLGVNYQQIPVNAPIRAFNPFHRDGAMAVNGNYGANPNYPSSYRSMTYKPVKPTNDHEKWAGAAVMDLSEVVAEDYVQAKGLWDVLGKQPGQQDNFVGNVSGHLSAAKEDTRKRTYEMFGRVDEQLGKRIAEATEKLAPAPGSQAAGSAQSHL

>Phain_OT5_Proseq9413

MFQPILDLSLVSLFLLLSGFYGTILIIQFILDPLRDIPGPLLARFTRFWFFFAIYKGSFEKTNIELHRKYGPIVRIAPGEYSIDDYEAARTIYGYGNAFVKAPWYSAWVPPSPELQSLFTERDPHRHATQRRKFSAVYSMSSLVGYEPFVNNCSSLLSQRLSEFAKLGETINLHHYLQCFAFDVIGEITFGNRFGFLDTGEDKEGVFAAIDARGVYGTFVGIFPWIHRFLYPLLPNTGGFRYVLNYTLRQIETRSKALKDPLNVSREGPPDIMTKVLLAHEENPQKMTRADLIIICGSNIGAGSDTTAITLSSIFYHLMKNPQSYHRLQSEMDVAASEGRISDFVTFKEAQDLPYLQAVIKEALRMHPATGLPLPRVVSPKGATIAGRFIPGNAHVGINAWVASRNTSVFGPDADTWRPERWLEIEEQGRGGEIEKYFFPFGMGSRACIGRNISLLEISKLVPQLLRNFDFVLDESEELRSLNRQMVKQQNFKARIVARSEKS

>Phain_OT5_Proseq9515

MAIRKTQNGVHPAEGVLEDLNDGPSNTSTNGFTHNIEHPTVEPIAVIGMSLKFPQEATSPEAFWDMLYKGRCAMTEFPPDRFNIDAFYDKENSGTGTLPLRGGHFLKEDLGAFDAPFFSVTPAEAVAMDPQQRIMLETSYHALENAGLTIDKCSGSKTSVYTATFTDDYKSMLQQDSEQLPKYAATGLSGSMLANRVSWFFNLRGPSMNLDSACSSSLSALHIACQDLLNGTSKMALVGGCNLVFHPDFMLIMSNMSFMSPDSRCWSFDHRANGYARGDGFGVVVLKKLSDAMQDGDTIRAVIRATGLNQDGRTTGGITQPNGEAQQLLINETFTRANLDMAPVRFFEAHGTGTALGDPTEAKAIGNSFRSYRSEEEPLIVGAVKSNIGHLEGGSGIAGLIKTVMILEKGIILPNTGLEKVNPRIDTQNLRIHFPTRPMQWPSLGLRRACVNSFGFGGSNAVVIVDDALHFLKAHRLRGHHRTIDLCLQKSNGETNGTKETNGFYEPVEQKSERDVGRVTDENGLSTKLTSTQKHPRPRLLIFSAADEIGIKSMLDTYGEFFKKRGRAVLAPSFDDIVHTLAAKRSLHAWRSYTVATSGSDLISLEKDSSKPIRFSGDPKSIAFVFTGQGAQYAKMGNGLLAFPLFRLRFAELDHILQDLGCEWSLQALLCDSTSTINEPEYSQTFSTALQIAIVDLLKSFGITPAAVVGHSSGEIAAAYCAGAFDDRTALKIAYHRGRLAARLPTLHKTPQAMLAVALSEERIQPFLARLTEHVEILNVQVGCVNSPKSITLTGDKDQLEILESWLKDDQHLARKLRVNVAYHSPFMKAIKTDYYESLKDMDSKVAKKVTIPMISSVTGAVIPLGRVCDAEYWVKNLISQVKFSQAVSLLSVQSGKAPRKTLGSKSQSLSGIKELVEIGPHSTLQGPLQEILTNNKSQGRMSYLSILNRQRDAAACTLDAVGRLWSLGHHIDMLAVNGFDKKTRAVMTDLPSYPFNHTQKYWFEDRTSTQFRFRSHARHELLGSLIPSSSSFEVRWRNFLSLEKLQWVQHHEISNNCLLPGAAMLAMAIEGVKQLTTREAKVVAFEMRDVQFLNALQVPDSSEGLEIQVSLATSKDRAIEHEPWYEFRLFSYSSEWSEHCRCRIRAETASQVWSISSPTSSYHLELSSIANACVHEADAKHFYDVTRDNGVNYGPIFQTLNHVRFNESGAAMADIRAFSGSADDKSKRLNFDAYTIHPSVLDGLFQMVFPALNQGGALDLPTMVPSSIRRLVIYANDDFISSGSHLRASVRSTFNGYRGTESKVIAVSPSNYELFSIMEGYQTTFVSTSNDTQLLNSAQRPLLSHLEWRPDLTLLNNEQISELCKQARPQNNDFVNFQQRLSLLIHHYISTTLEALKQAPPEGISPHSLRYIRWMVQQHSQPDSDWAPRALSEPAGVDALEKEIVTANNLGQYYVTLGRNLLPLLLDEEGSKSLVSREELSSSYFNEQLASQNIRKQLEVLLSNLAHKTPTMKILELGGSGGNSTSPCLSILSAKGNTRWLRYDYTNVSSDKVLQAQEKLGSSSNRVHFQVLDIENEPAAQGIEEFSYDLVIAAHVLQMNSNLPRTFRNLRRLLKPGGSLILVGISAPQSVNIDFIFGLIQGWWTNGQTSLNPYRSKEEVSKALTDSGFNGIDYVIQDSEDQENHEIGVIVATAAEQIVSEALKLPEIAIVLDEKSLTKANFANRLKEKIQGDGKTTALIYNFADAVSKRSINGMFCIFLLDYDEPFFPSISSDEFNNFKEVLSLTNDILWVAKDTQTLCRPEFHMVDGLSRALRSENPKLRFARVAITDNDQTGLDGPTAVLTVLNHAIQSPLDDMEPEYEERDGMLCINRVIQSQRMNRLIGANIASYQERTLQLSHELPIRATMKNSGMINSITYEEVTELDDLPEPDEILVQVRAVGLTSRDYQIASGQLNEESIFTECSGLIVKAGSDSGFSPGDKVLVSCSGVCTTSLRCKASSAARFPDSMSFIEAAALPTSTLLSFQALVNAASLARDEVVLIHHAAGAIGQAAIQISQKIGAKIIVTTGSEEKAELLQSTYGLSRDSILSNKDPHLSQSILQATANHGVDVVLSFDLGDDLDVSLEALAPFGRLVNLGIGNNAVSAKVIQQVLPKCITYTSLNLAEMQRLRPQLIGKLFKQTSSLIHSGDIRPAMPLNVFQACDLVKGLQSFHTGRSVGKVVIDFDPGLSVTIRKALVTNKPSYYFDPEATYLIAGGFGGLGRSVSRWMVDRGARNLIILARSGTRTESAKELVGELEALGARVEAPACDIIQAASLKGVLEQCAKTLPPIRGCLQCTMVLRDAIFDNMTHDDFTTGTQPKVHGSWNLHTLLPKGLDFFVSLSSIGGILGATSQANYCAGNTYIDALARYRTSIGEKAVSIDLGMMVSEGVVAETEGMLDSLRRLGYFMDIRQAEFLALLDHYCNPALPLLPPSESQIIVGIEHPASMEAKGLEVPHWMMRPLFRHFQLINKDTAGPSNKQKQSAADSETVLRQSASAEIAVEHATGWIVSKLSQILGIKADEIEVQKPVHVNGINSLIAVELRNWFDKKLGADVTVFEILGNMSIVDLSKYTVDKSRFRDEK

>Phain_OT5_Proseq9569

MNNASPIAIVGLSYRAPGVGRKGLWEFLAEAKSAWSKVPADRFDQDAFYNPDSERAGCFSSQGAHFLPDDIYSFDAQFFNLRADEARAVDPQHRMMLECAFEAAESAGLSLIDLAGANIGVFAAIGSTEYTQQSSEDLFSTTTWTALGGAPCMFANRLSYFFDIHGPSISLDAACASSTYAIHMACQSLRAGECNAAFIGASSLIMGATQWNVLDKLGALSPEGKCFSYDTKASGFGRGEGGACLLAMRLEDALKSGHPIQAIIRNSAASHSGRSEGITMPSRVIQEALLLRVHEEVGLDPSETPVVEGHGTGTQAGDPIEAGAFATVLGKARTPSDPLYIGSLKSNFGHLEGASGILGVIKAIMMIQRGCILPNSGFEEFNHHIEGRDKLKVAQTIIPWPQNARKRVIVTNFGFGGSNAAVILEEVPAKQTNGSRLENGTANGGVNDTTKGIALTNGGTKNGTSNGTALINGNGAAPHDVSSREHLLFVLSAKSESTLASYLSSFIEYLNNAPQSTDFMEKLSFTLGQRRTQYPYRLTATANSATSLKTQLSASKISKVKDRIIAYVFTGQGAQHPQMTAGLRHHKTFAAAINQAELYLHEMGASWSLNEELDKPGFESRIDDAEISQPACTAVQLALVMLLKTWGVAPTGVTGHSSGEIAAAYAAGLISFRAAIAIAYFRGKAAAQLSLEHDQKGAMLALGTSFEAASKLLQQNPNGYATIAAINSPQSVTISGDESAIDNIRQIADTQGLFARKLKVEVAYHSNHMQLVAASYLESIKPFCHLESLSFDHDESRAVFVSSVTGHIEGADSIDASYWVKNLLQPVRFADAIESIFSTGDNKIDAGQRTAAVPNIIVEVGPHSALQNPIKQTVEVLRQRNDQGLTQFTYLASLVRGKRGYEALLGLAGSLFSMGSSIKLGMVNQLDHHNAHVLTDLPPYAWDKSVSYVHKSRIMQEKLHPGQSFSQLLGSKSLYGNGSEPTFRHVFTLDDIPWIRDHNVGGHVIFPMTGYLSMAIEALRRVSPKIPESILVREYHVKRSLDIEEGERIDITTKLRPVTTGTESFSTTAWVFEINSWTETNGWTAHCYGQVEPEASEMTMESPTFKVSAPLIGGENLKERDAKLIYSNLGQGGTLYGPSFQAMRKYWEGPSWTVLETELRDLDLSTPGPFGSPISIDPPTLDSHLQGIAPWQNNAAHMPNYVSRLRISNKIEAVKNQRFTIVTRLLGNDTKAGRLRSSIAVFLDSHGSLRALAEWESVTYRFISSSDTGDSASSLPAGYCWDLIPSMDFVDNEYAANMLTVESDTLEIARQHRRKVNRAGVYYMSRALKETAGEDLSHLPSHLLRYLNWSKKVVAGENLALDIEQTSLLTEVLTSDAQGEMLCAVGEKLVQILRGEMQPLEIMLKDSLLMRNYEQEAATTHSSRALGRFVRQLSDIKPDLRVLEIGGGTASATLPVLDELSRGEQDLPAFLSYTFTDISTGFFENARTKLAKWAKCITYKKLDVSQDPVQQGFATEQYDVVIASNVLHATPNIATTLDHVRSLLKPNGKLLLMEAIVHPSLSLPYSLLPGWWLSEDEYRDHEEGPLLSEEKWQTVLTARGFSGVDAAIADYPGTPEHVISVICSTRIGMREDSDDSRSITICGLIMDDEEEEFAQIVSDHVTQHLGCPSEVKPFAEMDAEDKRFCIFIESPRHSVLGDLSAENFEIMKNTLLETEGLLWVVPEGCPPEAESIKGLLRSLRHETESKNLLILEKTPCTSEGALAIAQIAGRLRDLELANSAGITDQDFVWHNGMIHLPRLRQLKEATDVFASEAGIAVRKMQNVWQNERSLEMTVDAAGSPDSIYFRENEVKTKPLGDDEVLIRVVAAGVNFRDLLLVLGSIPWTRPGFEGSGVVTQTGSGVGDLKPGDRVFYGSLAGGSMGTFVRLASYRVLKIPNDMSSTDAASIPVAYSTAIFAIIRIGRLRKGESVLIHAASGAVGQACIVLAQYIGARIFATAGTPAKREFLRETYGLPEEQIFSSRTPAFREGILSATDSQGVDVIVNSLSGNLLQETWDLTGDFGRFVEIGKRDLLQNSYLGMRPFDRNVTFSGVDLRTLFDRRPDEEKECLAELVDLVQRKVIVPIRPVTTVSISQLAIGLRKLQSGQNIGKIVVTMGPEDSVLADCPPALSVPYGQLLRPDATYLITGGTGGIGISLASWMVKNGALNVVLLGRSGSSRPEVKTLLEQYDGTDVQMRAVACDVGSRTELVSALRSIKDLPPVSGVVHGALYLHDSLLANATYDDWQNITRPRVQGAWNLHELLEDVDFFIALSSFVGAAGNLGQGIYAGTATYFDAFARHRIARGKATVAMALPVVLDVGYAADKGLTEALKLSLGATLTEADLHIAVKGAIIGPSSNLNENGSAIAFRFSSGEDPNTLGWQYYHPLALAERVNAKKRNSEITSPDEDNDTALNGLRLANSGDPLLALLDALMDKVSSITMIERDEVEPDAPLAVYGLDSLVSVELRNWIRRETGIETPLPAITRAENLRALATYILSQMETSRKS

>Phain_OT5_Proseq9590

MPSSTTSVRTFTMPKSLNASLAAEPGNAKHASEEGRSAEGVKQVSESYSKDQKDPLSNQPKPKPGITFAAQDRLPKLPIPELEHSMTGYLKALRPLQSPREHAETQQAVEEFLKSEGPELQERLKKYATGKTSYIEQFWYDSYLNFDNPVVLNLNPFFLLEDDPTPARNNQVTRAASLVVSALSFVRAVRREELPPDHIRGTPLCMYQYSRLFGTARVPTENGCHIGQDPDAKHIVVLCHGQFYWFDVLDDNSDLIMTEKDVSINLQTIVDDAQQTPIQEAAKGALGVLSTENRKIWSGLRDTMTRKEGSNNADCLGIVDSALFILCLDYTEPASGAELCQNMLCGTSSVEKGVQVGTCTNRWYDKLQIIVCKNGSAGINFEHTGVDGHTVLRFASDLYTDTILRFARTINGGAPSLWASTSPDPSKRDPASFGDVNTTPHKLEWDMVPELSIALRFAETRLADLIQQNEFQTLDFAAYGKNFITSMGFSPDAFIQMAFQAAYYGLYGRVECTYEPAMTKVYLHGRTEAIRSVTPESVDYVQTFWAENPPQQKVDTLKKACQKHTANTKECAKAQGCDRHLYALYSVWQKALDEDGAEAASSYGSNGYSSPIDGGSERDPSSIVGSPNRNSVLSSDGDDVVSLPSRYRNNSSPSRPGQQNTMPLLFADSGWDKLNNTILSTSNCGNPSLRHFGFGPTTGDGFGIGYIIKDEGISICASSKHRQTKRFVDAIESYLLEIRRVLRTTQRRGTSPTASRAREATASRPKPGSRLKSRGRVIRATEGKKTPGAMTPVEDSTVGSDDEGLGGCKSQISNSCHVNMDWDVNLQVWIEADDTRVDGFFDAGMLLQALKARGEGPGDDAKAPEARRREIGKKLRLSEY

>Phain_OT5_Proseq984

MAMDREERHLLSAAFFCPQSRAPDEEYLAGLHSFLQQNQYGQILLQEILDLESIWTIFENARDDVRALSQGPVYIDILRGWAANGESHRLSEVRSGIVALPLLIILQIGQYLRYLEFQGLAHQEFLAEIRHGGAQGYCGGLPPAIALACARDETEVVKNAGIAMRILLGIGAYGEAADEGNGTGTTTLALRLKYEGQGDEFARLFQGTHVSAITDPKSVSMVGPAQKLEEVFNYARAQGLQIQKMDIRGKVHNPENKDLAIELCQLCNETPCLQLPEASALQVPVRSNRSGEKLLHGSLTEEIVTTILASRCDWYVLLNKVAEDLAASKQPAHTFVIFGLNDCVPMSPFHRQRLQSTKFQAHSLIEKVRKPKSHDNLPLDSSFFQDSAIAVVGASCRLPGADNLEELWDLLASGSDRHQEVPADRFDLHGSFRASQSGGFTAGRKFYGNFIDDVQRFDNSFFGINPREAANLDPQQRILLELSFEALDASGYLSKHRREAGDNVGCFIGASLVEYLDNTNAHGPTAYTATGTIRAFLCGRLSHYYGWRGPSEVIDTACSSSGVAINRACKAIQTGECNMALAGGINIITGINNYLDLGKAGFLSPTGQCKPFDQSADGYCRSDGAGLVVLKKLKQAIIDGDHIMGVIPGIATNQGGLSASITVPDSGAQQALYQRVIQQSGLQPDQVTYIESHGTGTQAGDPLEMDSIRSVFGSPSRSDTAYVGSIKGNIGHCETAAGVASLLKVLAMLKHGQLPPQANHQRLNPKIPSLREDSLDITQSLRPWDAPFRAALVNSYGAAGSNCALLCCEMPQQESNKSHATDSELSFPFMLSAASQETLLDSVRVLGAHLRETSTNLNIGDISFTLNEKRKLHKYCMSTTSTSLDDLARQLSSLEPSDCFEFCQSSKPIVLCFSGQSSNKVALDKTLYESFPGFRYFIDACDAEIQALGFGSILPAIFQKEPIADVVILQCSIFAMQYACAQCWVESGLKISAIIGHSLGELTALAVSGVLSLSDSIKLVASRGRLIESQWGPEKGAMLALQCDMQGFEEISVHLKRSSQNGGLEIACHNAPASLVAVGTSATIDAVEDLLRTEPNLQKIRSQRLETSHGFHSALVEPILADLATISGSLTWNEPKIPLEVCTAEKFQSMQNYSVSSHAREPVFFSNAVRRLENSLGACIWLEAGVDTPIISMVRRASKQPDIHNFHAMKMQGERKYVDAVSSIWSALWKSGVSSTYWGFLPRHARQYKQIWLPPYQFKKTPHWRENVDRVIEMQQKLSTSDSATASVDLLQALPQLITRRAATGEAPGVAEFLVNTQSERFRKIVGGHAVRERPLCPASLYMECATMAIQLLTGDTKDASLVFEKVEFHSALGLDSGNEVVIRLEEVSNGQSWKFNIRSSMPTKPKPRQTSHGNGIISRDPNHIPSMFQRLVSGPTERLEKKEDAEKLMSTRAYGLFALVVDYAPFFQGIHAITLDDWEAIATISLPENQPNREESTSWKICDTVTIDSFIQVVGLLMNSSDVVSRGEVMVMVGIERAVISPACKMDEKKSWQVYAKFSFNQGQPIGDVFVSSPEGELVAMLCGCRFTKIQISKLEKALDSANSTVPLGVTPRKETPRNELSAGSITTSDGFMTPATSTPAQDSNDSVLRDLIAEYTGVNKLDISQDTIFADLGLDSLASVELVSELLSKFGLVITSDDLVTSTLHSLNQALGISSSHPVNIKSKKPRTGDYVNPNSIASPLYDLPEQESDGRRQQFLQILAEISGAKLEDIEPPNALADLGIDSLSSVDLKQELEDAFSERLDDFDLDCTVSELSTRLKINLSKQRALDLSLNLPRTGVAVPSSAPQLKENAVLPNPFDALKQSDTYFNASANKQGFLRYWSDVAPLQDELLLAYIVEGFCTLGVDFSRILPGNYVPQVPHVGQKYDKLMKRLWEILQKHHIVFIDEEGDVIRGSCPIDSRSSSQLAESFRAQFPGYEHETNLISLTGPRLADCLSGKADPVSIMFGSPSSLKIMENFYGQSPMMSTLTEQLVIFMTTLLHNQDSSQPVRILEVGAGTGGTTKRLAEALDADGIKAQYTFTDISPSLVAKAKNKFKQYPWIEFATFNLEKEVPAAFQNRFDVVVSANCVHATTNRTSSCRRLREVLNEDGFIVLSEVTRVMDWYDICFGLLDGWWLAEGRNAYPLQPAEAWMSTFEAAGFVSTGYSHGPTLEANSQQLLVASKKPWDTPAIMEIIPDTPHGQNGAYSMETMTYKEVGNVQIHADIYFPLRTPPSPMPIALMIHGGGFMTLSRKAIRPIQTQHLLANGFLPVSIDYRLCPEINIIDGPMADVCDAYKWAQTSLPEIASQHGITVDQTKVVVVGWSTGGHLAMSLGWTAKMAGLQPPIAVLSFYAPVDFESEDLDANRLSELPDRSMSMERIMAALPTTPITNYSSAKADSTGLGWVRPGDPRSELVMALFKEGIGLPLLLNGLPDTTTPSSEWFSRPSPAQAASISPLAQIRLGAYDTPTFIIHGTGDQIAPFAGAESFVVELRERGVRHGFWPLEGLDHIHDLRLRPGSEEWNTQVGPGYQFLFDVVRGHA

>Phain_OT5_Proseq9876

MFKYLLVAGLLPTIFAAPTPQMDYGENSGPVGGITAPVPTVTATSGSLYGPKSLLGEVAQPSPVSGGDSATVSNYPLVNGQEADSDLGLYLDFNSVENPQPLRGEGGQTDPGPRTYAYEKLNPDIFAPPGTDSGDVPQLQWPLGLSHNRFGSGKQAGWARQQNTDNLPIATAFAGVDMRLAPHAYRELHWHTANEWSLMLKGSVRLAAMNENGESFIDDISAGDVWFFPAGVPHSIQALDEGCEFLLVFDDGSFSEDSTSLVSELFERNPKEVLSKNLQTPISAFDDLPDGQLYIFNGTPAPANISVQNQTGPAGILPYDQSYTYHWSEQQPYETPGGSVKILDSTTFPIASDFAVALVTVQPGAMREMHWHLSSDEWNYFLSGSARITVFSAPSSSRTFDYTAGDIGYIPVTAAHYIENTGTEDVIFLEVLKQPKFTDISVAQWLAITPKQVVKDTLHLPDETLEGLPREKTYLKPGNRNMTALAADPNGTAAYEALD
